# Effects of mosquito control using the microbial agent *Bacillus thuringiensis israelensis* (Bti) on aquatic and terrestrial ecosystems: a systematic review

**DOI:** 10.1186/s13750-023-00319-w

**Published:** 2023-11-22

**Authors:** Magnus Land, Mirco Bundschuh, Richard J. Hopkins, Brigitte Poulin, Brendan G. McKie

**Affiliations:** 1 The Swedish Research Council for Environment, Agricultural Sciences and Spatial Planning (Formas), Box 1206, 111 82 Stockholm, Sweden; 2 iES Landau Institute for Environmental Sciences, RPTU Kaiserslautern-Landau, Fortstrasse 7, 76829 Landau, Germany; 3 The Natural Resources Institute, University of Greenwich, Medway Campus, Central Avenue, Chatham Maritime, Kent, ME4 4TB UK; 4 Tour du Valat Research Institute for the Conservation of Mediterranean Wetlands, Le Sambuc, 13200 Arles, France; 5 Department of Aquatic Sciences and Assessment, Swedish University of Agricultural Sciences, P.O. Box 7050, 75007 Uppsala, Sweden

**Keywords:** Diptera, Biocontrol, Indirect impacts, Non-target organisms, Chironomidae, Whole-ecosystem effects

## Abstract

**Background:**

The bacterium *Bacillus thuringiensis serovar israelensis* (Bti) is commercially produced in various formulations for use as a larvicide worldwide, targeting especially the aquatic larval stage of mosquitoes. However, there is a concern that repeated Bti treatments may have both direct and indirect impacts on non-target organisms (NTOs) and the ecosystems they inhabit. This review evaluates the evidence for such impacts.

**Methods:**

Literature was searched using six bibliographic databases, two search engines, and on specialist web sites. Eligibility screening was performed in two steps on (1) title/abstract, with consistency among reviewers assessed by double-screening 557 articles and (2) full text. Articles included after full text screening were critically appraised independently by two reviewers. Disagreements were reconciled through discussions. Key parameters of included studies are presented in narrative synthesis tables, including risk of bias assessments. Meta-analyses comparing treated with untreated ecosystems and using either the raw mean difference or log response ratio as effect size were performed.

**Review findings:**

Ninety-five articles covering 282 case studies were included in the review. From these, we identified 119 different response variables, which were divided into nine outcome categories. Most studies investigated NTO abundance or life history (reproduction related outcomes), but diversity and community composition are also well represented as outcome categories. The studies are highly variable in methodology, rigor, and spatio-temporal scale, spanning 1 day to 21 years and from < 1m^2^ to > 10,000 m^2^. Our metanalyses revealed a consistent negative effect of Bti treatment on abundances of Chironomidae and Crustacea, and also on chironomid emergence, although from a more restricted set of studies and regions. For most remaining response variables, we judged meta-analysis unfeasible, due to low study numbers or insufficient reporting of methods and results.

**Conclusions:**

There is now a significant body of studies documenting effects of mosquito control using Bti on NTOs or other ecosystem properties, especially associated with negative effects on Chironomidae, as apparent from our meta-analyses. Accordingly, we suggest the potential for negative NTO or other ecosystem effects of Bti treatment should not be discounted a priori. Once a decision to proceed with Bti treatment has been taken, priority should be given to a well-designed program of ongoing monitoring and assessment. The paucity of rigorous studies conducted with low bias risk for most response variables undermines our capacity for evaluating how common many of the effects documented might be. Future research would benefit from a rigorous and well-replicated approach to studying Bti impacts in semi-field mesocosms or in the field, combined with a greater rigor in reporting key methodological details. A greater focus is needed on understanding the environmental factors which regulate the wider effects of mosquito control using Bti on NTOs and ecosystems, to enhance our capacity for predicting where and when Bti is most likely to have additional, negative and indirect ecological impacts.

**Supplementary Information:**

The online version contains supplementary material available at 10.1186/s13750-023-00319-w.

## Background

**Fig. 1:**
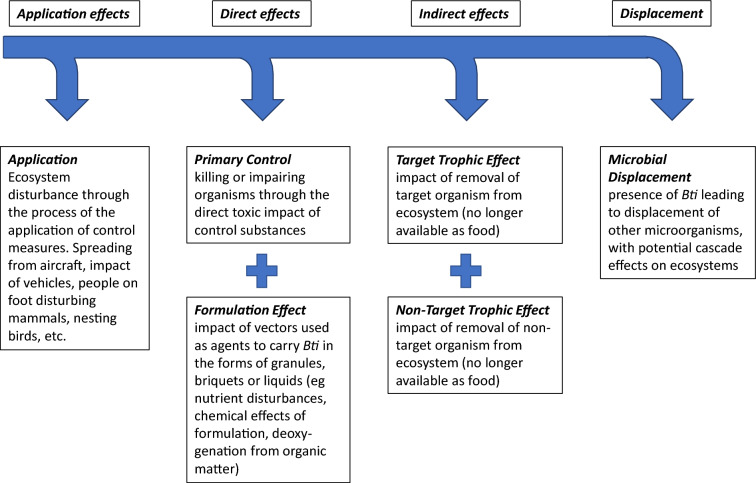
Conceptual model of ecosystem effects at different levels associated with insect control. In this systematic review we are primarily looking at direct effects on NTOs and abiotic parameters, indirect effects, and displacement

The bacterium *Bacillus thuringiensis serovar israelensis* (Bti) is commercially prepared in various formulations, including as liquid, water dispersible granules, powders, and pellets, for use as a larvicide all over the world, primarily targeting the aquatic life stage of mosquitoes. Bti produces crystal aggregations containing multiple toxins that disrupt the gut wall of organisms having an alkaline environment in their digestive tracts, as is typical of Nematocera, the suborder of Diptera (true flies) to which mosquitoes belong [1]. However, the control of insects, by whatever means, is not without disruption to the ecosystem in which the control measure takes place. There are a number of levels at which this disruption might occur (Fig. 1), and the disturbance of the ecosystem can be positive, neutral or negative for any individual species within the system. The first level at which a disturbance takes place is during the application procedure for the treatment [2] (Fig. 1). Insect control measures come in many forms, varying in application method from hand application to the use of vehicles or helicopters to treat large areas. For some organisms, such as mammals or nesting birds, the very presence of a human, a vehicle or aircraft provides a possible negative impact [3], although the disturbance may be transient and not have any measurable effect on the organisms concerned.

The next level of ecosystem disruption is associated with the direct effects of the control agent itself (Fig. 1), applied to reduce the population of the target organism(s). Additionally, any control measure applied may also have a direct effect on non-target organisms (NTOs). Bti, at recommended application rates, typically causes 90–100% mortality of the target organism’s larvae, with the presumption of limited or no direct impacts on other aquatic and terrestrial species [4–6]. The generally low level of NTO mortality caused by Bti contrasts strongly with alternative control agents such as chemical insecticides [7]. Non-target organisms showing some susceptibility to Bti are, like mosquitoes, members of the Diptera suborder Nematocera. In particular, non-biting midges (Chironomidae) are known to be susceptible to Bti [8–10], albeit sometimes at doses greater than those recommended for mosquito control [11]. Nevertheless, direct impacts of Bti on chironomid [12, 13], and on other types of NTOs including invertebrates [14], have been observed in some field studies applying Bti doses used in operational mosquito control. Further direct effects on ecosystems can arise from characteristics of the product used to deliver Bti in the ecosystem (Fig. 1). Different Bti-based mosquito control products vary in the range of additional compounds and materials used to facilitate the function of the agent in the field. Some products consist of granules or pellets (which include oils to bind the particles), whilst others deliver Bti as part of a liquid, often containing additional chemicals to enhance the delivery and action of Bti. The composition of the formulation agent can have a direct impact on NTOs in the ecosystem, either by toxicity or by modifying the nutrient balance of the affected habitat [15].

At the third level of effects (Fig. 1), large-scale population reductions of a target or non-target organism may cause broader disruption in food webs, including indirect effects spanning several trophic levels. Such indirect trophic effects occur when the direct effects of Bti on the density or behaviour of one or more organisms (target or NTO) affect further organism groups and the structure and function of food webs [16]. Thus, even when Bti has minimal direct effects on NTOs, it might be associated with knock-on effects on other ecosystem properties, including biodiversity and ecosystem functioning. This may be reflected in changes in the number and identity of species within trophic levels, the number of trophic levels, or ecosystem processes such as resource consumption and biomass production [17]. For example, a reduction of adult mosquito biomass of 90–100% following Bti application may remove an important food source for other organisms. Indeed, evidence from a longer-term study indicates that reductions in the biomass of emerging nematoceran dipterans (mosquitoes and chironomids) from wetlands treated with Bti alters the diet of birds, in turn reducing their breeding success [18]. Indirect effects might also result in positive outcomes for some organism groups in some circumstances. For example, reductions in the abundance of biting insects might relieve vertebrates from the negative effects of blood loss and the parasites and pathogens that blood feeding insects often spread [19, 20]. Fourthly and finally (Fig. 1), when a biological agent is introduced to an ecosystem there is the possibility that it becomes established and displaces other organisms. In the case of Bti, this has been promoted as a sustained presence yielding the advantage of “perpetual control”, but the long-term impact of disrupting the microorganisms in an ecosystem requires evaluation [21].

In Sweden, Bti has been applied on a large scale almost yearly since 2002 (not in 2004, 2013 and 2017) in the form of the commercially available granular formulation VectoBac G® (Valent BioScience, USA) (www.mygg.se). The applications have taken place in the lower Dalälven River area to control mass outbreaks of the floodplain mosquito *Aedes (Ochlerotatus) sticticus* Meigen [6]. During 2002–2011, the number of applications ranged from 0 to 4 times per year between May and August, at an average dose of 13–15 kg per hectare (2.6–3.0 × 10^9^ ITU/ha), with treated areas ranging from less than 100 to more than 3500 ha [6]. This control program has been highly effective at reducing abundances of flying *Ae. sticticus,* evident not only in individual mosquito outbreak years [6], but also possibly in a tendency for a longer term decline in population densities (www.mygg.se). Ongoing monitoring of impacts on NTOs in the lower Dalälven suggests limited or no negative consequences for Chironomidae [22] or other organism groups [23]. Assessment of indirect ecological effects in the lower Dalälven are scarce. Native bacteria (*Bacillus cereus* group) abundances appear little affected [21]. However, increases in occurrence of Bti itself [21] in the wetlands, and changes in heterotrophic protozoan communities [24] indicate shifts in the microbiota of treated wetlands. This, together with changes in beetle assemblages [23] and the trophic length of ground-based food webs quantified based on isotopic biomarkers [15], point towards the potential for repeated application of Bti to alter food-web properties. Finally, the marked reduction in abundances of flying *Ae. sticticus* adults could have wider consequences for other organism groups [25], which might be either positive (e.g., reduced harassment and blood-feeding on vertebrates) or negative (a reduction in food for mosquito predators), but these have not been investigated.

The Swedish Environmental Protection Agency (Swedish EPA) judges the risk of unwanted ecological impacts of large-scale and repeated Bti treatments to still be unclear and does not find legal grounds for permitting Bti treatments in the Natura 2000-designated areas of the lower Dalälven catchment. However, in such cases the Swedish EPA can transfer the decision regarding whether permission should be granted to the Swedish government [26]. The Swedish EPA has expressed a need for a synthesis of the available evidence for ecological impacts of mosquito control using Bti. To refine the review question and design a systematic review as relevant as possible, the Swedish research council Formas arranged in 2018 a stakeholder meeting where representatives from the Swedish EPA, county administrative boards, municipalities, non-governmental environmental organisations, and mosquito control associations discussed the significance of the question, potential sources of evidence, study inclusion criteria, and study quality aspects (See Additional file 1). Although the initial motivation for this review arose from the specific situation in Sweden, evidence from all over the world are compiled. Accordingly, the findings of the review are expected to have broad relevance to all regions of the world where biological control of blood feeding Nematocera is undertaken.

## Objective of the review

The objective of the review is to answer the primary review question: “What are the effects of mosquito control using the microbial agent *Bacillus thuringiensis israelensis* (Bti) on aquatic and terrestrial ecosystems?”. The key elements of the primary question are defined by the following PECO (Population/subject, Exposure, Comparator, Outcome):

- Population/subject: Aquatic or terrestrial ecosystems.
- Exposure: Bti treatments for control of mosquitoes.
- Comparator: Reference ecosystems not exposed to Bti or any other Nematoceran control agent/intervention.
- Outcome: Outcomes related to food web structure and function.

Here we are not primarily concerned with the direct effects of Bti Cry toxins on target organisms, which are reasonably well understood [1]. Accordingly, studies that only report changes in the abundance or any other property of target organisms without data on changes in other ecosystem properties are not considered relevant for our review. Rather, we are interested in examples where unintended consequences on other ecosystem properties are documented. Such unintended effects might include changes in the structure (biodiversity, trophic levels, functional group composition), resource base (e.g., relative importance of different plant types and/or detritus) and functioning (changed ecosystem processes including those underpinning biogeochemical cycles) of food webs.

The key elements are further defined in the subsection on eligibility criteria. In addition to the primary question, the consulted stakeholders were interested in answers to the following secondary questions: (1) Are ecosystems more affected by long-term and repeated Bti treatments? (2) Is there a clear dose–response relationship? (3) Do landscape characteristics (e.g., in terms of floodplain characteristics, vegetation type, species composition etc.) affect the type or size of effects? (4) Are observed effects transient or long-lasting?

## Methods

### Deviations from the protocol

The most important deviation from the protocol [27] is that exposure to Bti treatments for control of other nematoceran Diptera than mosquitoes is not eligible. The reason for this is that other target organisms, such as black flies, breed in fast flowing lotic ecosystems [28] that require higher Bti application rates than the still-water habitats typically occupied by mosquitoes [9]. Control of other nematoceran Diptera than mosquitoes was not considered as relevant for the consulted stakeholders. We have also decided to not include other control methods or control agents as comparators due to a paucity of such studies. As stated in the protocol, articles written in Spanish were included during the literature screening process. However, at the screening and data extraction stage we judged it necessary to prioritise articles in English, German, and French, due to time and translation constraints. In the protocol we wrote that the search results could be split between two reviewers for screening but only one reviewer screened the vast majority of the search results (see below for details). Another deviation is that the critical appraisal of included studies is not based on the original statistical evaluation (e.g., ANOVA) performed by study authors, as there was too much variation in statistical approach and reporting of error and effect sizes. Rather, we have used effect sizes based on published raw data or mean values, variances, and number of replicates for each treatment group.

### Search for articles

Searches were made in August 2019 and in May 2022 in the bibliographic databases and platforms listed in Additional file 2, using the following search string:

(nematocera* OR midge* OR diptera* OR mosquito* OR vector* OR larv* OR “black fly” OR “black flies” OR biting OR chironom* OR culicid* OR simuliid* OR anopheles OR aedes OR ochlerotatus OR culex OR culiseta OR limatus OR uranotaenia OR psorophora OR mansonia OR armigeres OR trichoprospon OR coquillettidia OR tripteroides) **AND** (bti OR israelensis OR vectobac* OR Introban* OR biorational* OR biopesticid* OR biolarvicid*).

The asterisk (*) is a wildcard representing any number of characters. The search string was adapted to valid syntax in each database. An evaluation of the search string was shown in the protocol for this review [27]. No constraints regarding time, document type, or language were applied.

Searches were also performed using the internet search engines Google and Google Scholar in advanced search mode. In these searches the simplified search strings below were used.

- Mosquito AND effect AND (Bti OR israelensis OR vectobac)
- “Black fly” AND effect AND (Bti OR israelensis OR vectobac)
- Thesis AND Bti AND mosquito AND (MSc OR PhD)

Searches in Google Scholar were performed using Publish or Perish [29] and for each search string the first 300 articles were screened. Search results in Google were restricted to pdf documents, and for each search string the first 100 were screened. The same search strings translated to Swedish, German, and French were also used (screening was limited to the first 100 hits in each language). Websites of relevant specialist organisations (listed in Additional file 2) were also searched. An evaluation of the search strategy was reported in the review protocol [27].

### Article screening and study eligibility criteria

#### Screening process

Search results were exported to EndNote X9 where 10,583 duplicates were removed. All unique articles were then exported to Eppi Reviewer [30] where screening on eligibility took place in two stages. At stage one the screening was based on titles and abstracts and at stage two it was based on full text. To test the eligibility criteria, 557 articles were screened on title and abstract by five reviewers (authors of this article) independently. Following an analysis of all disagreements (Kappa values 0.65–0.69) and amending the preliminary eligibility criteria with some important clarifications, we reached consensus on the interpretation of the criteria. From that point the bulk of the search results were screened by one reviewer (ML), while BP and MB screened papers in French and German, respectively. At stage one there were three options: 1) include, 2) exclude, and 3) probably exclude. All articles coded with option 3 were screened by an additional reviewer, after which a consensus decision was made. Reviewers were allowed to participate in discussions regarding the eligibility of their own papers against the background of this systematic review, but they were excluded from making final decisions on eligibility. All articles included after this process were at stage two screened independently by two reviewers using full text, and any disagreements were reconciled through discussions.

#### Eligibility criteria

Although effects of a certain change in the food web (e.g., reduction in mosquito biomass) may be the same regardless of what factor caused the change in the food web (e.g., Bti or some other control method), we include only publications where Bti itself is the primary agent of change in densities of the target organism. We have not applied any geographic limitations. Even though our focus is on temperate and boreal systems, ecosystems are complex and insights in, for example, tropical systems can inform decision making in other climatic regions when posing more general questions as we do here. The following criteria have been used when assessing relevance and deciding on inclusion or exclusion of studies.

##### Eligible population/subjects

Aquatic or terrestrial ecosystems.

##### Eligible exposure

Bti treatments for control of mosquitoes or for Bti effect assessment designed for mosquito control. All forms of Bti products (granular, liquid, sterilised, non-sterilised etc.) and application techniques (ground-based, air-borne etc.) were eligible.

##### Eligible comparator

Reference ecosystems not exposed to Bti or any other Nematoceran control agent/intervention.

##### Eligible outcomes

Any outcome related to ecosystem processes or ecosystem function were deemed eligible. This broad definition of eligible outcomes means that a wide range of response variables have been included in this review. Response variables have been grouped into nine outcome categories, shown in Table 1. It should be noted that although the target species (mosquitoes) are part of the ecosystem, outcomes pertaining to those are not eligible. Also, vector-borne pathogens are part of ecosystems, and studies documenting changes in abundances of such pathogens in aquatic or terrestrial food webs may be eligible. However, changes in incidences of pathogens among human populations are beyond the scope of the review question.

**Table 1:** Outcome categories and example response variables^a)^

Outcome category	Example response variables
Abundance	Number of individuals, density
Diversity	Species richness, evenness, diversity indices
Community composition	Bray–Curtis distance, species turnover
Species traits/Feeding groups	Body size, growth, longevity
Life history	Clutch size, reproduction success, emergence success, number of generations per year
Food web structure/biomarkers	Changed food sources, isotopes
Changed ecosystem processes	Leaf decomposition, plant productivity
Environmental data	Nutrient concentrations, pH, suspended solids, dissolved oxygen
Persistence/fate^b)^	Number of spores, toxicity of crystals

^a)^ The outcome categories used in this review and how to allocate the response variables among these are to some extent arbitrary, but the review team members agreed that this categorization was a pragmatic way to structure the data and help the readers to get an overview of the results

^b)^ Not including cases where short-term persistence was quantified as toxicity against target species, for example using the response variable mortality of mosquito larvae or abundance of mosquitoes

##### Eligible types of study design

Field-based studies or mesocosm studies using field-sourced communities or laboratory studies including quantification of ecological interactions, using before-after (BA), control-impact (CI), before-after control-impact (BACI), or randomised control trial (RCT) study designs.

##### Eligible languages

English, German, French, Swedish. Thirty-two articles screened at full-text level were in other languages (predominantly Russian, Portuguese, and Chinese, see Additional file 4) and were not included.

### Study validity assessment

#### Internal validity

The internal validity assessment was based on (1) risk of bias and (2) data quality based on sampling strategy and other factors that not necessarily cause bias but may in other ways make the study less sensitive or selective to true differences in outcome measures between a treatment and a control group. For most outcomes one key challenge is that many systems, especially periodically flooded wetlands, are often highly heterogenous in space and time. It is therefore important to consider the ability of each study to account for such heterogeneity and provide results that are representative for the whole studied system. Key recorded parameters include study duration, size of study area, spatial and temporal sampling density, level and method of taxonomic identification, and method for quantifying Bti spores in the field.

We developed a “risk of bias tool” specifically for this systematic review based on the assumptions that the main sources of bias are selection bias, performance bias, detection bias, and reporting bias. When assessing the risk of bias, we acknowledged that the susceptibility to selection and performance bias may depend on the experimental design. The assessment of these biases has thus been guided by parameters detailed in Table 2 and Table 3. Risk of selection bias may be high when treatment and control areas were not selected randomly. However, given the potentially large degree of heterogeneity in many target species habitats, randomisation in allocation of treatment and control areas requires a relatively large sample size (high number of replicates) to balance all heterogeneities and confounding factors between groups of treatment and control areas. If the sample size is low, it is possible that one of the groups *by chance* are characterised by, for example, a larger proportion of open water. The risk for this kind of selection bias may be lower in studies where comparable study areas are matched in pairs but treatment allocation is randomized *within* each pair. Performance bias may occur when study groups are managed differently. For example, some areas may be more visited by tourists or more susceptible to extreme weather events than others. Ubiquitous time-related trends, and contamination of study groups where treatment and control areas are not isolated from each other may also form sources of performance bias. Detection bias may occur if different sampling or measurement methods or if inadequate methods are used for different groups. For example, the number of insects collected by a certain device may not only depend on the abundance of the insects but also on the activity of those insects, and the activity of the insects may vary with a range of local and temporary conditions. In such cases, even if the same sampling device is used for all study groups and across all replicates, the efficiency of that sampling device may vary when employed at different times or locations.

**Table 2:** Guidance for assessing risk of selection bias for different study designs

Study design	Treatment and control areas	Randomisation of treatment allocation	Risk of selection bias
BA	Treatment and control in same area	N/A	Low
CI	Independent groups (spatially independent replicated controls and exposure areas)	Yes	Probably low / Moderate
		No	High/Probably high
		Unclear	Unclear
	Matched pairs (one control and one exposure area in each replicate)	Yes, within pairs	Low
		No	Probably high
		Unclear	Unclear
BACI	Independent groups or Matched pairs	Yes or No	Low/Probably low

**Table 3:** Guidance for assessing risk of performance bias for different study designs

Study design	Source of performance bias	Reported or suspected	Risk of performance bias
BA	Confounding factor or time-related trend	Yes	High/Probably high
		No	Low/Probably low
		Unclear	Unclear
CI and BACI	Confounding factor or contamination of study groups	Yes	High/Probably high
		No	Low/Probably low
		Unclear	Unclear
	Time-related trend	Yes or No	Low (N/A for CI)

For each individual study, the overall risk of bias was governed by the source of bias carrying the highest risk. For example, if at least one source of bias was judged to carry a “Moderate” risk while none was judged to carry a “High” or “Probably high” risk, the overall risk of bias was judged to be “Moderate”. Similarly, if any of the sources was judged to be “Unclear” while none was judged to carry a “High” risk of bias, the overall risk of bias was judged to be “Unclear”. The overall risk of bias was always judged to be “High” if at least one source of bias was judged to carry a “High” risk of bias.

#### External validity

External validity, i.e., the degree to which the studies are appropriate or applicable for answering the review question in a particular context, was partly assessed based on the eligibility criteria during relevance screening. Nevertheless, the eligibility criterion for exposure needed further consideration. The criterion states only that the exposure should be to Bti treatment for mosquito control. However, to be able to reliably conclude that a treatment does not cause side-effects, the study should demonstrate that the level of Bti treatment indeed was sufficient to obtain the *intentional* effect (in this case a significant decrease in mosquito abundance). It is open to debate how much lower the target species abundance needs to become to regard Bti treatment as successful, but it has been argued that as much as 95% of mosquito larvae need to be killed to achieve a significant decrease in the perceived nuisance by people in Sweden [6]. Accordingly, a statistically significant decrease in the abundance of mosquito larvae alone is not enough to evaluate the success of mosquito control. However, it is also difficult to define successful mosquito control efforts by a fixed number that can be applied across all environments, target organisms, and social contexts. The efficacy of Bti is variable over time and location, being influenced by water temperature, sunlight, vegetation coverage, and density of filter feeders (mosquitoes and others) [31]. Accordingly, it is difficult to a priori define how much the target species abundance must decrease to make an assessment of side-effects meaningful. We have chosen a pragmatic approach where we did not exclude any studies based on target mosquito species or achievement of intentional effect, but where we record these parameters for each study and make a comment on the external validity in a Swedish context. The assessment of external validity was more focused on type of study, type of habitat and ecosystem, and the Bti application rate. Application rate is here defined as dose (amount/ha) per treatment. In 2018 the maximum allowed application rate in Sweden was 15 kg Vectobac G®/ha per treatment, and the maximum allowed treatment frequency was 4 treatments/year [32]. The active ingredient in Vectobac G® is *Bacillus thuringiensis subsp. israelensis* serotype H-14, strain AM65-52, at a concentration of 200 International Toxic Units (ITU) per mg. The maximum allowed application rate in Sweden is thus 3·10^9^ ITU/ha per treatment.

#### Coding for study validity assessment

Data on key parameters necessary for study validity assessment was entered in predesigned Excel data sheets (see Additional file 3). Critical appraisal and coding for study validity was carried out by all reviewers, and each study was critically appraised independently by two reviewers. The articles were randomly allocated to the reviewers, but no one was allowed to assess the validity of their own work. Disagreements were reconciled through discussions seeking to reach consensus among all reviewers. The assessments of internal validity were used to probe the general level of methodological rigour in the evidence base. They were also used for sensitivity analyses in which studies with a “high”, “probably high”, or “unclear” risk of bias were excluded from meta-analysis to evaluate how these cases altered the summary effect. The assessments of external validity were used especially in cases where several treatments (most often different Bti application rates) had been investigated in the same setting. We choose to include in the meta-analysis only the one treatment that had the highest external validity, i.e., the treatment closest to the recommended Bti application rate.

### Data coding and extraction strategy

Study metadata were recorded in the same pre-designed Excel datasheets that were used for critical appraisal (Additional file 3). Extracted metadata include bibliographic details, Bti treatment details (e.g., Bti product used, number of treatment years, application frequency, dose/treatment, size of treated area), study area details (e.g., coordinates, type of ecosystem and habitat, study area size, target species and target species mortality, average water depth at treatment), sampling details (e.g., start and end of sampling period, sampling frequency), funding body, and outcome details (e.g., measured response variable, unit). Bti treatment and study area details were mainly used for external validity assessments while sampling details were mainly used for internal validity assessments. The articles included for data extraction were randomly split in two batches and allocated to two reviewers (ML and MB). To check consistency between reviewers all 59 articles extracted by MB were double checked by ML. Where needed, consensus was reached through discussion. Data was recorded as reported in each study. If necessary and feasible, data were standardised at the analysis stage to allow for direct comparison among studies.

In many cases one article has reported more than one Bti treatment and one study. Here, a Bti treatment is defined by the Bti product used and the dose at which it was applied, whereas a study is defined as an investigation with a unique combination of article ID, study location, medium, Bti treatment, and outcome category. This means that one individual study may provide data for more than one response variable and more than one species. In the metadata Excel sheet, one column was used for each study.

Outcome data were recorded in a separate Excel file for each article, in which a separate sheet was used for each study. Outcome data for all reported time points were recorded. In cases where outcome data were reported in graphic figures, we used WebPlotDigitizer [33] to extract data.

### Potential effect modifiers/reasons for heterogeneity

In the review protocol we listed the following potential effect modifiers for consideration: (1) dose/treatment, (2) number of treatments/year (frequency), (3) biological active concentration of Bti, (4) average water depth of inundated areas, (5) duration of Bti treatment (number of years), (6) background biodiversity and community composition including non-target species sensitive to Bti cry toxins, and (7) formulation of Bti used. We have extracted data for all these factors except background biodiversity and community composition, which were rarely reported.

### Data synthesis and presentation

All studies included in the review are included in narrative synthesis tables in Additional file 6. There is one table for each outcome category. The tables provide bibliographic information including funding body, information on study characteristics, risks of bias, external validity assessments, and whether study results have been used in meta-analysis. When case study results have not been used in the meta-analysis a reason for that is given. The results of studies are discussed in the Data synthesis section, where the studies are grouped by outcome category and each response variable is discussed separately. The discussions are focused on (1) number of studies, (2) study validity (internal and external), (3) consistency of observed effects among studies, (4) size and significance of observed effects, and (5) relationship between the intensity of the Bti treatment(s) and the outcome (dose–response relationship). The last aspect is, however, not always easily evaluated since Bti (if not sterilised as required in Germany [34]) is a bacterium having the potential to sporulate [35, 36] and therefore its concentration in the environment may not necessarily correlate with the amount applied. Together, the above-mentioned aspects make up a basis for an assessment of the *strength of evidence* for each outcome category or response variable.

Quantitative synthesis through meta-analysis was performed where at least four studies were available that assessed comparable response variables. Raw mean difference (D) is used as effect size when the response variable is measured on a common scale across all studies (e.g., pH). In other cases, log response ratio (R) is used as effect size. The log response ratio was chosen over standardized mean difference since the magnitude of the former effect size is easier to interpret.

For studies with independent groups the raw mean difference, log response ratio, and their within-study variance estimates (${V}_{D}$ and ${V}_{R}$, respectively) were calculated as:

$$D={\overline{X} }_{T}-{\overline{X} }_{C}$$

$${V}_{D}=\frac{{s}_{T}^{2}}{{n}_{T}}+\frac{{s}_{C}^{2}}{{n}_{C}}$$

$$R=ln\frac{{\overline{X} }_{T}}{{\overline{X} }_{C}}$$

$${V}_{R}=\frac{{s}_{T}^{2}}{{n}_{T}{\overline{X} }_{T}^{2}}+\frac{{s}_{C}^{2}}{{n}_{C}{\overline{X} }_{C}^{2}}$$

where $\overline{X }$ is mean, s is standard deviation and n is number of replicates. T and C denote treatment and control group, respectively. The equations for calculating ${V}_{D}$ and ${V}_{R}$ assume that the two population standard deviations are not the same.

Studies investigating more than one treatment (in most cases different Bti application rates) often used the same control group for comparing all treatment effects. Rather than applying a multiple comparisons correction for potential inflated type-II statistical error, we choose to include in the meta-analysis only the treatment that has the highest external validity, i.e., the treatment closest to the recommended Bti application rate. However, we have also conducted meta-regressions with Bti dose as moderator, and in these regressions, we have included multiple treatments using the same control. In some studies, the same response variable has been investigated for several species or taxa groups. In such cases, we used only one species or taxa group to avoid risks of statistical dependence confounding our results. To obtain a conservative estimate of negative effects we select the one showing the least negative effect size unless stated otherwise.

Several studies have measured the response variable multiple times (time series) in the control and Bti treatment group. To address our secondary question regarding the time scales of effects (question 4), we have evaluated how large short-term effects may be, for how long effects persist after the last application, and what the overall effect integrated over time is. Therefore, separate meta-analyses have been produced for (1) the maximum negative effect during each study period, (2) the effect at the end of each study period, (3) the overall effect across the whole study period, and (4) the maximum positive effect during each study period. For meta-analysis of maximum short-term effects, we have included only studies reporting one or more discrete time points. For meta-analysis of end-of-study effects, we have included only studies reporting multiple time points, while for overall effects, we have used studies with all types of temporal coverage. In cases they were not reported, the integrated effects across the whole study period have been calculated by treating the time points as replicates.

In the meta-analyses, we used a random-effects model where the between-study variance (${\tau }^{2}$) is estimated by the Mandel-Paule estimator [37]. It has been argued that this estimator is better than the more commonly used DerSimonian-Laird estimator [38], and it has also been evaluated for log response ratios [39]. Where log response ratio is used as effect size, study weights are based on sample size in this systematic review. Bakbergenuly et al. [39] showed that this resulted in less bias of the weighted average compared to inverse variance weights. It should be stressed however, that in our case the sample sizes in each study (n) as well as the number of studies in each meta-analysis (k) are generally rather small, which increases the risk of biased weighted averages. Also, when the mean in either the intervention group or the control group is close to zero, the distribution of the log response ratio may not be normal. To test whether log response ratios provide valid and accurate approximations of effect sizes to be used in meta-analysis, we have used the diagnostic tests proposed by Hedges et al. [40] and Lajeunesse [41]. We used the *I*^*2*^-statistic [42] to evaluate the proportion of the observed variance that reflects heterogeneity, i.e., real differences between studies. To test the robustness of significant summary effects we calculated Rosenberg’s fail-safe N [43]. In cases where meta-analysis included more than 10 studies, we constructed funnel plots [44] to assess the risk of publication bias. Meta-analysis was carried out in R using the metafor package [45].

## Review findings

### Review descriptive statistics

**Fig. 2:**
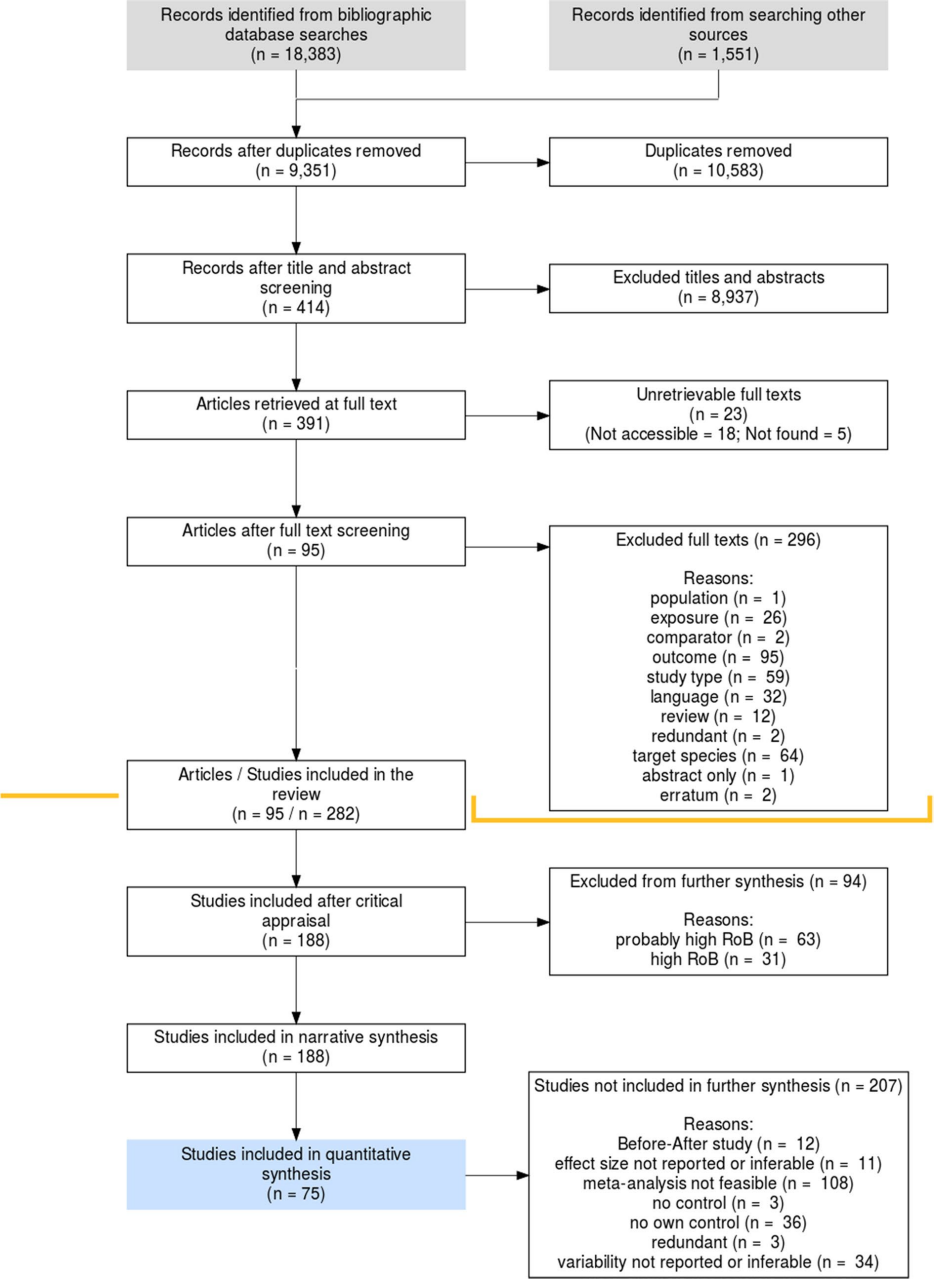
Roses flow chart illustrating the screening process. Some of the studies excluded after critical appraisal were included in quantitative synthesis. The influence of these on the summary effects were tested through sensitivity analyses

Our literature search resulted in 9,351 potentially relevant unique articles. None of these were found through the manually searched specialist websites. After the first screening at the level of title and abstract, 414 articles remained and after full text screening 95 articles with 150 Bti treatments and 282 studies were included in the review (Fig. 2). Additional file 4 provides a list of unretrievable articles and articles excluded during screening at the full text level with reasons for exclusion. A complete list of the included articles is also shown in Additional file 4.

**Fig. 3:**
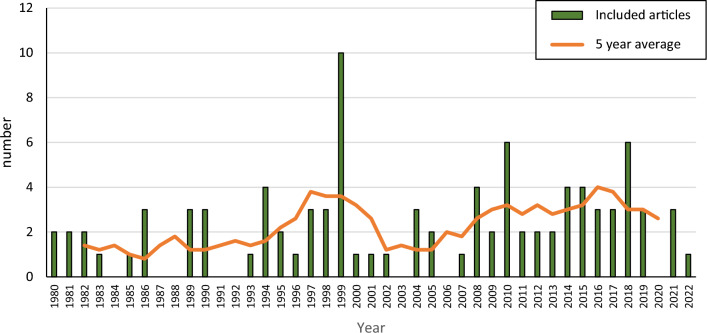
Number of included articles published each year

**Fig. 4:**
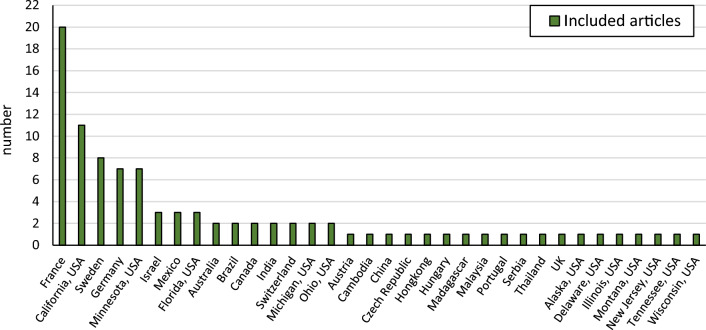
Number of included articles reporting on studies from different countries or US states. The greatest number of papers report studies from USA (32 articles), followed by France, Sweden, and Germany. The US states have been separated to achieve a similar level of spatial resolution and detail as for Europe

The included studies were published between 1980 and 2022, with a small peak in published articles during the late 1990s and a growing number from around 2005 (Fig. 3). The distribution of included papers across countries or states (USA) in which the studies were performed is shown in Fig. 4. The greatest number of papers report studies from USA (32 articles), followed by France, Sweden, and Germany. In total, studies from 23 different countries are included.

The included articles have in total reported 119 different response variables which we have grouped into nine outcome categories. The number of articles and studies reporting on each outcome category is shown in Table 4. Response variables designated to each outcome category are shown in Additional file 5.

**Table 4:** Number of articles and studies reporting on different outcome categories. Also indicated are the number of response variables within each outcome category

Outcome Category	Articles	Studies	Response variables
life history	24	43	14
abundance	52	95	12
diversity	21	30	12
community composition	15	32	10
species traits/feeding groups	13	21	32
food web structure/biomarkers	8	11	14
changed ecosystem processes	1	3	1
environmental data	11	26	16
persistence/fate	13	21	8

### Narrative synthesis including study validity assessment

Metadata and study validity assessments for all included studies are listed in narrative synthesis tables provided in Additional file 6. These tables also indicate if study findings were used in meta-analyses and, if not, the reason for exclusion. Given the high temporal variability of ecosystem properties in especially temporary wetlands, field studies with a BA design were excluded from meta-analyses as the risk of performance bias in those was judged to be high.

**Fig. 5:**
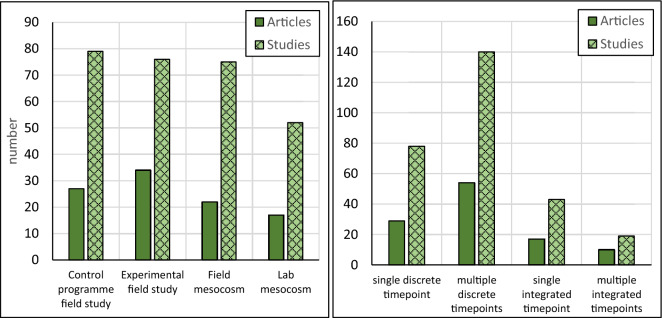
Number of included articles and studies within different study types (left) and temporal coverage of the reported results (right)

We have identified four types of studies: (1) observational field studies conducted in connection with ongoing Bti treatment programmes, often on scales > 1000m^2^, (2) experimental field studies on scales typically > 10m^2^, (3) field mesocosm studies on scales typically < 1m^2^, and (4) laboratory mesocosm studies. The distribution of study types among the included articles is fairly even, but the greatest number of articles reported experimental field studies (see Fig. 5).

The study duration of the articles included in our review varied from a few days up to 22 years. We have grouped the results reported based on their temporal coverage: 1) one discrete timepoint after Bti application reflecting the situation on one sampling occasion, usually 1 to 7 days after treatment, 2) more than one discrete timepoint after Bti application (i.e., a time series often running for several weeks), 3) one integrated timepoint reflecting the overall effect during the entire study period (i.e., samples were taken on multiple occasions but the results were reported in an aggregated form, often showing seasonal or annual effects), and 4) more than one integrated timepoint, often showing seasonal or annual effects over several years. Driven by the inherently different duration and time resolution of these studies, these result types should not be mixed in the same meta-analysis without consideration of how comparable they are in temporal coverage (in the Methods section we describe which results are included in what meta-analysis). The article and study distribution among the four result types is shown in Fig. 5.

**Fig. 6:**
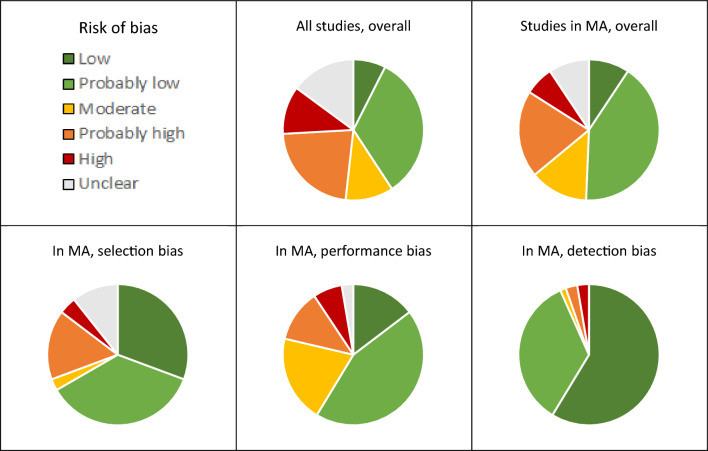
Assessment of overall risk of bias for all included studies and for studies included in meta-analyses (upper row) and risk of selection bias, performance bias, and detection bias for studies used in meta-analyses (lower row)

**Fig. 7:**
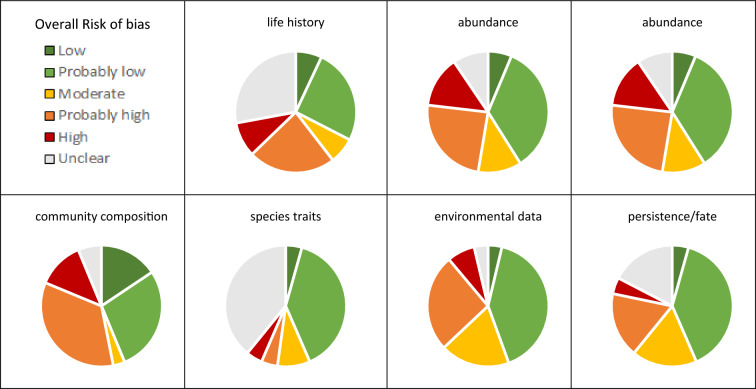
Assessment of overall risk of bias for all included studies within different outcome categories

Looking at all included studies, the overall risk of bias was judged to be low to moderate in 48% of the studies, “probably high” or high in 24%, and unclear in 28% (Fig. 6). The overall risk of bias is generally lower in studies used in meta-analyses (evaluated as low-moderate in 63% of the studies, as “probably high” or high in 18%, and as unclear in 19%). When examining different types of bias, we can see that the risk was highest for performance bias followed by selection bias and detection bias. The most frequent underlying reason for high risk of performance bias was a suspected presence of confounding factors or time-trends. The risk of bias varied also between outcome categories (Fig. 7). The overall risk of bias is highest in studies on diversity and community composition where it was judged to be low-moderate in only about 35% of the included studies, whereas the risk of bias was lowest in studies on persistence/fate and environmental data where it was judged to be low-moderate in 67% and 58% of the studies, respectively. However, for several outcome categories, the fraction of studies with *unclear* risk of bias is relatively large, calling for more transparent reporting in articles.

**Fig. 8:**
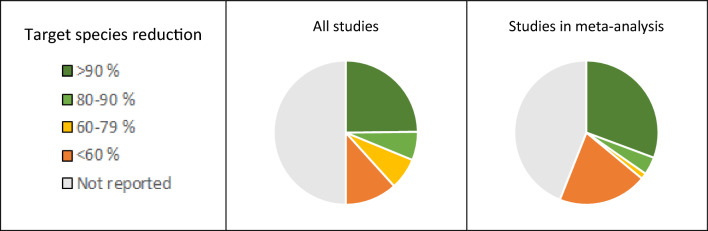
Reduction in abundance of target species in all included studies and in studies used in meta-analyses

**Fig. 9:**
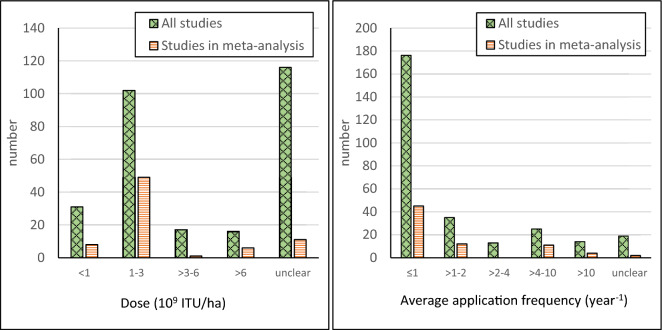
Number of included studies with various Bti doses (left) and application frequencies (right). For studies where treatments have been carried out for more than one year an average application frequency has been calculated

**Fig. 10:**
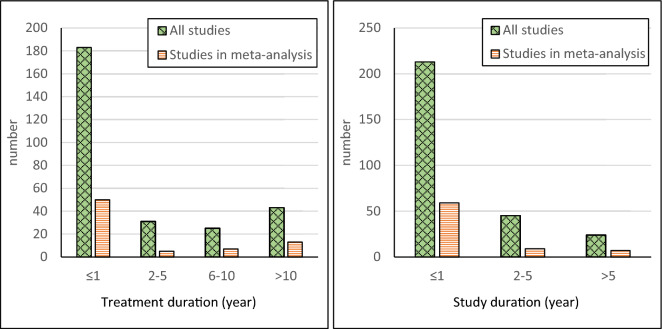
Number of studies with various treatment duration (left) and duration of the study itself (right)

The reduction in abundance of the target species is a key parameter for the external validity as any assessment of side-effects is meaningful only when the Bti treatment has been shown to achieve the intended effect. However, the reduction in abundance of target species is reported in only 50% of all included studies, with the degree of reduction reaching 90% or more in only c. 25% of the included studies (Fig. 8). The same is true for the studies included in meta-analyses. Another important parameter for the external validity is the Bti application rate and frequency. In Sweden, the maximum allowed rate of 15 kg Vectobac G®/ha per treatment corresponds to 3*10^9^ ITU/ha. A majority of the included studies, as well as of the studies used in the meta-analyses, have used a dose lower than that (Fig. 9). Most of the included studies are within the allowed range in Sweden also in terms of application frequency (max. 4 applications per year), see Fig. 9. As many mosquito control programmes run for several years in one area, the external validity of the studies is also dependent on the duration of Bti treatments. The treatment duration is however rather short in most studies; one year in about 65% of the studies and 5 years or less in about 75% of the studies (Fig. 10). Furthermore, although many studies have measured effects during periods of 30 days or more during one season, significantly fewer studies have measured effects over a time period of more than one year. Consequently, the available evidence base provides little information regarding long-term Bti treatments and how the effects on the ecosystems may accumulate over time.

### Data synthesis

For each outcome category, relatively few studies have assessed comparable response variables or have presented the data in a way that allows quantitative synthesis through meta-analysis. In this section we report the results of the meta-analyses we have found feasible and judged meaningful to conduct. To complement the meta-analyses and investigate whether additional evidence corroborate or contradict the analyses we also summarise the findings of studies that for various reasons could not be used in the quantitative synthesis. However, the results of such studies are not considered in cases where the risk of bias was judged to be “probably high” or “high” (information about those studies can be found in Additional file 6, Narrative synthesis tables).

The small number of studies in each meta-analysis makes it difficult to investigate interactions between moderating factors and to conduct sensitivity and subgroup analyses. However, in Additional file 7 we provide some key characteristics of the included studies (e.g., risk of bias assessments, study design, study type, study duration etc.), making it possible for the reader to get a quick overview of the validity of the evidence base. In Additional file 7 we further visualize the meta-analyses as forest plots for 1) the maximum negative effect during each study period, 2) the effect at the end of each study period, and 3) the overall (time-integrated) effect across each study period.

#### Life history

**Fig. 11:**
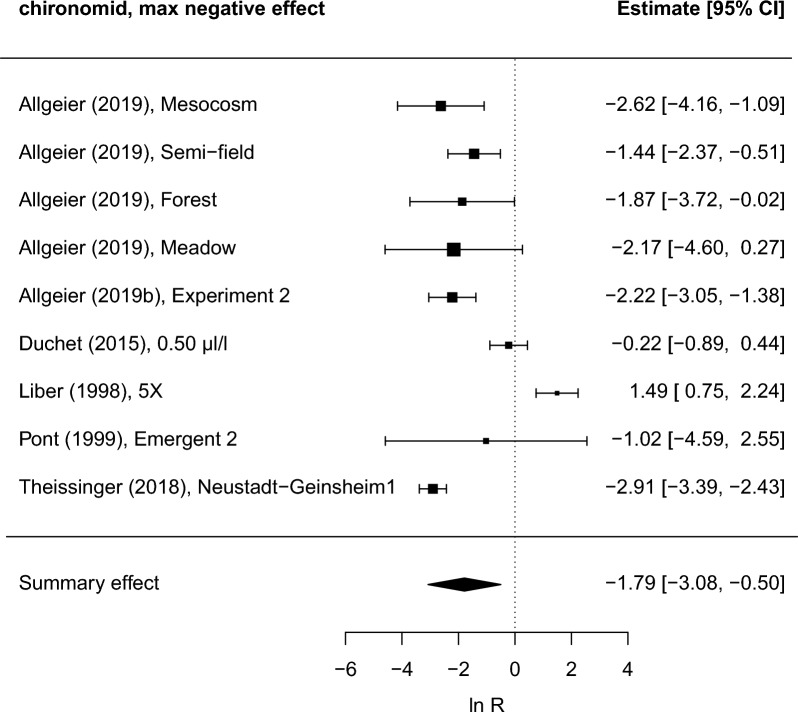
Forest plot showing maximum negative effects on chironomid emergence during the study period in each included study. The size of the squares is proportional to the weight of the studies, which is based on the number of replicates. Liber (1998) and Duchet (2015) reported separate emergence data for different chironomid species, but to avoid statistical dependence we have used data for only one species. To obtain a conservative estimate of negative effects the species showing the least negative effect was selected (results do not change significantly if other species are used). Data in the figure represents five regions (four in Europe, one in North America). Study labels refer to short reference and study name information as recorded in Additional file 6

This outcome category included eleven response variables related to reproduction and longevity (see Additional file 5). The most frequently studied response variables are emergence and mortality. Meta-analysis was only performed for chironomid emergence; data for other response variables were too scarce. The summary (weighted average across studies) maximum negative effect on chironomid emergence, as well as the summary overall effect across the entire study period, was significantly different from zero (p < 0.05). The summary effect at the end of the included studies was, however, not significant, indicating that the effects are not long-lasting. The *I*^*2*^-statistic (> 80%) indicates a high heterogeneity of study results. The summary effects, their 95% confidence intervals and p-values together with other statistics, including diagnostic statistics, are shown in Additional file 7: Table S1. Also shown in the table are the changes in chironomid emergence calculated from back-transformed summary effect sizes. A forest plot of the maximum negative effects is shown in Fig. 11. We judged the risk of bias in included studies to be low to moderate (therefore no sensitivity analysis excluding studies with higher or unclear risk of bias was conducted). The time from last Bti application to last sampling occasion in the studies ranged from 12 to 91 days. Application rate was not significant as an effect moderator (Additional file 7: Table S25), but statistical power for these analyses was generally low. An investigation using highly standardized mesocosms [11] reported larger effects for higher application rates when other conditions were identical. Funnel plots (Additional file 7: Fig. S13) do not indicate any obvious publication bias.

In addition to the studies included in the meta-analysis, Bond et al. [46] studied reproduction of chironomids in field experiments and found that inhibition of reproduction lasted 3 weeks following Bti treatment (Vectobac AS at recommended rate), whereas the overall reduction during the study period (July-December) was 19% compared to the control treatment. These effects were comparable to or larger than those observed on *Ae. aegypti* and *Culex spp*. mosquitoes, respectively [46]. Duchet et al. [47] studied *Daphnia pulex* and *D. magna* population intrinsic growth rate under both laboratory and field conditions but found no effect of Bti at recommended application rates. Hanowski et al. [48] studied Red-winged Blackbird (*Agelaius phoeniceus*) and found significant effects of Bti treatments on clutch size, hatch rate and overall reproductive success in some years but not all. However, significant differences in clutch size between treatment areas and control areas were also observed before the Bti treatments commenced, making it difficult to assess how much Bti application contributed to differences observed during the treatment period. No effect was detected on survival or fledgling success. The risk of bias in these three studies were judged to be unclear. In another study, house martins (*Delichon urbicum*) had a significantly smaller clutch size, fledgling survival, and overall breeding success at Bti treated relative to control sites, but there was no effect on hatch rate [18].

#### Abundance

**Fig. 12:**
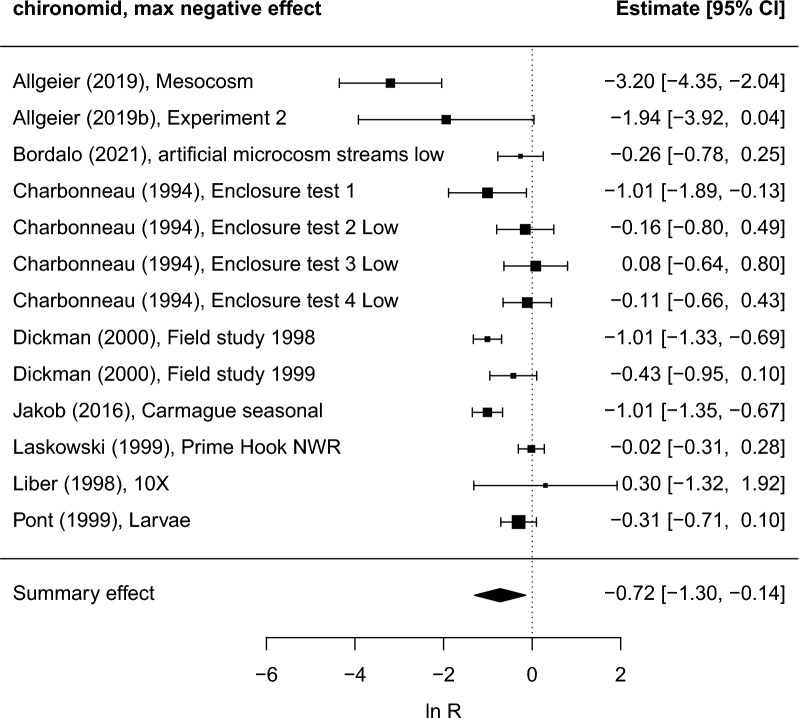
Forest plots showing maximum negative effects on Chironomidae abundance during the study period in each included study. The size of the squares is proportional to the weight of the studies, which is based on the number of replicates. Liber (1998) and Bordalo (2021) reported separate abundance data for different chironomid species, but to avoid statistical dependence we have used data for only one species. To obtain a conservative estimate of negative effects the species showing the least negative effect was selected (results do not change significantly if other species are used). Data represents eight regions (four in Europe, three in North America, one in Asia). Study labels refer to short reference and study name information as recorded in Additional file 6

All response variables in this outcome category were sufficiently comparable for potential inclusion in the same meta-analysis. However, the number of studies was sufficient to perform meta-analyses for chironomids, crustaceans, and molluscs only. For chironomids, the summary max negative and summary overall effects were significant and negative, with a high *I*^*2*^ statistic (> 80%) indicating large heterogeneity among study results (Fig. 12 and Additional file 7: Table S3). The risk of bias in included studies are judged to be low to moderate (therefore no sensitivity analysis excluding studies with higher or unclear risk of bias was conducted). Application rate was not significant as an effect moderator (Additional file 7: Table S26). Funnel plots do not indicate any obvious publication bias (Additional file 7: Fig. S14). Other studies that could not be used in the meta-analyses (effect size or variability could not be calculated) showed varying results. In some of these [49, 50] the risk of bias was judged to be “high” or “probably high” and therefore the results from them are not further considered here. In experimental field studies where the risk of bias was judged to be “unclear”, Sáringer et al. [51] and Wolfram et al. [52] reported no significant difference between treated and untreated areas. The authors of the latter study stressed the importance of using a BACI design for field studies as differences between heterogenous areas may confound differences in habitat properties with the Bti treatments. However, we found no significant summary effects when analysing data from studies using either BACI or CI designs separately (Additional file 7: Table S29).

**Fig. 13:**
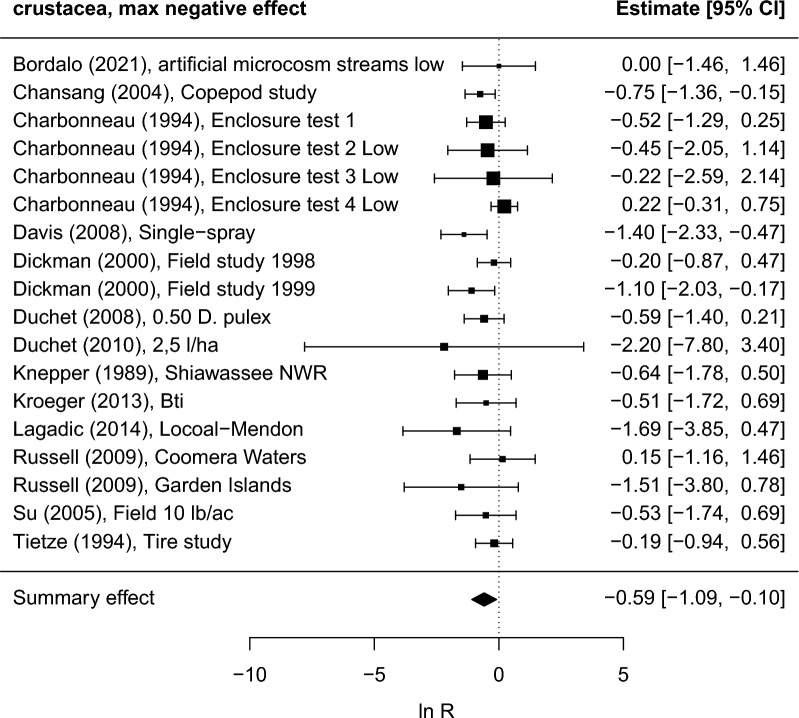
Forest plots showing maximum negative effects on Crustacea abundance during the study period in each included study. The size of the squares is proportional to the weight of the studies, which is based on the number of replicates. Kroeger (2013) reported separate abundance data for different taxa groups, but to avoid statistical dependence we have used data for only one taxa group. To obtain a conservative estimate of negative effects the taxa group showing the least negative effect (Cyclopoida) was selected (results do not change significantly if other species are used). Data represents 13 regions (five in Europe, five in North America, two in Asia, one in Australia). Study labels refer to short reference and study name information as recorded in Additional file 6

For crustaceans, the summary maximum negative effect is significant (p < 0.05), i.e., the abundance is lower in treated areas (Fig. 13). In contrast to the case of chironomids, the heterogeneity of results seems to be low as indicated by a *I*^*2*^-value of 3% (Additional file 7: Table S5). For eight of the studies the risk of bias was judged to be high, probably high, or unclear. If these studies are excluded from the meta-analysis the summary effect is no longer significant (see sensitivity analysis in Additional file 7: Table S30). Neither the summary effect across study periods nor that at the end of the study periods were significantly different from zero (Additional file 7: Table S5). Application rate was not significant as an effect moderator (Additional file 7: Table S27). Funnel plots are shown in Additional file 7, Fig. S15. Two studies on crustaceans reported in one publication [50] could not be used in the meta-analyses. The risk of bias for those was judged to be “probably high” and “high”, respectively. For molluscs no significant summary effects were found (Additional file 7: Table S7).

Among other organism groups, the abundance of Coleoptera (Hydrophilidae) and Hemiptera (Corixidae), feeding on *Ae. aegypti* in Bti treated water jars in Thailand [53] did not differ from untreated jars. Similar results for Coleoptera and Hemiptera were obtained in California [50] and Madagascar [54], and for Coleoptera (Dytiscidae) in Sweden [23, 55], but the risk of bias in the latter four studies was judged to be “high” or “probably high”. The abundance of Ephemeroptera was reported as negatively affected in a field study performed at Lake Balaton, Hungary [51]. Two other studies on Ephemeroptera [50, 54] were judged to have a “probably high” or “high” risk of bias. The impact of Bti treatment on the abundance of Odonata at Lake Balaton was low [51]. Two other studies on Odonata [50, 54] were judged to have a “probably high” or “high” risk of bias. Niemi et al. [56] studied the abundance of 19 bird species in Minnesota, USA, and detected no effect of Bti treatments. In the same area, a study with “unclear” risk of bias found no evidence to suggest that breeding bird communities or species in the studied wetlands were affected by treatments with Bti [57]. In contrast, Poulin et al. [2] found significantly lower counts of several bird species in Bti treated areas in Camargue, France.

Investigations of abundance have also been conducted for algae [58, 59], diptera [51, 55], Haemosporodia [60], Hymenoptera [51], Lepidoptera [51], Trichoptera [51], Protozoa [24], as well as plankton [56, 59], but these taxa have only been investigated in one or two studies, making it difficult to synthesise the results. These studies point to a highly variable picture in abundance effects among taxa. More studies have investigated the abundance of the taxa groups Insecta [55, 56, 61] and invertebrates [49, 62–68]. However, the often unclear and presumably varying assemblages of species included in these studies, combined with the fact that we judged most of the studies to have a “probably high” or “high” risk of bias, makes it difficult to draw any reliable conclusions based on their results.

Finally, among studies focussed on micro-organisms, Nguyen et al. [69, 70] suggested that bacterial densities were less strongly reduced when Bti was applied to outdoor mesocosms with water enriched in organic matter due to a decreased interaction with mosquito larvae, and that Bti treatment had little effect on total bacterial densities. Similarly, Xu et al. [71] reported a higher abundance of slow growing bacteria after Bti treatments in tree hole habitats, with bacterial total abundance being unchanged. Hajaij et al. [72] reported the abundance of *Bacillus* species being more dependent on the biotope than on Bti application. The latter study was however judged to have a “high” risk of bias, but the results are mainly supported by three studies in Sweden [21, 73, 74] with “unclear” risk of bias.

#### Diversity

Diversity measurements included 10 different response variables, of which Shannon’s diversity index, species/taxa richness, and Pielou's evenness index are most frequently reported. However, only species/taxa richness could be used in meta-analysis, either due to a lack of measurements of variability or an insufficient number of studies for the other indices. Looking at the time of maximum negative effect and over the entire study periods, small but statistically significant summary effects were detected for invertebrates in our meta-analysis (Additional file 7: Fig. S5), but that is not the case if only studies with low to moderate risk of bias are included (see sensitivity analysis in Additional file 7: Table S31). No significant summary end-of-study effect could be detected through meta-analysis. A relatively large number of studies (16 in eight articles) recording species/taxa richness data were excluded from meta-analysis due to inadequate reporting of data variability. Wolfram et al. [52] and Theissinger et al. [75] studied chironomid species richness but found no effect of Bti treatments. No effect was also reported by Niemi et al. [56] who studied richness of cladoceran, copepod, rotifer, and total zooplankton taxa. In contrast, Marina et al. [7] reported significantly different species accumulation curves (during 25 weeks of sampling) for aquatic insect species from experimental pools in southern Mexico. Other studies, on aerial insects [61] and non-target aquatic invertebrates [49], were judged to have a “probably high” or “high” risk of bias.

Duguma et al. [76] found a higher richness in microbial communities in experimental microcosms treated with a high dose of Bti (9.62 × 10^9^ ITU/ha) compared to a lower dose (0.12 × 10^9^ ITU/ha) and an untreated control. Tetreau et al. [77] studied the bacterial microbiota of *Ae. aegypti* larvae and found a lower species richness in larvae treated with Bti compared to untreated larvae, but only in larval strains tolerant towards Bti. In contrast, microbial richness found in Bti susceptible and intermediate susceptible strains was larger in Bti treated larvae.

Effects of Bti on Shannon’s index have been studied for invertebrates [49, 67, 78], insects [7, 61], Chironomidae [52], phytoplankton [59], and bacteria [76, 77]. For most of these studies we judged the risk of bias to be “probably high” or high”, but one study [7] with a “moderate risk of bias” showed no significant difference in Shannon’s index for aquatic insects between Bti treated and untreated ponds in Mexico. The same result was obtained for Chironomidae [52] in Austria. In contrast, microbiome Shannon diversity was reduced in mosquitoes that had developed tolerance towards Bti, suggesting the development of resistance to Bti might be associated with changes in microbiota [77]. The risk of bias in the two latter studies was judged to be “unclear”. Effects of Bti on Pielou's evenness index have been studied for phytoplankton [59], invertebrates [67, 78], insects [61], and bacteria [77]. The study results and our risk of bias assessments are similar to those for Shannon’s index.

Other measures of diversity that have been reported include phylogenetic bacteria diversity [76], dominance of insect genera [12], Simpson’s index for bacteria [77], chironomid saprobic index [75], Hurlbert’s PIE for dytiscids [23], and McQuitty’s similarity by taxonomic order, Nematocera families, and Coleoptera families [55]. Overall, although significant differences in various diversity metrics between Bti-treated areas and untreated areas were found in some cases, the disparate nature of these studies (e.g., in sampling design, organism groups, temporal scale) means we are not able to draw reliable conclusions.

#### Community composition

The most commonly reported response variables in the community composition category are Bray–Curtis dissimilarity/similarity [49, 61, 75, 76, 78, 79] and relative abundance [10, 52, 61, 76, 80]. None of the reported response variables could be used in meta-analysis. The Bray–Curtis dissimilarity reported by Duguma et al. [76] demonstrated that bacteria from high Bti treatments (9.62 × 10^9^ ITU/ha) separated significantly from low Bti (0.12 × 10^9^ ITU/ha) and control treatment samples. Theissinger et al. [75] reported a small but statistically significant dissimilarity in chironomid community composition. The Bray–Curtis dissimilarity was studied also for invertebrate and chironomid communities [49, 78],insects, [61], and salt-marsh arthropods [79], but we judged the risk of bias to be “probably high or “high” in these studies. In a mesocosm study, Allgeier et al. [10] investigated aquatic invertebrates and recorded significant lower relative abundances of Culicidae and Chironomidae. In this study, differences in community composition between Bti-treated and untreated mesocosms were also demonstrated through Principal Response Curves. Duguma et al. [76] recorded a lower relative abundance of cyanobacteria in mesocosms treated with high doses compared to low Bti and control mesocosms. Receveur et al. [80] studied the microbiome of larval mosquitoes in temporary, hurricane created, habitats. They found that Bti-treatment was associated with differences in relative abundances of key bacterial groups but attributed this to an indirect effect arising from delayed larval development, rather than a direct impact of Bti on microbial community structure. Receveur et al. [80] also observed lower relative abundances of photosynthetic algae in mosquitoes subjected to Bti treatment, and which they could not explain as an indirect treatment effect. Xu et al. [71] studied tree hole bacteria and found that removal of mosquito larvae through Bti treatment altered bacterial phylogenetic distribution. Wolfram et al. [52] studied chironomid communities but found no Bti related differences in their composition. Lundström et al. [81] used Jaccard’s index to calculate the annual species turn-over rate in wetlands and reported higher rates in Bti treated wetlands compared to untreated wetlands. Generally, we judged the risk of bias to be lower in the studies on relative abundance than in the studies on diversity and dissimilarity response variables, but it is still difficult to draw any general conclusions based on existing studies. However, it seems that some changes in community compositions may be related to Bti application.

#### Species traits/feeding groups

In terms of number of response variables, the category of species traits is the most diverse, including both physical and behavioural variables. Physical endpoints are mostly related to weight or size of an organism or body parts, whereas behavioural endpoints are related mainly to feeding. We have performed meta-analyses for size, weight, and predation (feeding rate). For size, no summary effect was significant (Additional file 7: Table S11). Various species of Amphibia, Crustacea, and Odonata were included in the meta-analysis. Subgroup analysis show no significant effect for any individual taxa group (Additional file 7: Fig. S6). The number of studies in each group is, however, small. The risk of bias in included studies was most often “probably low”. For weight, fewer studies have been conducted and no significant summary effect could be detected by our meta-analysis (Additional file 7: Table S13). This is also true for feeding rate (Additional file 7: Table S15), but in the latter case there are some additional studies that could not be used in meta-analysis due to a lack of reporting of data variability. Olejnicek and Maryskova [82] studied common backswimmers (*Notonecta glauca*) and noted that these did not attack mosquito larvae killed by Bti. In contrast, Rebollar-Tellez et al. [83] found that the predatory capacity of *Buenoa sp*. was greater when their prey, *Culex pipiens* larvae, was treated with Bti. Roberts [84] studied crustaceans (*Gammarus duebeni* and *Palaemonetes varians*) and found that they preferred live mosquito larvae over Bti-killed larvae, although Bti-treatment did not influence the Crustacea feeding rate. Gunasekaran et al. [85] found no effect of Bti treatment on the feeding rate of *Notonecta sp.* and *Diplonychus indicus*.

Allgeier et al. [62] conducted a mesocosm study to investigate how potential effects of Bti delivered in a granulate form (2.88 × 10^9^ ITU/ha in each mesocosm) on non-target chironomid larvae affect the development time of two predatory newt species (*Lissotriton helveticus, Lissotriton vulgaris*), and found no significant difference between Bti-treated and untreated replicates. They also studied the Body Condition Index (BCI) of the newts and a dragon fly species (*Aeshna cyanea*). While there was no significant effect on the newts, the BCI of the dragonflies was significantly lower in treated areas compared to untreated areas (p < 0.001). Hanowski et al. [48] studied the behaviour of red-winged blackbirds (*A. phoeniceus*) and found no significant effects on visits to nest (female and total) or on male fledge age. Kimball and Williams [86] investigated the effects of Bti on rice grain yield, crop height, and crop maturity (as measured by harvest moisture content) in two studies but found no significant effects. Su and Mulla [87] studied the fraction of gravid tadpole shrimps (*Triops longicaudatus* or *Triops newberryi*) at two different Bti application rates but found no significant difference relative to controls.

#### Food web structure/biomarkers

Seven articles reported in total 13 response variables related to food sources. None of these could be synthesized in meta-analysis. Allgeier et al. [62] studied carbon and nitrogen isotopic compositions and niche widths of newts (*L. helveticus, L. vulgaris*), dragonfly (*A. cyanea*), and their prey. Although Bti treatment reduced chironomid abundance, they were preferred over other invertebrates and comprised the major component in the newts' diet (ca. 55%) regardless of their availability. However, the presence of dragonflies decreased survival of newt larvae in Bti treated mesocosms, possibly due to dragonfly nymphs preying on newt larvae when chironomids were less available. Wolfram et al. [52] found no effect of Bti treatments on relative proportions of chironomid feeding guilds, but highlighted the need for investigations to be conducted over longer periods, to reveal possible delayed effects. Poulin et al. [18, 88] examined prey taxa and prey size in house martin faecal samples and found that at Bti treated sites, the diet was enriched by ants and depleted in Diptera, Odonata, and Araneae compared to control areas. In treated areas it was also enriched in small but depleted in large prey. A food availability index [89], which is positively correlated with number of reed passerines [90], has been shown to be significantly lower in Bti treated areas compared to untreated control areas [88, 91].

#### Changed ecosystem processes

In this outcome category only one study was found. In laboratory mesocosms, Bordalo et al. [63] studied leaf litter decomposition rates at three different Bti concentrations (12, 120, and 1200 µg/l) and found significantly lower decomposition rates at all concentrations, compared to untreated control mesocosms.

#### Environmental data

In this outcome category 16 response variables were reported. The most studied environmental response variables were pH, dissolved oxygen (DO), suspended particles, and chlorophyll a. Quantitative synthesis through meta-analysis of a rather small number of available studies showed no significant summary effect on pH, DO, or chlorophyll a (Additional file 7: Tables S17, S19, S21). However, in one study by Duguma et al. [76], a significant negative effect was observed for all these response variables in the afternoon (when photosynthesis by the phytoplankton was likely at its peak) at a high Bti application rate (9.62 × 10^9^ ITU/ha). A reduction in DO was also observed by Su and Mulla [58]. One additional study on pH and DO [92], not used in the meta-analyses, was judged to have a “probably high” risk of bias. Another study not used in meta-analysis showed no significant effect of three different Bti application rates on chlorophyll a concentration [63]. For suspended particles, meta-analysis resulted in a significant summary maximum negative effect (Additional file 7: Table S23). Again, the largest effects in a single study were observed by Duguma et al. [76] at a high Bti application rate. It was, however, unclear to the authors whether the decrease in chlorophyll a concentration and abundance of sestonic particles in the water column were directly related to toxins or degradation products of Bti, proprietary components of the VectoBac G formulation or recycling of Bti in mosquito carcasses.

Other response variables reported were chemical oxygen demand (COD), sulphate, total phosphorus (TP), total nitrogen (TN) [76], and estradiol equivalents [93]. While a low application rate of Vectobac G resulted in no effect on COD, sulfate, TN, or TP, a high application rate (two times the recommended dose) resulted in decreased concentrations of all these response variables, possibly related to a reduced phytoplankton biomass [76]. Maletz et al. [93] showed a link between VectoBac® TP and estrogenic activity although the source of estrogenic activity remained unclear. However, water samples taken from field experiments with VectoBac TP and VectoBac WDG did not show estrogenic activity with the potential to cause adverse effects in the aquatic ecosystems.

#### Persistence in the environment

When studying persistence of Bti in the environment, the most common response variables are spore and colony concentration. Khawaled et al. [35, 94] studied recycling of Bti in mosquito pupae on relatively short timescales (up to 290 h) and found that the spore concentration reached a maximum after about 60–100 h followed by a decrease. However, our main interest was in long-term persistence, and in particular whether Bti recycling occurs under field conditions. Studies have shown that although the abundance of Bti spores and colonies usually decrease with time, residues can persist for months on vegetation [72, 95, 96], leaf litter [97], pebbles [95], periphyton [96], in soil and sludge [98–101], sediment [95, 96], water [95, 96, 99, 102], and waste tires [103]. It has also been shown that Cry toxin – the toxic component of Bti—concentrations and toxicity may persist for similar periods [84, 96, 104]. Repeated Bti applications could cause accumulation of Bti over time. However, one long-term study on a Swiss natural wetland where repeated Bti applications had been conducted for 22 years showed no evidence of such accumulation [100]. The other concern is whether Bti from commercial mosquito control agents has the ability to reproduce in the environment if not sterilised. Tetreau et al. [104] found indications of recycling of Bti spores in the Rhône-Alpes region, France, as they recorded higher than expected concentrations of Cry4 toxins in leaf litter after Bti treatment. However, a subsequent study in the same area was not able to show any evidence of recycling [101]. Also in the Rhône-Alpes region, Tilquin et al. [97] found an unexpected high number of Bti spores on leaf litter and could not exclude the possibility that the bacteria germinate and proliferate in situ. Overall, results from these studies are regarded as suggestive rather than proving recycling of Bti spores from commercial mosquito control agents.

## Review limitations

The scope of this systematic review is very broad and encompasses more than 100 response variables, some of which were reported in only one or a few publications. Even though some of these are closely related and could be combined in the same meta-analyses, the number of outcomes is still larger than would normally be considered in systematic reviews. From a strict systematic review point of view this is not ideal, as it is unrealistic to discuss and compare such a large range of different outcomes in depth, and undesirable to conduct formal statistical analyses for variables represented by so few studies. On the other hand, the Swedish stakeholders were very clear about the importance of not focusing on a single detail such as abundance of some specific taxa. To make the systematic review useful for decision-making, it is necessary to convey a more complete picture of ecosystem effects. Consequently, this review is a compromise in combining in depth meta-analyses of selected variables with a summary overview of a broader range of outcomes. However, even if we had the resources to conduct all in-depth analyses that theoretically would be possible (meta-regressions etc.), this systematic review remains limited by a rather small number of studies per outcome. Another limitation in the conduct of this systematic review is that we have not been able to consider studies published in every language. During title and abstract screening, we included 32 articles in various languages that seemed to be highly relevant, but lacked time and translation recourses to use the results. These articles are listed in Additional file 4.

In our critical assessment of the publications included in our systematic review, a large proportion of studies were judged as carrying either a “probably high” to “high” or “unclear” risk of bias, and many also had inadequate or no reporting of variation among mean responses. This highlights the widespread use of inadequate statistical designs and/or methodologies, or else under-reporting of important methodological details, in a large portion of published research on Bti impacts to date. Whilst we were able to use information on bias risk in sensitivity analysis as part of our metanalyses, it was harder to account for biases when writing our narrative syntheses, beyond flagging the presence of confounding factors or limitations in study design in some cases.

While many Bti treatment programmes run for several years with multiple Bti treatments each year, most experimental field studies are limited to shorter time periods and fewer Bti treatments. It is therefore not clear how applicable the results of these studies are for larger scale treatment programmes with repeat applications. Finally, we also discovered variables in our meta-analyses where most recorded observations were associated with particular geographic regions. For example, all studies showing significant negative effects on chironomid emergence were conducted in Germany by the same research group. These studies were conducted with a high degree of rigor, but their limited geographic scope necessitates a degree of caution when generalising the observed impacts across a broader range of habitat types and geographic habitats. The limited geographical range of available data for chironomid emergence highlights the need for research of similar quality assessing Bti effects on chironomid emergence elsewhere in the World and in other ecosystem types. In contrast, the overall negative effect size of Bti treatment on chironomid abundance was driven by results from more than one research group, and from more than one country, allowing a higher degree of generalisability for the effect of Bti on chironomid abundance.

## Review conclusions

### Implications for policy/management

Our metanalyses revealed a consistent overall negative effect of Bti treatment on the abundances of Chironomidae and Crustacea, based on a relatively small number of studies but from several different countries or states. However, the result for Crustacea was partly based on evidence for studies assessed as having a high risk of bias, and when these were excluded the effect was no longer significant. In contrast, the evidence for impacts on chironomid abundance was based entirely on studies assessed as having low-moderate risk of bias. Multiple additional effects of Bti were also detected by our metanalyses as well as in individual publications, albeit often associated with single or only a few studies. The negative effects of Bti treatment on Chironomidae, which reflect their close phylogenetic relationships with mosquitoes, have potential significance ecologically. The Chironomidae are represented in most freshwater food webs with a high ecological diversity (encompassing predators, algal grazers, suspension and deposit feeders, as well as detritivorous species) and are also typically among the most productive freshwater taxa [105, 106]. Furthermore, Chironomidae typically constitute a highly abundant prey item for not only aquatic predators, but also terrestrial predators once they have emerged from the aquatic habitat as winged adults [107, 108]. Accordingly, impacts on chironomids as NTO have particularly strong potential to affect food web integrity, and both terrestrial and aquatic organisms that depend on chironomids as a food source [18, 109–111]. More research is required to quantify these possible indirect consequences of changed chironomid abundance following Bti treatment.

We thus emphasise that, on the one hand, there is now a large number of studies highlighting direct and indirect effects on NTOs and ecosystems of mosquito control using Bti. Many have been conducted directly in the field and assessed operational Bti application rates as used in mosquito control. On the other hand, the paucity of rigorous studies conducted with low risk of bias for most response variables undermines our capacity for evaluating how common most of the effects documented here might be. Often, negative effects observed in one study, habitat type and/or geographic region have not been observed in similar studies conducted in different habitat types elsewhere. In many cases, it is likely that these differences are attributable to differences in study rigor, especially differences in the level of replication and consequent statistical power. However, in some cases, differences in study outcomes might indicate that the occurrence and strength of Bti effects on NTOs and other ecosystem properties are sometimes context dependent, varying with aspects such as differences among treated ecosystems in environmental characteristics and species pools, or differences in more technical aspects such as realised dosage levels or the timing of monitoring. Presently we lack the data and knowledge base to evaluate the occurrence of context-dependency in Bti effects, or whether inconsistent observations are primarily attributable to differences in aspects such as study design and performance.

An evaluation of the environmental and ecological factors associated with increased vulnerability to Bti treatments is critical for identifying what types of ecosystems are most at risk from NTO and indirect effects of Bti control. However, until the knowledge base develops sufficiently for such a vulnerability analysis to be undertaken, we suggest the potential for negative, indirect effects of Bti treatment in ecosystems should not be discounted a priori*,* and needs to be balanced against positive outcomes of mosquito control for *inter alia* human health, well-being, and activities.

More extensive monitoring and assessment of Bti effects will help provide the data needed to reduce uncertainties regarding NTO and other, indirect impacts on ecosystems. This includes monitoring of not only the effects of mosquito control for TO and the most sensitive NTOs (clearly Chironomidae in our study), but also of unforeseen outcomes arising from reductions in TO and sensitive NTO abundances for other organisms (including endangered species) and ecosystem properties (e.g., ecosystem functioning and services).

Finally, our syntheses highlighted several studies demonstrating the potential for persistence of Bti in the environment. This indicates that using sterilised forms or monitoring Bti persistence may be justified, to reduce risks of recycling of the bacterium in the environment and potential for collateral effects, especially outside the period of Bti treatments.

Based on our data synthesis and discussion above, we draw the following conclusions regarding the secondary questions posed in this systematic review:

1. While many mosquito control programmes run for several years with multiple Bti treatments each year, most experimental field studies are limited to shorter time periods and fewer Bti treatments. It is therefore not clear how applicable the results of these studies are for full scale control programmes, and based on available data we are not able to determine whether effect sizes or the number of indirect effects [15] increase in ecosystems subjected to long-term and repeated Bti treatments.
2. When we synthesise the study results in meta-regressions, we do not see any clear dose–response relationship. However, some articles investigating more than one dose have reported stronger effects for higher doses where all other conditions were identical. In many cases the higher doses used were significantly higher than recommended for mosquito control.
3. The small number of eligible studies per outcome makes it difficult to statistically identify ecosystem properties that may regulate the occurrence and magnitude of Bti effects on NTOs and other ecosystem properties.
4. The only response variables for which non-intended effects have been shown to last for months after treatment are chironomid abundance and concentration of Bti spores in the environment. It is however not clear if these effects can last from one year to another if treatments are ceased, or accumulate if treatments are continued.

### Implications for research

The knowledge base for assessing indirect effects of Bti treatment programs on ecosystems is diverse, with a wide variety of response variables assessed. However, published studies are also very disparate, characterised by wide variation in study design and the rigorousness of method and result reporting. As such, it is often very difficult to distinguish whether variation in the direct and indirect effects of Bti are attributable to particular environmental characteristics of the ecosystem, or due to variation in study design, methodology and performance. Distinguishing these two sources of variation in study outcomes is essential, since understanding the factors driving variation in the indirect effects of Bti treatments would greatly enhance our capacity for formulating concrete recommendations regarding where and when Bti is most likely to have additional, negative and indirect ecological effects.

A greater focus is thus needed on understanding the factors which increase the risk of indirect ecological impacts of Bti, and which factors limit negative indirect impacts. This requires increased (i) reporting and analyses of both abiotic environmental (temperature, wind, soil and water chemistry) and biotic (species pool, vegetation cover) factors that could potentially affect the indirect impacts of Bti treatments in all studies, and (ii) development of studies explicitly investigating how abiotic and environmental variation affects treatment outcomes. Studies should also a priori identify the ecosystem attributes most likely to be affected by Bti treatment, based on ecological knowledge of the biota and environment, so that studies quantify the most relevant endpoints.

Accordingly, future research should aim to employ a more rigorous and well-replicated approach to studying Bti impacts in semi-field mesocosms or in the field, ideally following a BACI design, combined with a greater rigor in reporting key methodological details essential for assessing study rigor (*inter alia* Bti dosage, statistical design, possible confounding factors and how they were addressed), and directly addressing effects of specific environmental factors hypothesised a priori to regulate outcomes of Bti treatments. Recent examples of research meeting at least some of these criteria include mesocosm experiments assessing aquatic macroinvertebrate community changes and odonatan emergence [112], chironomid emergence dynamics [111] frogs [113] and indirect effects on riparian spiders [110], along with a field study replicated at the wetland scale which used biomarkers to track changes in the structure of floodplain food webs [15].

Finally, in some regions there are long term data sets available on the effects of Bti mosquito control on both TOs and NTOs. Rigorous analysis and environmental assessment of such data by independent researchers is likely to be of great value in not only assessing the effects of Bti control locally, but in generating hypotheses for future research.

## Supplementary Information

**Additional file 1. **Stakeholder engagement.

**Additional file 2.** Literature searches.

**Additional file 3.** Extraction forms.

**Additional file 4.** Not retrieved and excluded articles at full text screening.

**Additional file 5.** Reported response variables.

**Additional file 6. **Narrative synthesis tables and extracted data.

**Additional file 7.** Meta-analysis results.

**Additional file 8. **ROSES for Systematic Review Reports.

## Data Availability

All data generated or analysed during this study are included in this published article and its supplementary information files.
