# Supplementary material for: Effects of mosquito control using the microbial agent Bacillus thuringiensis israelensis (Bti) on aquatic and terrestrial ecosystems: a systematic review
Source: Environ Evid. 2023 Nov 22;12:26. doi: 10.1186/s13750-023-00319-w (PMC11378846; doi:10.1186/s13750-023-00319-w)
Supplement: Supplementary file 1 — Additional file 1. Stakeholder engagement. [file 13750_2023_319_MOESM1_ESM.pdf]

Stakeholder engagement

Identification of stakeholders

The main stakeholder of this systematic review is the Swedish Environmental Protection Agency (SEPA), with which Formas started to discuss the possibility of conducting a systematic review in 2018. Another obvious stakeholder is the County administrative board of Gävleborg, which during 2010-2013 conducted an assignment from the Swedish government, investigating long-term solutions to the mosquito problem in the lower River Dalälven area in Sweden. Identification of other stakeholders started by looking for organisations that in some way had been involved in previous permitting processes. Some stakeholders were identified through discussions with stakeholders we had already identified (to some extent the stakeholder identification was thus like a snow-balling process).

Stakeholder meeting

A Stakeholder meeting took place on 24 October 2018 at Gävle slott (Castle of Gävle) in Gävle, Sweden. The meeting was introduced by the governor of the County administrative board of Gävleborg. Fifteen participants from 10 organisations attended the meeting. The review team was represented by Magnus Land and Brendan McKie. Two people from Biological Mosquito Control were unable to participate but attended a separate meeting with Magnus Land on 26 November 2018.

Invitation and participation

An invitation to the stakeholder meeting was sent to identified stakeholders and published on Formas' website. The meeting was open to anyone who was interested. The invited organisations are shown in Table 1.

Agenda

- Welcome – Short introduction by the governor of the County administrative board of Gävleborg *Per Bill.*
- Presentation of Formas and Formas' assignment to conduct evidence syntheses.
- Introduction to systematic review methods and why a systematic review on mosquito control using Bti was initiated.
- Presentation of the review team and a short introduction to the scientific basis for assessing ecosystem effects.
- Discussions.

The discussions, which constituted the main part of the meeting, centered around seven questions, some of which were discussed in plenum while others were discussed in smaller groups. The discussions were documented using the web-based tool Howspace (https://howspace.com/).

The questions discussed were:

1. Where to search for literature? Which organisations to contact? (small groups)
2. How should ecosystem be defined? (plenary)

3. Which indicators are useful for probing effects on ecosystems? (plenary)
4. Which factors can cause heterogeneity across studies? (small groups)
5. What should the inclusion/exclusion criteria for studies be? (small groups)
6. How should study validity be assessed? What makes a study reliable or unreliable? (small groups)
7. How should the results be presented? How can we make the review useful? (plenary)

Outcomes of the meeting

Here we summarise key comments from the discussions at the stakeholder meeting. A general comment was that some of the questions were quite difficult to discuss as many participants did not have a scientific background. In many cases conflicting comments were made by different participants.

Review question

- Clarify whether the review is about effects of Bti treatments or effects of decreased mosquito abundance.
- It is most interesting to isolate the effects of Bti treatment and compare with effects of other methods for mosquito control.

How should ecosystems be defined?

- You can't separate aquatic and terrestrial ecosystems –they are connected.
- Ecosystems are about food webs. Interesting to see how far away (trophic levels) from the target species you can find effects.

Which indicators are useful for probing effects on ecosystems?

- Important to separate different response variables and not mix them unless justified
- Eutrophication may be a useful indicator

Which factors can cause heterogeneity across studies?

- Type of landscape – rich/diverse or poor (if you include studies from other countries)
- Species composition before Bti treatment
- Number of treatments/year, Dose/treatment
- Flow regime, water level
- Timing of treatment (seasonal)
- Treatment history (longevity and intensity)

Which studies are relevant? What should the inclusion or exclusion criteria be?

- Do not include studies from the tropics
- Include studies from all areas, also those from the tropics
- Focus on temperate regions
- Include only studies where target species is relevant (flood mosquitos)
- Include studies regardless of mosquito target species
- Focus on treatments using VectoBac G
- Do not include studies investigating direct effects of Bti on non-target species

What makes a study reliable or unreliable?

- Long-term studies are good
- Studies on large scales (landscape) are preferred
- Laboratory studies are less valid
- Studies performed by people who have a vested interest in mosquito control are less reliable

Based on the discussions, four questions in addition to the main systematic review question were found to be of general interest to answer:

1. Are ecosystems more affected by long-term and repeated Bti treatments compared to short-term or single treatments?
2. Is there a clear dose-response relationship?
3. Do landscape characteristics (e.g., in terms of floodplain characteristics, vegetation type, species composition etc) affect the type or size of effects?
4. Are observed effects transient or long-lasting after treatment?

Table 1. Organisations invited to the stakeholder meeting on 24 October 2018, arranged by Formas in collaboration with the County Administrative Board of Gävleborg, Sweden. Participating organisations in bold.

Organisation (in Swedish)	Organisation (in English)
**Biologisk Myggkontroll[1)]**	**Biological Mosquito Control[1)]**
Sveriges ornitologiska förening	Birdlife Sweden
**Länsstyrelsen i Dalarna**	**County administrative board of Dalarna**
**Länsstyrelsen i Gävleborg**	**County administrative board of Gävleborg**
Länsstyrelsen i Uppsala	County administrative board of Uppsala
Länsstyrelsen i Västmanland	County administrative board of Västmanland
Avesta kommun	Municipality of Avesta
Forshaga kommun	Municipality of Forshaga
Gävle kommun	Municipality of Gävle
**Heby kommun**	**Municipality of Heby**
Hedemora kommun	Municipality of Hedemora
Sala kommun	Municipality of Sala
**Sandvikens kommun**	**Municipality of Sandviken**
Tierps kommun	Municipality of Tierp
Älvkarleby kommun	Municipality of Älvkarlreby
**Nedre Dalälvssamarbetet, NeDa**	**Nedre Dalälvscollaboration/Nedre Dalälvens Development Inc.**
Region Dalarna	Region Dalarna
Region Gävleborg	Region Gävleborg
Region Uppsala	Region Uppsala
Region Västmanland	Region Västmanland
Centrum för biologisk mångfald	SLU Swedish Biodiversity Centre
Artdatabanken	SLU Swedish Species Information Centre
**Västra Gästriklands samhällsbyggnadsförvaltning[2)]**	**Spatial planning office of western Gästrikland[2)]**
Havs- och vattenmyndigheten (HaV)	Swedish Agency for Marine and Water Management
Kemikalieinspektionen	Swedish Chemicals Agency
**Naturvårdsverket**	**Swedish Environmental Protection Agency**
Skogsstyrelsen	Swedish Forest Agency
**Föreningen för myggbekämpning**	**The Association for Mosquito Control1)**
**Lantbrukarnas Riksförbund (LRF)**	**The Federation of Swedish Farmers**
Statens Veterinärmedicinska Anstalt	The National Veterinary Institute
Sportfiskarna	The Swedish Anglers Association
Naturskyddsföreningen	The Swedish Society for Nature Conservation
**Världsnaturfonden WWF**	**WWF**

[1)]Unable to attend on 24 October but participated in a separate meeting with Formas on 26 November 2018

[2)]Authors' translation (not an official English name)

Systematic review protocol

After the stakeholder meeting the review team drafted a systematic review protocol which the invited stakeholders were offered to comment on. The review team received small number of minor editorial comments. After revision of the draft a manuscript was submitted to Environmental Evidence on 24 April 2019. From that point the stakeholders have been informed on the progress of the review, but they have not been involved in any other way.
