# Supplementary material for: Effects of mosquito control using the microbial agent Bacillus thuringiensis israelensis (Bti) on aquatic and terrestrial ecosystems: a systematic review
Source: Environ Evid. 2023 Nov 22;12:26. doi: 10.1186/s13750-023-00319-w (PMC11378846; doi:10.1186/s13750-023-00319-w)
Supplement: Supplementary file 2 — Additional file 2. Literature searches. [file 13750_2023_319_MOESM2_ESM.pdf]

Bibliographic databases and search engines

Table 1. Bibliographic databases used for searching.

Database/platform	Searched field	Publisher and URL
Web of Science[1)]	topic	Clarivate Analytics, https://clarivate.com/products/web-of-science/
Scopus	title, abstract and keywords	Elsevier, https://www.scopus.com/
ProQuest Natural Science Collection[2)]	Abstract	Proquest, https://www.proquest.com/
CAB Abstracts	abstract, title, original title, broad terms, heading words, identifiers, cabicodes	Ovid, http://www.ovid.com/site/catalog/databases/31.jsp
Academic search premier	abstract or author-supplied abstract	EBSCO, https://www.ebsco.com/products/research-databases/academic-search-premier
Directory of Open Access Journals[3)]	all fields	Independent, https://doaj.org/

[1)] Including Web of Science™ Core Collection, KCI-Korean Journal Database, MEDLINE, Russian Science Citation Index, and SciELO Citation Index.

[2)] Including AGRICOLA, Agricultural Science database, Environmental Science database, Environmental Science index, Biological Science database, Biological Science index, Earth, atmosphere & Aquatic Science database.

[3)] Wildcards are not allowed. The search will be performed using the following Application Programming Interface (API) request: https://doaj.org/api/v1/search/articles/(nematocera OR midge OR diptera OR mosquito OR vector OR larv OR "black fly" OR "black flies" OR biting OR chironom OR culicidae OR simuliidae) AND (bti OR israelensis OR vectobac OR Introban OR biorational OR biopesticide OR biolarvicide).

Search results

Table 2. Search results in bibliographic databases and search engines.

Database/search engine	Date search 1	Records search 1	Date search 2	Time span search 2	Records search 2
Web of Science	2019-08-09	3968	2022-04-25	2019-2022	829
Scopus	2019-08-14	2956	2022-04-25	2019-2022	682
ProQuest Natural Science Collection	2019-08-15	3972	2022-04-25	2019-	689
CAB Abstracts	2019-08-15	3446	2022-05-05	2019-Current	545
Academic Search Premier	2019-08-15	744	2022-05-05	2019-2022	286
Directory of Open Access Journals	2019-08-15	266			
Google + Google Scholar	2019-06-25	1551			

Specialist web sites manually searched for literature.

Below is a list of specialist web sites we manually searched for literature. No relevant and accessible report beyond those picked up by Google, Google scholar, or the bibliographic databases was found.

Europe

- European Mosquito Control Association, http://www.emca-online.eu/
- KABS e.V. (Kommunale Aktionsgemeinschaft zur Bekämpfung der Schnakenplage), https://www.kabsev.de/
- Verein Biologische Gelsenregulierung entlang Thaya und March, http://mta-gelsen.at/
- Biologisk myggkontroll, http://mygg.se/
- EID Méditerranée, http://www.eid-med.org/
- EID Atlantique, https://www.eidatlantique.eu/
- EID Rhône-Alpes, https://www.eid-rhonealpes.com/
- Brigade Verte, https://www.brigade-verte.fr/demoustication
- The Netherlands Institute of Ecology, https://nioo.knaw.nl/en
- Innovative Vector Control Consortium, http://www.ivcc.com/
- Swedish Environmental Protection Agency, https://www.naturvardsverket.se/
- Federal Agency for Nature Conservation (BfN) https://www.bfn.de/en.html

North America

- American Mosquito Control Association, https://www.mosquito.org/
- Northwest Mosquito and Vector Control Association, https://nwmvca.org/
- North Central Mosquito Control Association, http://north-central-mosquito.org/WPSite/
- Northeastern Mosquito Control Association, http://www.nmca.org/
- West Central Mosquito & Vector Control Association, https://www.westcentralmosquitoandvector.org/
- Mid-Atlantic Mosquito Control Association, https://www.mamca.org/
- Mosquito and Vector Control Association, of California https://www.mvcac.org/
- Florida Mosquito Control Association, https://www.floridamosquito.org/
- Canadian Entomological Society, https://esc-sec.ca
- United States Environmental Protection Agency, https://www.epa.gov/
- Environment and Climate Change Canada, https://www.canada.ca/en/environment-climate-change.html

Rest of the world

- Asian society for Vector Ecology and Mosquito control, https://www.asiansvemc.org/
- The Society for Vector Ecology (SOVE) Indian Region, http://www.soveindia.org/
- Pan-African Mosquito Control Association, https://www.pamca.org/
- Fiocruz, https://portal.fiocruz.br/
- Mosquito Control Association of Australia, https://mcaa.org.au/
