# Supplementary material for: Effects of mosquito control using the microbial agent Bacillus thuringiensis israelensis (Bti) on aquatic and terrestrial ecosystems: a systematic review
Source: Environ Evid. 2023 Nov 22;12:26. doi: 10.1186/s13750-023-00319-w (PMC11378846; doi:10.1186/s13750-023-00319-w)
Supplement: Supplementary file 5 — Additional file 5. Reported response variables. [file 13750_2023_319_MOESM5_ESM.pdf]

Response variables recorded in nine *a priori* defined outcome categories

life history	abundance	diversity	community composition	species traits/feeding groups	food web structure/biomarkers	changed ecosystem processes	environmental data	persistence/fate
breeding success	biomass	Chao1 index	Bray-Curtis dissimilarity	adult female head width	acetylcholinesterase	leaf decomp-osition rate	chlorophyll a	colony concentration
clutch size	colony concentration	dominance	Bray-Curtis similarity	adult female hind wing length	C isotopic composition		COD	Cry toxin concentration
cumulative emergence	concentration	evenness	nestedness-resultant fraction of the Sørensen dissimilarity	adult male head width	C isotopic niche width		conductivity	faecal particle toxicity
density	density	Hurlbert’s PIE	phylogenetic distribution	adult male hind wing length	feeding guild score		DO	fraction of sample
emergence	GLMM	McQuitty similarity	principal response curve	body condition index	food availability index		estradiol equivalents	residual larvicidal activity
fledgeling success	monthly count	phylogenetic diversity	relative abundance	body length	functional groups		optical density	residual toxic activity
hatch rate	number	Pielou's evenness index	Simpson dissimilarity	body size	N isotopic composition		particles	spore concentration
mortality	number of individuals	saprobic index	species turn-over	feeding rate	N isotopic niche width		pH	spore count
net bird	number of live larvae	Shannon index	Sørensen dissimilarity	female visits to nest	prey items per faecal sample		salinity	
population growth rate	occurrence	Simpson index	unclear	grain yield	prey size		SS	
reduction in reproduction	occurrence rate	species richness		gravids	prey taxa in faecal sample		sulfate	
survival	spore concentration	taxonomic richness		harvest moisture content	Savage's index		suspended matter	
survival to metamorphosis				head width	α-carboxylesterase		temp	
weeks of no reproduction				head width growth ratio	β-carboxylesterase		TN	
				hind femur length			TP	
				hind wing length growth ratio			turbidity	
				length				
				length increase				
				male fledge age				
				male weight				
				mass at metamorphosis				
				plant height				
				predation				
				predation handling time				
				predation searching capacity				
				proportion deformed				
				size structure				
				snout-vent length				
				time to metamorphosis				
				total visits to nest				
				weight				
				young males				
