# Supplementary material for: Effects of mosquito control using the microbial agent Bacillus thuringiensis israelensis (Bti) on aquatic and terrestrial ecosystems: a systematic review
Source: Environ Evid. 2023 Nov 22;12:26. doi: 10.1186/s13750-023-00319-w (PMC11378846; doi:10.1186/s13750-023-00319-w)
Supplement: Supplementary file 7 — Additional file 7. Meta-analysis results. [file 13750_2023_319_MOESM7_ESM.pdf]

Life history, chironomid emergence

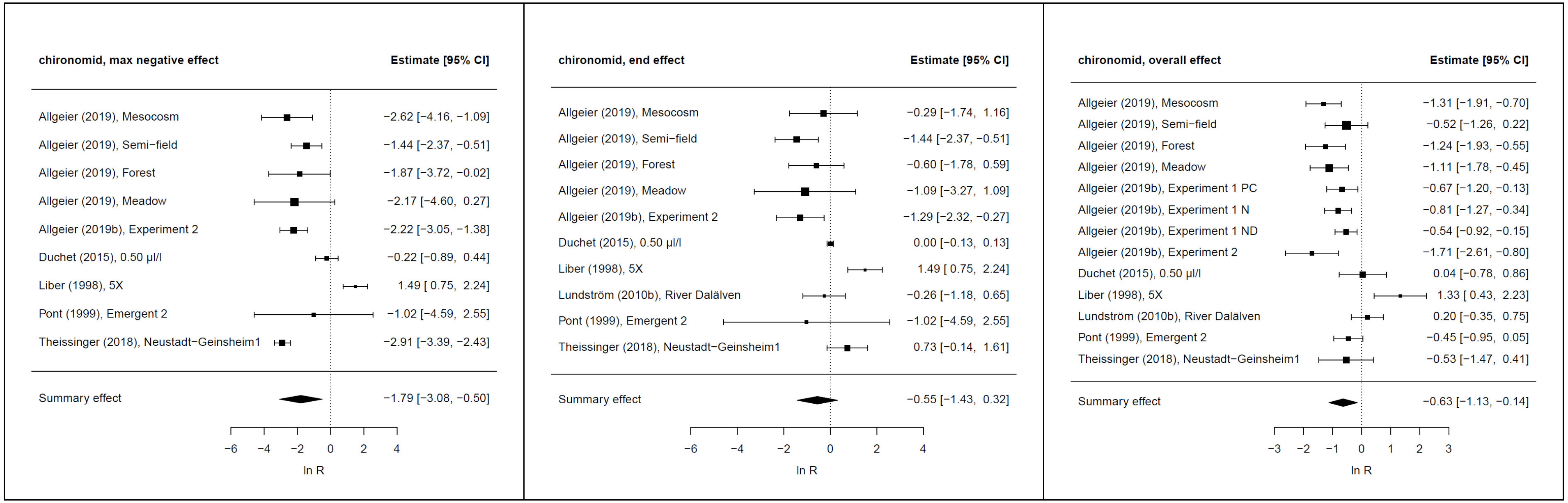

Figure S1. Forest plots showing effects on chironomid emergence in Bti treated areas compared to untreated areas. Summary max negative effect and summary overall effect is significant (p<0.05). Duchet (2015) and Liber (1998) reported data for multiple species in the same study (see **Table S2**). In those cases, we used only one of the species to avoid statistical dependence, and to obtain a conservative estimate of negative effects we selected the species showing the least negative effect (results do not change significantly if other species are used).

Table S1. Estimated summary effects (weighted averages across n studies), their 95% confidence intervals and p-values for chironomid emergence. Changes in chironomid emergence in Bti-treated areas are calculated from back-transformed effect sizes and 95% confidence intervals. The I^2-statistic reflects the heterogeneity of study results beyond random sampling error. Fail-safe number according to Rosenberg (2005).

Group	Summary effect	Estimate [95% CI]	p-value	n	Change (%)	I^2 (%)	Fail-safe N
chironomid	Max negative	-1.79 [-3.08, -0.501]	**0.0126**	9	-83 [-95 – -39]	88	220
chironomid	End of study	-0.554 [-1.43, 0.318]	0.185	10	-43 [-76 – 37]	79	--
chironomid	Overall	-0.634 [-1.13, -0.142]	**0.0158**	13	-47 [-68 – -13]	82	155
chironomid	Max positive	0.882 [-40.2, 41.9]	0.962	9	140 [-100 – 1.6e+20]	71	--

Table S2. Studies included in meta-analyses of Chironomid emergence. Bti dose is given in 10^9 ITU/ha. Number of Bti applications per year or study is denoted n_T, number of years with reported measurements is denoted n_Y, and number of reported timepoints after first application is denoted n_{tp}.

Short ref	Type of habitat	Country/State	Taxa	Response variable	Bti dose	n_T	Type of study	Exp. design	n_Y	n_{tp}	Type of datapoints	Risk of bias
Allgeier (2019)	Constructed/Artificial	Germany	Chironomidae	emergence	2,88	1	Field mesocosm	BACI	1	5	discrete	Probably low
Allgeier (2019)	Temporary flooded wetland	Germany	Chironomidae	emergence	1,44	2	Experimental field study	CI	1	16	discrete	Probably low
Allgeier (2019)	Temporary flooded wetland	Germany	Chironomidae	emergence	1,2	1	Control programme field study	CI	1	6	discrete	Probably high
Allgeier (2019)	Temporary flooded wetland	Germany	Chironomidae	emergence	2,88	1	Control programme field study	BACI	1	14	discrete	Probably high
Allgeier (2019b)	Constructed/Artificial	Germany	Chironomidae	emergence	2,88	1	Field mesocosm	CI	1	1	integrated	Probably low
Allgeier (2019b)	Constructed/Artificial	Germany	Chironomidae	emergence	2,88	1	Field mesocosm	CI	1	1	integrated	Probably low
Allgeier (2019b)	Constructed/Artificial	Germany	Chironomidae	emergence	2,88	1	Field mesocosm	CI	1	1	integrated	Probably low
Allgeier (2019b)	Constructed/Artificial	Germany	Chironomidae	emergence	2,88	2	Field mesocosm	CI	1	6	discrete	Probably low
Duchet (2015)	Temporary flooded wetland	France	Polypedilum nubifer	cumulative emergence	3	1	Experimental field study	CI	1	9	discrete	Moderate
Duchet (2015)	Temporary flooded wetland	France	Tanytarsus curticornis	cumulative emergence	3	1	Experimental field study	CI	1	9	discrete	Moderate
Liber (1998)	Permanent wetland	Minnesota, USA	Chironominae	emergence	9	2	Experimental field study	BACI	1	4	discrete	Low
Liber (1998)	Permanent wetland	Minnesota, USA	Orthocladiinae	emergence	9	2	Experimental field study	BACI	1	4	discrete	Low
Liber (1998)	Permanent wetland	Minnesota, USA	Tanypodinae	emergence	9	2	Experimental field study	BACI	1	4	discrete	Low
Lundström (2010b)	Temporary flooded wetland	Sweden	Chironomidae	emergence	3	0-2	Experimental field study	CI	6	6	integrated	Probably high
Pont (1999)	Temporary oligohaline pool	France	Chironomidae	emergence	4,8	1	Experimental field study	CI	1	6	discrete	Probably low
Theissinger (2018)	Temporary flooded wetland	Germany	Chironomidae	emergence	1,44	1-2	Control programme field study	CI	1	11	discrete	Unclear

Abundance, Chironomidae

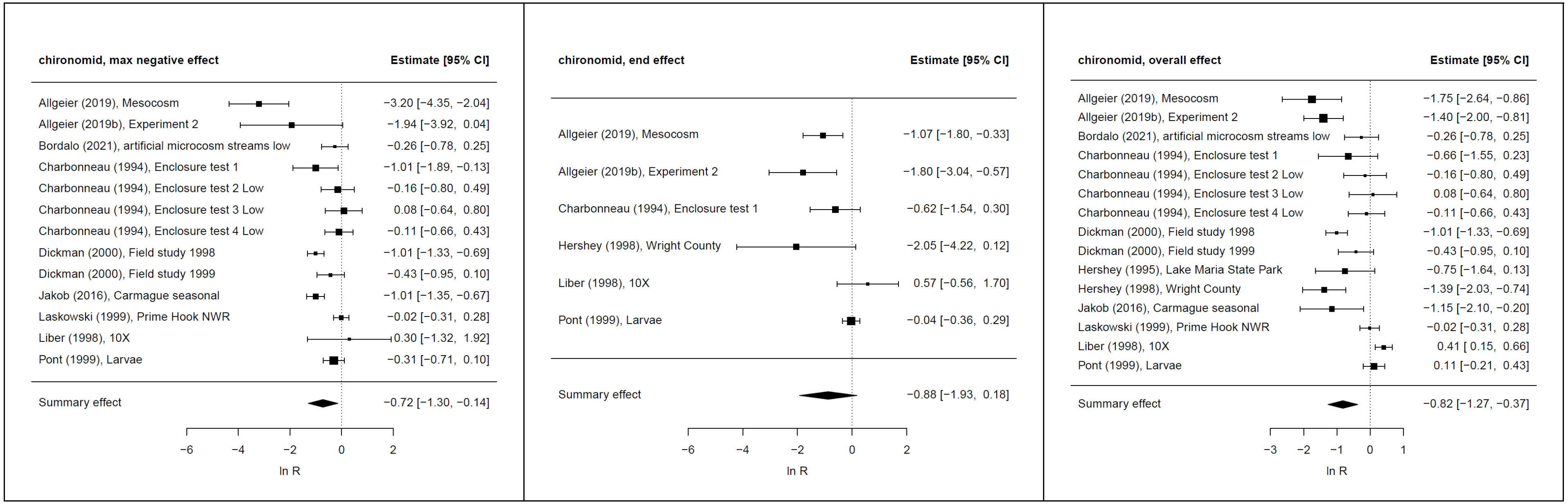

Figure S2. Forest plots showing effects on chironomid abundance in Bti treated areas compared to untreated areas. Summary max negative effect and summary overall effect is significant ($p<0.05$). Bordalo (2021) and Liber (1998) reported data for multiple species in the same study (see **Table S4**). In those cases, we used only one of the species to avoid statistical dependence, and to obtain a conservative estimate of negative effects we selected the species showing the least negative effect (results do not change significantly if other species are used).

Table S3. Estimated summary effects (weighted averages across n studies), their 95% confidence intervals and p-values for chironomid abundance. Changes in chironomid abundance in Bti-treated areas are calculated from back-transformed effect sizes and 95% confidence intervals. The I^2-statistic reflects the heterogeneity of study results beyond random sampling error. Fail-safe number according to Rosenberg (2005).

Group	Summary effect	Estimate [95% CI]	p-value	n	Change (%)	I^2 (%)	Fail-safe N
chironomid	Max negative	-0.721 [-1.3, -0.145]	**0.0184**	13	-51 [-73 – -13]	90	174
chironomid	End of study	-0.876 [-1.93, 0.178]	0.0857	6	-58 [-85 – 19]	76	--
chironomid	Overall	-0.820 [-1.27, -0.369]	**0.0016**	15	-56 [-72 – -31]	85	58
chironomid	Max positive	-0.260 [-0.564, 0.0438]	0.0869	13	-23 [-43 – 4.5]	72	--

Table S4. Studies included in meta-analyses of Chironomid abundance. Bti dose is given in 10^9 ITU/ha. Number of Bti applications per year or study is denoted n_T, number of years with reported measurements is denoted n_Y, and number of reported timepoints after first application is denoted n_{tp}.

Short ref	Type of habitat	Country/State	Taxa	Response variable	Bti dose	n_T	Type of study	Exp. design	n_Y	n_{tp}	Type of datapoints	Risk of bias
Allgeier (2019)	Constructed/Artificial	Germany	Chironomidae	density	2,88	1	Field mesocosm	BACI	1	5	discrete	Probably low
Allgeier (2019b)	Constructed/Artificial	Germany	Chironomidae	density	2,88	2	Field mesocosm	BACI	1	6	discrete	Probably low
Bordalo (2021)	River	Portugal	Chironomus	density	0	1	Lab mesocosm	CI	1	1	discrete	Probably low
Bordalo (2021)	River	Portugal	other Chironomidae	density	0	1	Lab mesocosm	CI	1	1	discrete	Probably low
Charbonneau (1994)	Permanent wetland	Wisconsin, USA	Chironomidae	density	1,12	3	Field mesocosm	BACI	1	4	discrete	Probably low
Charbonneau (1994)	Permanent wetland	Wisconsin, USA	Chironomidae	density	1,12	1	Field mesocosm	BACI	1	1	discrete	Probably low
Charbonneau (1994)	Permanent wetland	Wisconsin, USA	Chironomidae	density	1,12	1	Field mesocosm	CI	1	1	discrete	Probably low
Charbonneau (1994)	Permanent wetland	Wisconsin, USA	Chironomidae	density	1,12	1	Field mesocosm	CI	1	1	discrete	Probably low
Dickman (2000)	River	Hongkong	Chironomidae	density	0,144	1	Experimental field study	CI	1	1	discrete	Probably high
Dickman (2000)	River	Hongkong	Chironomidae	density	0,144	52	Experimental field study	CI	1	1	discrete	Probably high
Hershey (1995)	Pond	Minnesota, USA	Chironomidae	density	1,57	6	Experimental field study	CI	1	1	integrated	Probably low
Hershey (1998)	Permanent wetland	Minnesota, USA	Chironomidae	density	2,344	6	Control programme field study	BACI	5	3	integrated	Low
Jakob (2016)	Permanent wetland	France	Chironomidae	number of individuals	3	30-50	Experimental field study	CI	5	3	discrete	Probably high
Laskowski (1999)	Permanent wetland	Ohio, USA	Chironomidae	number of live larvae	3	1	Field mesocosm	CI	1	1	discrete	Probably low
Liber (1998)	Permanent wetland	Minnesota, USA	Dicrotendipes	density	18	2	Experimental field study	BACI	1	2	discrete	Low
Liber (1998)	Permanent wetland	Minnesota, USA	Einfeldia	density	18	2	Experimental field study	BACI	1	2	discrete	Low
Liber (1998)	Permanent wetland	Minnesota, USA	Endochironomus	density	18	2	Experimental field study	BACI	1	2	discrete	Low
Liber (1998)	Permanent wetland	Minnesota, USA	Cladotanytarsus	density	18	2	Experimental field study	BACI	1	2	discrete	Low
Liber (1998)	Permanent wetland	Minnesota, USA	Paratanytarsus	density	18	2	Experimental field study	BACI	1	2	discrete	Low
Liber (1998)	Permanent wetland	Minnesota, USA	Tanytarsus	density	18	2	Experimental field study	BACI	1	2	discrete	Low
Liber (1998)	Permanent wetland	Minnesota, USA	Chironominae	density	18	2	Experimental field study	BACI	1	3	discrete	Low
Liber (1998)	Permanent wetland	Minnesota, USA	Orthocladiinae	density	18	2	Experimental field study	BACI	1	3	discrete	Low
Liber (1998)	Permanent wetland	Minnesota, USA	Tanypodinae	density	18	2	Experimental field study	BACI	1	3	discrete	Low
Pont (1999)	Temporary oligohaline pool	France	Chironomidae	density	2,4	1	Experimental field study	CI	1	4	discrete	Moderate

Abundance, Crustacea

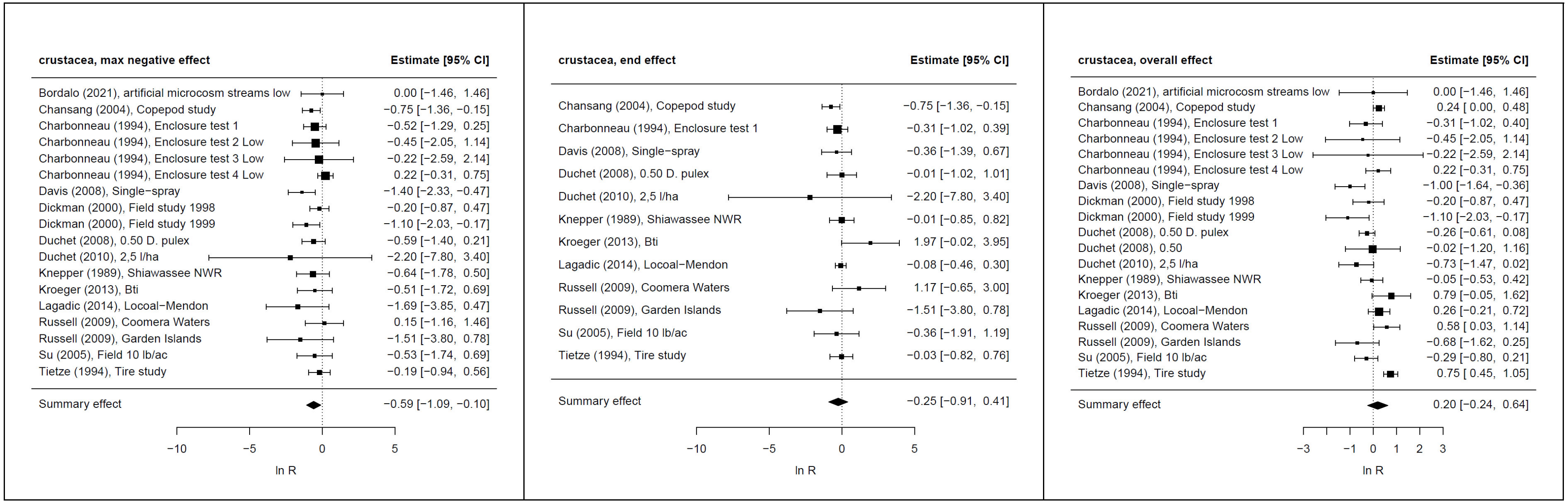

Figure S3. Forest plots showing effects on crustacea abundance in Bti treated areas compared to untreated areas. Summary max negative effect is significant (p<0.05). In cases where multiple taxa groups were reported in the same study, we used only one of the groups to avoid statistical dependence, and to obtain a conservative estimate of negative effects we selected the group showing the least negative effect.

Table S5. Estimated summary effects (weighted averages across n studies), their 95% confidence intervals and p-values for crustacea abundance. Changes in crustacea abundance in Bti-treated areas are calculated from back-transformed effect sizes and 95% confidence intervals. The I^2-statistic reflects the heterogeneity of study results beyond random sampling error. Fail-safe number according to Rosenberg (2005).

Group	Summary effect	Estimate [95% CI]	p-value	n	Change (%)	I^2 (%)	Fail-safe N
crustacea	Max negative	-0.591 [-1.09, -0.0965]	**0.022**	18	-45 [-66 – -9.2]	3	52
crustacea	End	-0.254 [-0.915, 0.407]	0.416	12	-22 [-60 – 50]	19	--
crustacea	Overall	0.196 [-0.245, 0.636]	0.363	19	22 [-22 – 89]	69	--
crustacea	Max positive	0.229 [-0.331, 0.789]	0.401	18	26 [-28 – 120]	71	--

Table S6. Studies included in meta-analyses of Crustacea abundance. Bti dose is given in 10^9 ITU/ha. Number of Bti applications per year or study is denoted n_T, number of years with reported measurements is denoted n_Y, and number of reported timepoints after first application is denoted n_{tp}.

Short ref	Type of habitat	Country/State	Taxa	Response variable	Bti dose	n_T	Type of study	Exp. design	n_Y	n_{tp}	Type of datapoints	Risk of bias
Bordalo (2021)	River	Portugal	Asellus	density	0	1	Lab mesocosm	CI	1	1	discrete	Probably low
Chansang (2004)	Other	Thailand	Mesocyclops thermocyclopoides	number of individuals	0	1	Lab mesocosm	CI	1	16	discrete	Probably low
Charbonneau (1994)	Permanent wetland	Wisconsin, USA	Hyalella azteca	density	1,12	3	Field mesocosm	BACI	1	4	discrete	Probably low
Charbonneau (1994)	Permanent wetland	Wisconsin, USA	Amphipoda	density	1,12	1	Field mesocosm	BACI	1	1	discrete	Probably low
Charbonneau (1994)	Permanent wetland	Wisconsin, USA	Amphipoda	density	1,12	1	Field mesocosm	CI	1	1	discrete	Probably low
Charbonneau (1994)	Permanent wetland	Wisconsin, USA	Gammarus spp	density	1,12	1	Field mesocosm	CI	1	1	discrete	Probably low
Davis (2008)	Pond	Montana, USA	Amphipoda	density	unclear	1	Experimental field study	CI	2	2	discrete	Probably high
Dickman (2000)	River	Hongkong	Decapoda	density	0,144	1	Experimental field study	CI	1	1	discrete	Probably high
Dickman (2000)	River	Hongkong	Decapoda	density	0,144	52	Experimental field study	CI	1	1	discrete	Probably high
Duchet (2008)	Temporary oligohaline pool	France	Daphnia pulex	density	1,5	1	Field mesocosm	BACI	1	5	discrete	Probably low
Duchet (2008)	Temporary oligohaline pool	France	Cladocera	density	1,5	1	Field mesocosm	CI	1	1	integrated	Probably low
Duchet (2010)	Temporary oligohaline pool	France	Daphnia magna	density	3	1	Field mesocosm	BACI	1	5	discrete	Moderate
Knepper (1989)	Other	Michigan, USA	Asellus forbesi	density	1,12	1	Experimental field study	BACI	1	3	discrete	Probably high
Kroeger (2013)	Pond	Germany	cladocera	density	1,8	1	Experimental field study	BACI	1	16	discrete	Probably low
Kroeger (2013)	Pond	Germany	ostracoda	density	1,8	1	Experimental field study	BACI	1	16	discrete	Probably low
Kroeger (2013)	Pond	Germany	cyclopoida	density	1,8	1	Experimental field study	BACI	1	16	discrete	Probably low
Lagadic (2014)	Permanent wetland	France	Crustaceans	density	0,85	4-10	Control programme field study	CI	7	35	discrete	Probably high
Russell (2009)	Temporary flooded wetland	Australia	Copepods	number of individuals	1,44	1	Experimental field study	CI	1	2	discrete	High
Russell (2009)	Temporary flooded wetland	Australia	Copepods	number of individuals	1,44	1	Experimental field study	CI	1	2	discrete	High
Su (2005)	Pond	California, USA	Triops newberryi	density	2,22	1	Field mesocosm	BACI	1	3	discrete	Unclear
Tietze (1994)	Other	Florida, USA	mesocyclops	density	0	2	Field mesocosm	BACI	1	21	discrete	Probably low

Abundance, Mollusca

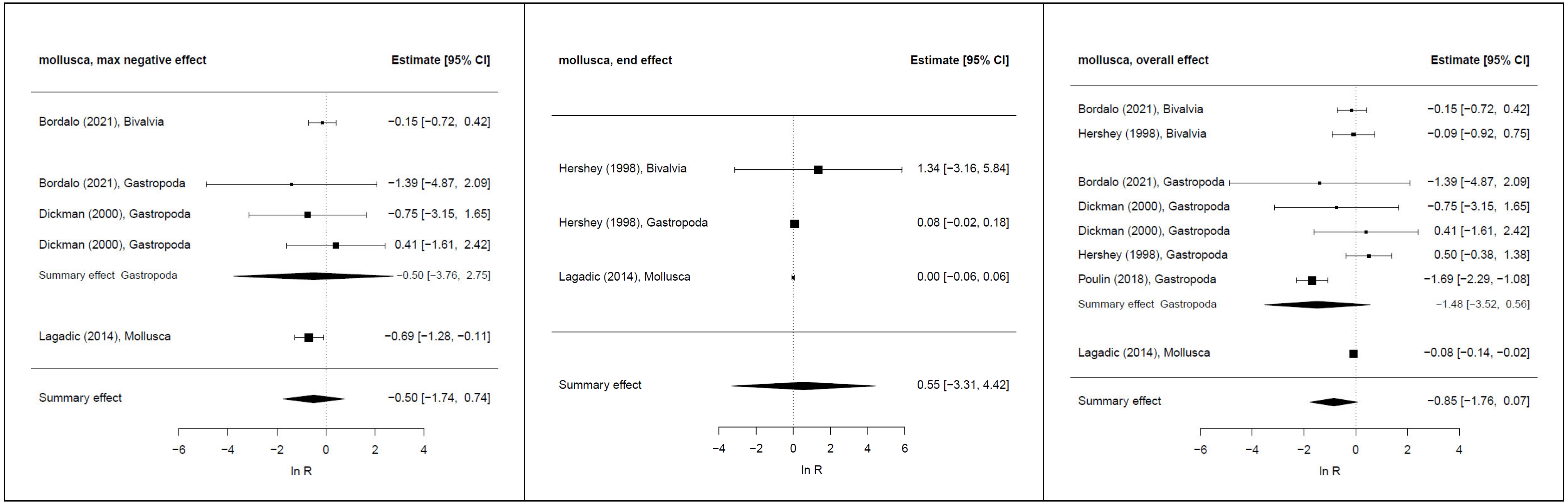

Figure S4. Forest plots showing effects on mollusca abundance in Bti treated areas compared to untreated areas. No summary effect is significant (p>0.05). Dickman (2000) conducted two independent studies.

Table S7. Estimated summary effects (weighted averages across n studies), their 95% confidence intervals and p-values for Mollusca abundance. Changes in Mollusca abundance in Bti-treated areas are calculated from back-transformed effect sizes and 95% confidence intervals.

Group	Summary effect	Estimate [95% CI]	p-value	n	Change (%)
mollusca	Max negative	-0.498 [-1.74, 0.744]	0.328	5	-39 [-82 – 110]
mollusca	End	0.554 [-3.31, 4.42]	0.6	3	74 [-96 – 8200]
mollusca	Overall	-0.846 [-1.76, 0.0681]	0.0648	8	-57 [-83 – 7]
mollusca	Max positive	-0.275 [-1.53, 0.977]	0.574	5	-24 [-78 – 170]

Table S8. Studies included in meta-analyses of Mollusca abundance. Bti dose is given in 10^9 ITU/ha. Number of Bti applications per year or study is denoted n_T, number of years with reported measurements is denoted n_Y, and number of reported timepoints after first application is denoted n_{tp}.

Short ref	Type of habitat	Country/State	Taxa	Response variable	Bti dose	n_T	Type of study	Exp. design	n_Y	n_{tp}	Type of datapoints	Risk of bias
Bordalo (2021)	River	Portugal	Ancylus	density		1	Lab mesocosm	CI	1	1	discrete	Probably low
Bordalo (2021)	River	Portugal	Pisidium	density		1	Lab mesocosm	CI	1	1	discrete	Probably low
Dickman (2000)	River	Hongkong	Gastropoda	density	0,144	1	Experimental field study	CI	1	1	discrete	Probably high
Dickman (2000)	River	Hongkong	Gastropoda	density	0,144	52	Experimental field study	CI	1	1	discrete	Probably high
Hershey (1998)	Permanent wetland	Minnesota, USA	Total Gastropoda	density	2,344	6	Control programme field study	BACI	5	3	integrated	Low
Hershey (1998)	Permanent wetland	Minnesota, USA	Total Bivalvia	density	2,344	6	Control programme field study	BACI	5	3	integrated	Low
Lagadic (2014)	Permanent wetland	France	Moluscs	density	0,85	4-10	Control programme field study	CI	7	36	discrete	Probably high
Poulin (2018)	Permanent wetland	France	Gastropoda	number of individuals	3	30-50	Control programme field study	CI	9	1	integrated	Moderate

Diversity, taxa richness

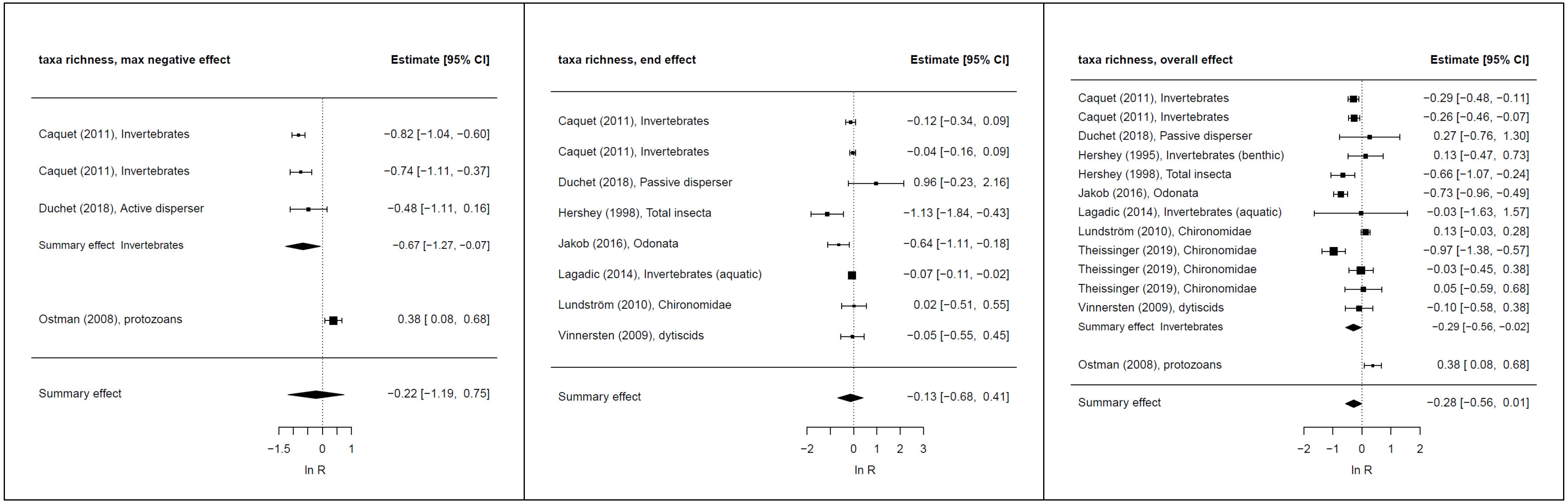

Figure S5. Forest plots showing effects on taxa richness in Bti treated areas compared to untreated areas. Summary max negative effect and summary overall effect are significant (p<0.05) for invertebrates. In Duchet (2018), active and passive dispersers include insects (Culicidae, Chironomidae, Ceratopogonidae, Ephemeroptera), and crustaceans (calanoid copepods, cladocerans, och ostracods), respectively. In each meta-analysis we used only one of the groups to avoid statistical dependence and to obtain a conservative estimate of negative effects we selected the group showing the least negative effect. From Hershey (1998) we selected the broadest taxa group reported (total insects).

Table S9. Estimated summary effects (weighted averages across n studies), their 95% confidence intervals and p-values for taxa richness. Changes in taxa richness in Bti-treated areas are calculated from back-transformed effect sizes and 95% confidence intervals. The I^2-statistic reflects the heterogeneity of study results beyond random sampling error. Fail-safe number according to Rosenberg (2005).

Group	Summary effect	Estimate [95% CI]	p-value	n	Change (%)	I^2 (%)	Fail-safe N
taxa richness	Max negative	-0.221 [-1.19, 0.746]	0.52	4	-20 [-70 – 110]	91	--
taxa richness	End of study	-0.134 [-0.682, 0.414]	0.58	8	-13 [-49 – 51]	94	--
taxa richness	Overall	-0.28 [-0.565, 0.00532]	0.0538	13	-24 [-43 – 0.53]	83	--
taxa richness	Max positive	0.363 [-0.257, 0.984]	0.159	4	44 [-23 – 170]	67	--

Table S10. Studies included in meta-analyses of species/taxa richness. Bti dose is given in 10^9 ITU/ha. Number of Bti applications per year or study is denoted n_T, number of years with reported measurements is denoted n_Y, and number of reported timepoints after first application is denoted n_{tp}. Caquet (2011) conducted two independent studies using different Bti formulations.

Short ref	Type of habitat	Country/State	Taxa	Response variable	Bti dose	n_T	Type of study	Exp. design	n_Y	n_{tp}	Type of datapoints	Risk of bias
Caquet (2011)	Temporary oligohaline pool	France	Invertebrates	taxonomic richness	0,6	5-8	Control programme field study	BACI	2	8	discrete	High
Caquet (2011)	Temporary oligohaline pool	France	Invertebrates	taxonomic richness	1,2	5-8	Control programme field study	BACI	2	8	discrete	High
Duchet (2018)	Pond	Israel	Active disperser	species richness	32,4	2	Field mesocosm	CI	1	3	discrete	Low
Duchet (2018)	Pond	Israel	Passive disperser	species richness	32,4	2	Field mesocosm	CI	1	3	discrete	Low
Hershey (1995)	Pond	Minnesota, USA	aquatic invertebrates	species richness	1,57	6	Experimental field study	CI	1	1	integrated	Probably low
Hershey (1998)	Permanent wetland	Minnesota, USA	Total insecta	taxonomic richness	2,344	6	Control programme field study	BACI	5	3	integrated	Low
Hershey (1998)	Permanent wetland	Minnesota, USA	Chironomidae	taxonomic richness	2,344	6	Control programme field study	BACI	5	3	integrated	Low
Jakob (2016)	Permanent wetland	France	Odonata	species richness	3	30-50	Experimental field study	CI	5	6	integrated	Probably high
Lagadic (2014)	Permanent wetland	France	aquatic invertebrates	taxonomic richness	0,85	4-10	Control programme field study	CI	7	3	integrated	Probably high
Lundström (2010)	Temporary flooded wetland	Sweden	Chironomidae	species richness	Not reported	unclear	Control programme field study	CI	6	6	integrated	Moderate
Ostman (2008)	Temporary flooded wetland	Sweden	protozoans	taxonomic richness	3	unclear	Control programme field study	CI	1	1	discrete	Probably low
Theissinger (2019)	Temporary flooded wetland	Germany	Chironomidae	taxonomic richness	2,88	1	Control programme field study	BACI	1	1	integrated	Probably high
Theissinger (2019)	Temporary flooded wetland	Germany	Chironomidae	taxonomic richness	1,44	2	Experimental field study	CI	1	1	integrated	Probably low
Theissinger (2019)	Temporary flooded wetland	Germany	Chironomidae	taxonomic richness	1,2	1	Control programme field study	CI	1	1	integrated	Probably high
Vinnersten (2009)	Temporary flooded wetland	Sweden	dytiscids	species richness	2,8	0-2	Experimental field study	CI	5	6	integrated	Probably high

Species traits, size

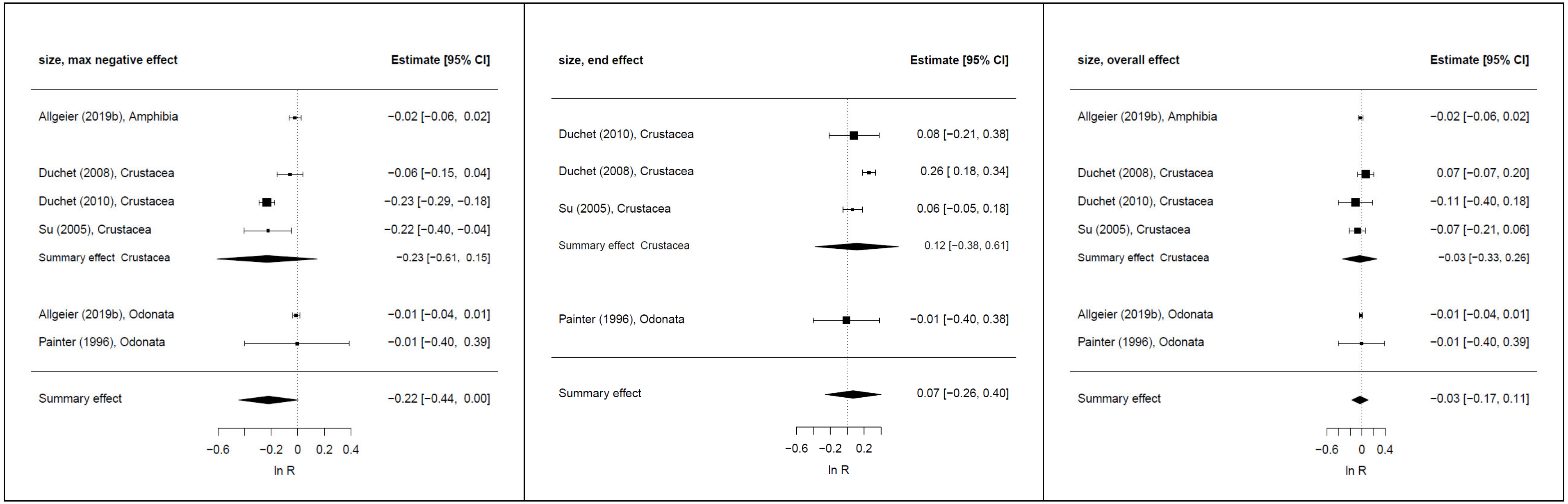

Figure S6. Forest plots showing effects on size in Bti treated areas compared to untreated areas. No summary effect is significant (p<0.05). Painter (1996) reported multiple response variables on the same individuals. In each meta-analysis we used only one of the response variables to avoid statistical dependence, and to obtain a conservative estimate of negative effects we selected the variable showing the least negative effect. Allgeier (2019b) conducted two independent studies for Amphibia and Odonata, respectively.

Table S11. Estimated summary effects (weighted averages across n studies), their 95% confidence intervals and p-values for size. Changes in size in Bti-treated areas are calculated from back-transformed effect sizes and 95% confidence intervals.

Group	Summary effect	Estimate [95% CI]	p-value	n	Change (%)	I^2 (%)	Fail-safe N
size	Max negative	-0.22 [-0.444, 0.00459]	0.0533	6	-20 [-36 – 0.46]	89	--
size	End	0.0713 [-0.26, 0.403]	0.543	4	7.4 [-23 – 50]	51	--
size	Overall	-0.0299 [-0.17, 0.111]	0.608	6	-2.9 [-16 – 12]	0	--
size	Max positive	0.0491 [-0.163, 0.261]	0.577	6	5 [-15 – 30]	89	--

Table S12. Studies included in meta-analyses of size-related response variables. Bti dose is given in 10^9 ITU/ha. Number of Bti applications per year or study is denoted n_T, number of years with reported measurements is denoted n_Y, and number of reported timepoints after first application is denoted n_{tp}.

Short ref	Type of habitat	Country/State	Taxa	Response variable	Bti dose	n_T	Type of study	Exp. design	n_Y	n_{tp}	Type of datapoints	Risk of bias
Allgeier (2019b)	Constructed/Artificial	Germany	Aeshna cyanea	length	2,88	1	Field mesocosm	CI	1	1	discrete	Probably low
Allgeier (2019b)	Constructed/Artificial	Germany	Lissotriton helveticus and Lissotriton vulgaris.	length	2,88	2	Field mesocosm	CI	1	1	discrete	Probably low
Duchet (2008)	Temporary oligohaline pool	France	Daphnia pulex	body size	1,5	1	Field mesocosm	CI	1	5	discrete	Probably low
Duchet (2010)	Temporary oligohaline pool	France	Daphnia magna	body length	3	1	Field mesocosm	BACI	1	5	discrete	Moderate
Painter (1996)	Other	Tennessee, USA	Erythemis simplicicollis	hind femur length	0,70	8	Lab mesocosm	CI	1	6	discrete	Probably low
Painter (1996)	Other	Tennessee, USA	Erythemis simplicicollis	adult male hind wing length	0,70	8	Lab mesocosm	CI	1	1	discrete	Probably low
Painter (1996)	Other	Tennessee, USA	Erythemis simplicicollis	adult female hind wing length	0,70	8	Lab mesocosm	CI	1	1	discrete	Probably low
Su (2005)	Pond	California, USA	Triops newberryi	length	2,22	1	Field mesocosm	BACI	1	3	discrete	Unclear

Species traits, weight

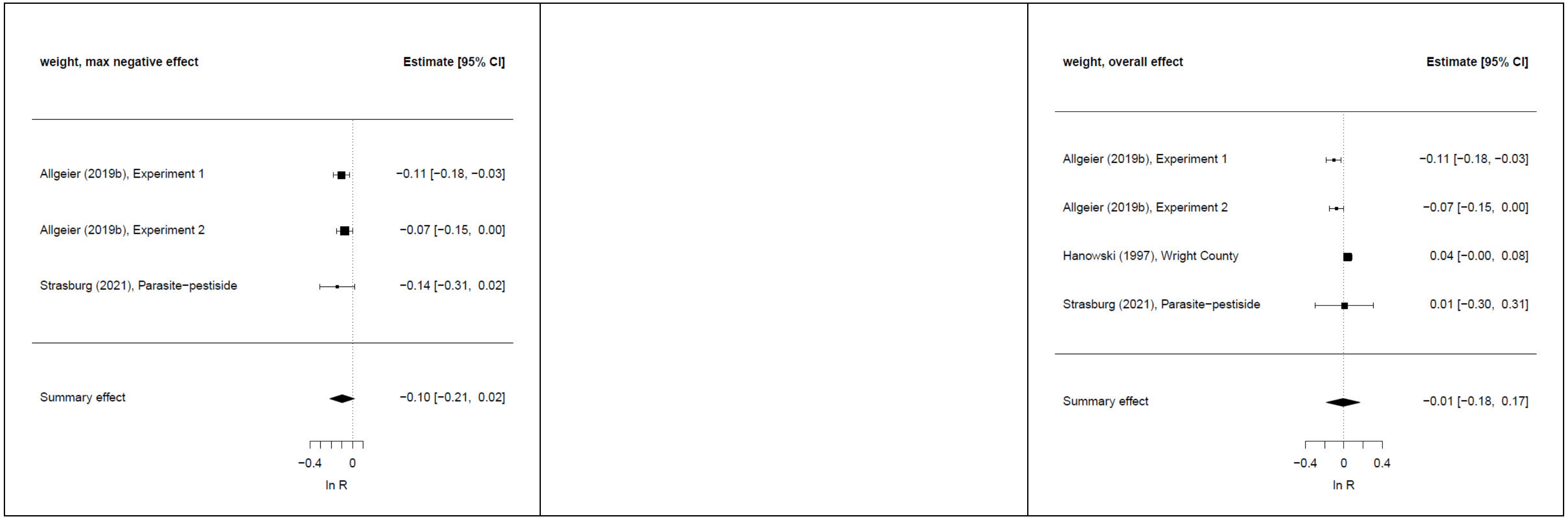

Figure S7. Forest plots showing effects on weight in Bti treated areas compared to untreated areas. No summary effect is significant (p>0.05). Meta-analysis for the effect at the end of the studies was not conducted since the number of studies with more than one reported timepoint were too low.

Table S13. Estimated summary effects (weighted averages across n studies), their 95% confidence intervals and p-values for weight. Changes in size in Bti-treated areas are calculated from back-transformed effect sizes and 95% confidence intervals.

Group	Summary effect	Estimate [95% CI]	p-value	n	Change (%)
weight	Max negative	-0.0983 [-0.214, 0.0172]	0.0671	3	-9.4 [-19 – 1.7]
weight	End of study	0.0989 [-0.8, 0.998]	0.395	2	10 [-55 – 170]
weight	Overall	-0.00696 [-0.182, 0.168]	0.908	4	-0.69 [-17 – 18]
weight	Max positive	-0.0406 [-0.226, 0.145]	0.446	3	-4 [-20 – 16]

Table S14. Studies included in meta-analyses of weight-related response variables. Bti dose is given in 10^9 ITU/ha. Number of Bti applications per year or study is denoted n_T, number of years with reported measurements is denoted n_Y, and number of reported timepoints after first application is denoted n_{tp}.

Short ref	Type of habitat	Country/State	Taxa	Response variable	Bti dose	n_T	Type of study	Exp. design	n_Y	n_{tp}	Type of datapoints	Risk of bias
Allgeier (2019b)	Constructed/Artificial	Germany	Aeshna cyanea	weight	2,88	1	Field mesocosm	CI	1	1	discrete	Probably low
Allgeier (2019b)	Constructed/Artificial	Germany	Lissotriton helveticus and Lissotriton vulgaris.	weight	2,88	2	Field mesocosm	CI	1	1	discrete	Probably low
Hanowski (1997)	Permanent wetland	Minnesota, USA	Agelaius phoeniceus	male weight	unclear	2	Experimental field study	CI	6	3	integrated	Unclear
Strasburg (2021)	Pond	Ohio, USA	Lithobates pipiens	mass at metamorphosis		1	Field mesocosm	CI	1	2	discrete	Low

Species traits, feeding rate

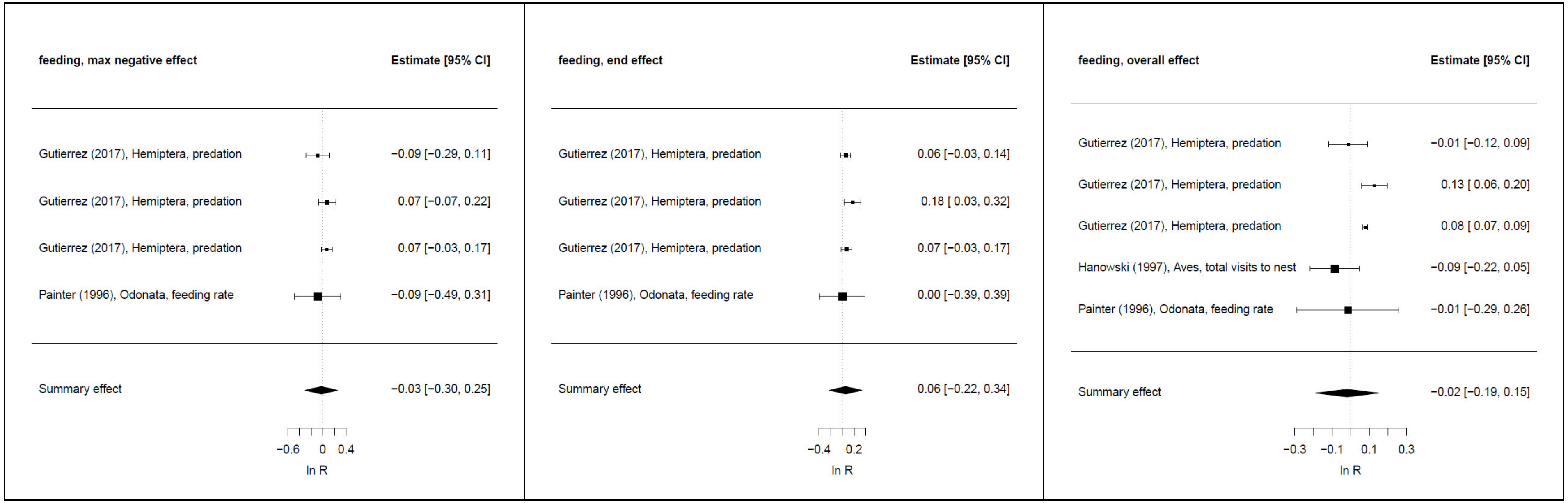

Figure S8. Forest plots showing effects on feeding rate in Bti treated areas compared to untreated areas. No summary effect is significant (p>0.05). Gutierrez (2017) conducted three independent studies.

Table S15. Estimated summary effects (weighted averages across n studies), their 95% confidence intervals and p-values for feeding rate. Changes in feeding rate in Bti-treated areas are calculated from back-transformed effect sizes and 95% confidence intervals.

Group	Summary effect	Estimate [95% CI]	p-value	n	Change (%)
feeding	Max negative	-0.0252 [-0.304, 0.253]	0.792	4	-2.5 [-26 – 29]
feeding	End	0.0573 [-0.221, 0.335]	0.559	4	5.9 [-20 – 40]
feeding	Overall	-0.0189 [-0.188, 0.15]	0.772	5	-1.9 [-17 – 16]
feeding	Max positive	0.0728 [-0.209, 0.355]	0.471	4	7.6 [-19 – 43]

Table S16. Studies included in meta-analyses of feeding rate. Bti dose is given in 10^9 ITU/ha. Number of Bti applications per year or study is denoted n_T, number of years with reported measurements is denoted n_Y, and number of reported timepoints after first application is denoted n_{tp}.

Short ref	Type of habitat	Country/State	Taxa	Response variable	Bti dose	n_T	Type of study	Exp. design	n_Y	n_{tp}	Type of datapoints	Risk of bias
Gutierrez (2017)	Constructed/Artificial	Brazil	Buenoa tarsalis	predation		1	Lab mesocosm	CI	1	2	discrete	Unclear
Gutierrez (2017)	Constructed/Artificial	Brazil	Buenoa tarsalis	predation		1	Lab mesocosm	CI	1	2	discrete	Unclear
Gutierrez (2017)	Constructed/Artificial	Brazil	Buenoa tarsalis	predation		1	Lab mesocosm	CI	1	2	discrete	Unclear
Hanowski (1997)	Permanent wetland	Minnesota, USA	Agelaius phoeniceus	total visits to nest	unclear	2	Experimental field study	CI	6	1	integrated	Unclear
Painter (1996)	Other	Tennessee, USA	Erythemis simplicicollis	feeding rate	0,70	8	Lab mesocosm	CI	1	6	discrete	Probably low

Environmental data, pH

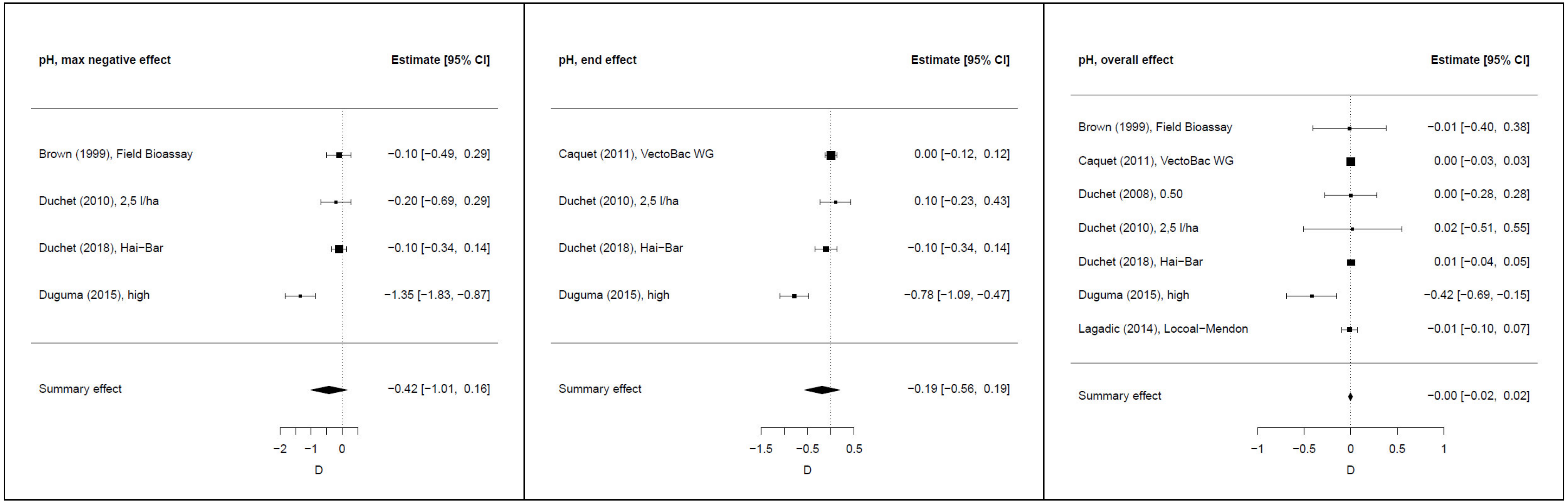

Figure S9. Forest plots showing effects on pH in Bti treated areas compared to untreated areas. No summary effect is significant (p>0.05).

Table S17. Estimated summary effects (weighted averages across n studies), their 95% confidence intervals and p-values for pH. Changes in pH in Bti-treated areas are calculated from back-transformed effect sizes and 95% confidence intervals.

Group	Summary effect	Estimate [95% CI]	p-value	n	Change (%)
pH	Max negative	-0.423 [-1.01, 0.161]	0.156	4	not calculated
pH	End of study	-0.188 [-0.565, 0.188]	0.327	4	not calculated
pH	Overall	-0.00216 [-0.0238, 0.0195]	0.845	7	not calculated
pH	Max positive	0.184 [-0.00942, 0.377]	0.0623	4	not calculated

Table S18. Studies included in meta-analyses of pH. Bti dose is given in 10^9 ITU/ha. Number of Bti applications per year or study is denoted n_T, number of years with reported measurements is denoted n_Y, and number of reported timepoints after first application is denoted n_{tp}.

Short ref	Type of habitat	Country/State	Response variable	Bti dose	n_T	Type of study	Exp. design	n_Y	n_{tp}	Type of datapoints	Risk of bias
Brown (1999)	Temporary oligohaline pool	Australia	pH	1,279	1	Field mesocosm	CI	1	1	discrete	Moderate
Caquet (2011)	Temporary oligohaline pool	France	pH	1,2	5-8	Control programme field study	CI	2	2	integrated	High
Duchet (2008)	Temporary oligohaline pool	France	pH	1,5	1	Field mesocosm	CI	1	1	integrated	Probably low
Duchet (2010)	Temporary oligohaline pool	France	pH	3	1	Field mesocosm	BACI	1	5	discrete	Moderate
Duchet (2018)	Pond	Israel	pH	32,4	2	Field mesocosm	CI	1	4	discrete	Low
Duguma (2015)	Pond	California, USA	pH	9,62	1	Lab mesocosm	BACI	1	6	discrete	Probably low
Lagadic (2014)	Permanent wetland	France	pH	0,85	4-10	Control programme field study	CI	7	1	integrated	Probably high

Environmental data, dissolved oxygen (DO)

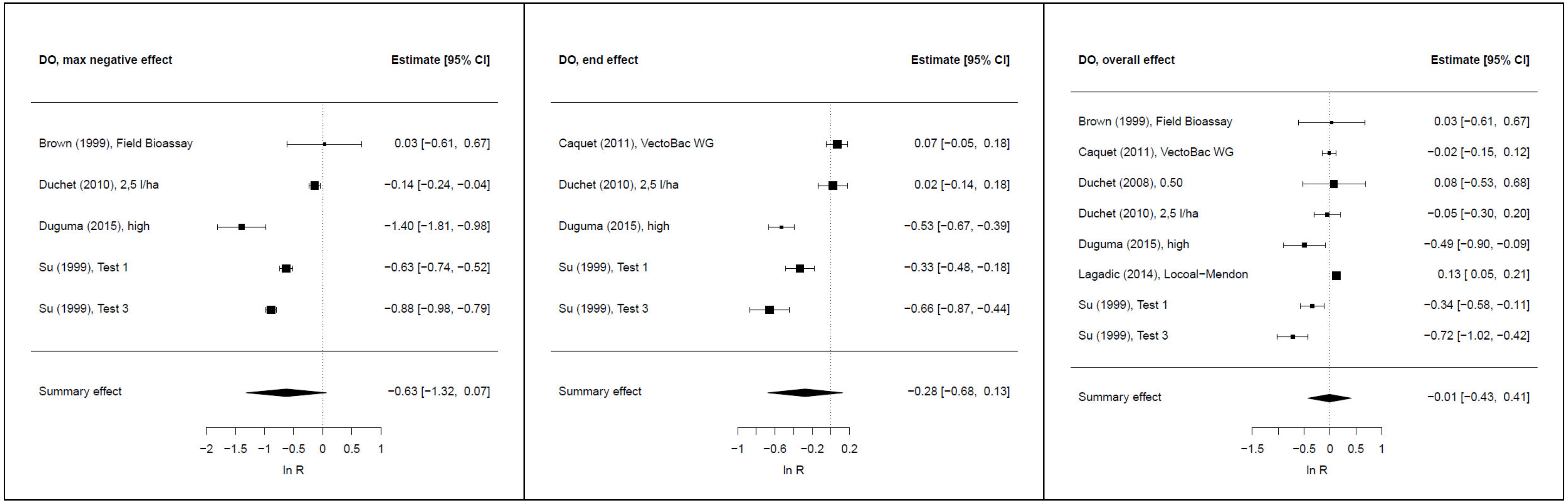

Figure S10. Forest plots showing effects on DO in Bti treated areas compared to untreated areas. No summary effect is significant (p>0.05).

Table S19. Estimated summary effects (weighted averages across n studies), their 95% confidence intervals and p-values for DO. Changes in DO in Bti-treated areas are calculated from back-transformed effect sizes and 95% confidence intervals.

Group	Summary effect	Estimate [95% CI]	p-value	n	Change (%)
DO	Max negative	-0.625 [-1.32, 0.0662]	0.066	5	-46 [-73 – 6.8]
DO	End of study	-0.276 [-0.678, 0.127]	0.13	5	-24 [-49 – 14]
DO	Overall	-0.0088 [-0.426, 0.409]	0.962	8	-0.88 [-35 – 51]
DO	Max positive	-0.124 [-0.513, 0.266]	0.428	5	-12 [-40 – 30]

Table S20. Studies included in meta-analyses of dissolved oxygen (DO). Bti dose is given in 10^9 ITU/ha. Number of Bti applications per year or study is denoted n_T, number of years with reported measurements is denoted n_Y, and number of reported timepoints after first application is denoted n_{tp}.

Short ref	Type of habitat	Country/State	Response variable	Bti dose	n_T	Type of study	Exp. design	n_Y	n_{tp}	Type of datapoints	Risk of bias
Brown (1999)	Temporary oligohaline pool	Australia	DO	1,279	1	Field mesocosm	CI	1	1	discrete	Moderate
Caquet (2011)	Temporary oligohaline pool	France	DO	1,2	5-8	Control programme field study	CI	2	2	integrated	High
Duchet (2008)	Temporary oligohaline pool	France	DO	1,5	1	Field mesocosm	CI	1	1	integrated	Probably low
Duchet (2010)	Temporary oligohaline pool	France	DO	3	1	Field mesocosm	BACI	1	5	discrete	Moderate
Duguma (2015)	Pond	California, USA	DO	9,62	1	Lab mesocosm	BACI	1	6	discrete	Probably low
Lagadic (2014)	Permanent wetland	France	DO	0,85	4-10	Control programme field study	CI	7	1	integrated	Probably high
Su (1999)	Built environment	California, USA	DO	9,62	1	Lab mesocosm	BACI	1	5	discrete	Moderate
Su (1999)	Built environment	California, USA	DO	0,21	1	Lab mesocosm	BACI	1	4	discrete	Moderate

Environmental data, chlorophyll a

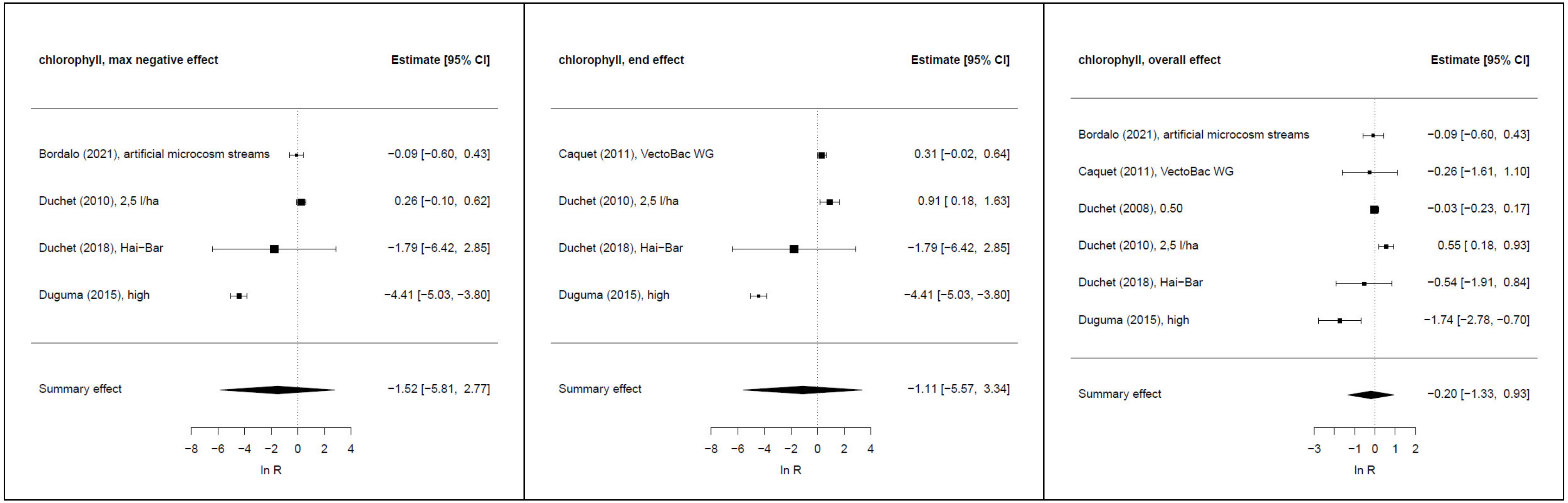

Figure S11. Forest plots showing effects on chlorophyll a in Bti treated areas compared to untreated areas. No summary effect is significant (p>0.05).

Table S21. Estimated summary effects (weighted averages across n studies), their 95% confidence intervals and p-values for chlorophyll a. Changes in chlorophyll a in Bti-treated areas are calculated from back-transformed effect sizes and 95% confidence intervals.

Group	Summary effect	Estimate [95% CI]	p-value	n	Change (%)
chlorophyll a	Max negative	-1.52 [-5.81, 2.77]	0.341	4	-78 [-100 – 1500]
chlorophyll a	End of study	-1.11 [-5.57, 3.34]	0.484	4	-67 [-100 – 2700]
chlorophyll a	Overall	-0.197 [-1.33, 0.932]	0.672	6	-18 [-74 – 150]
chlorophyll a	Max positive	-0.124 [-0.513, 0.266]	0.428	5	-12 [-40 – 30]

Table S22. Studies included in meta-analyses of chlorophyll a. Bti dose is given in 10^9 ITU/ha. Number of Bti applications per year or study is denoted n_T, number of years with reported measurements is denoted n_Y, and number of reported timepoints after first application is denoted n_{tp}.

Short ref	Type of habitat	Country/State	Response variable	Bti dose	n_T	Type of study	Exp. design	n_Y	n_{tp}	Type of datapoints	Risk of bias
Bordalo (2021)	River	Portugal	chlorophyll a	0	1	Lab mesocosm	CI	1	1	discrete	Probably low
Caquet (2011)	Temporary oligohaline pool	France	chlorophyll a	1,2	5-8	Control programme field study	CI	2	2	integrated	High
Duchet (2008)	Temporary oligohaline pool	France	chlorophyll a	1,5	1	Field mesocosm	CI	1	1	integrated	Probably low
Duchet (2010)	Temporary oligohaline pool	France	chlorophyll a	3	1	Field mesocosm	BACI	1	5	discrete	Moderate
Duchet (2018)	Pond	Israel	chlorophyll a	32,4	2	Field mesocosm	CI	1	3	discrete	Low
Duguma (2015)	Pond	California, USA	chlorophyll a	9,62	1	Lab mesocosm	BACI	1	5	discrete	Probably low

Environmental data, suspended particles

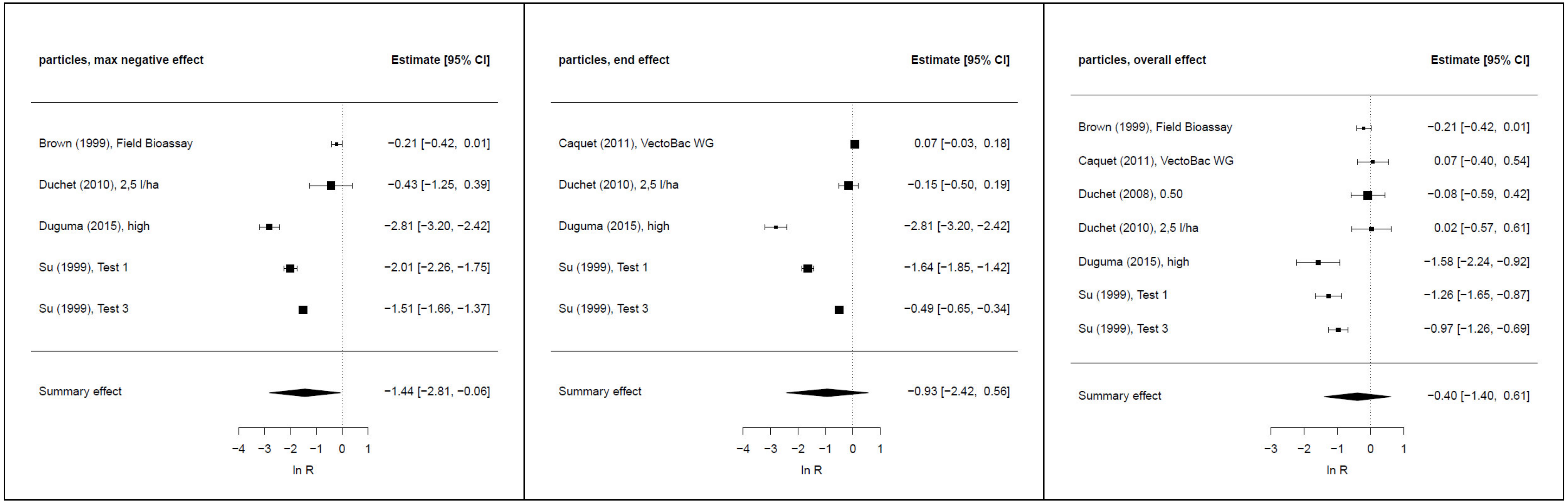

Figure S12. Forest plots showing effects on suspended particles in Bti treated areas compared to untreated areas. Summary max negative effect is significant (p<0.05).

Table S23. Estimated summary effects (weighted averages across n studies), their 95% confidence intervals and p-values for suspended particles. Changes in suspended particles in Bti-treated areas are calculated from back-transformed effect sizes and 95% confidence intervals.

Group	Summary effect	Estimate [95% CI]	p-value	n	Change (%)	I^2 (%)	Fail-safe N
particles	Max negative	-1.44 [-2.81, -0.0611]	**0.0441**	5	-76 [-94 – -5.9]	99	855
particles	End of study	-0.93 [-2.42, 0.565]	0.159	5	-61 [-91 – 76]	99	--
particles	Overall	-0.396 [-1.4, 0.609]	0.372	7	-33 [-75 – 84]	91	--
particles	Max positive	-0.228 [-0.826, 0.37]	0.35	5	-20 [-56 – 45]	93	--

Table S24. Studies included in meta-analyses of suspended particles. Bti dose is given in 10^9 ITU/ha. Number of Bti applications per year or study is denoted n_T, number of years with reported measurements is denoted n_Y, and number of reported timepoints after first application is denoted n_{tp}..

Short ref	Type of habitat	Country/State	Response variable	Bti dose	n_T	Type of study	Exp. design	n_Y	n_{tp}	Type of datapoints	Risk of bias
Brown (1999)	Temporary oligohaline pool	Australia	turbidity	1,279	1	Field mesocosm	CI	1	1	discrete	Moderate
Caquet (2011)	Temporary oligohaline pool	France	SS	1,2	5-8	Control programme field study	CI	2	2	integrated	High
Duchet (2008)	Temporary oligohaline pool	France	Suspended matter	1,5	1	Field mesocosm	CI	1	1	integrated	Probably low
Duchet (2010)	Temporary oligohaline pool	France	suspended matter	3	1	Field mesocosm	BACI	1	5	discrete	Moderate
Duguma (2015)	Pond	California, USA	particles	9,62	1	Lab mesocosm	BACI	1	5	discrete	Probably low
Su (1999)	Built environment	California, USA	optical density	9,62	1	Lab mesocosm	BACI	1	5	discrete	Moderate
Su (1999)	Built environment	California, USA	optical density	0,21	1	Lab mesocosm	BACI	1	4	discrete	Moderate

Funnel plots, chironomid emergence

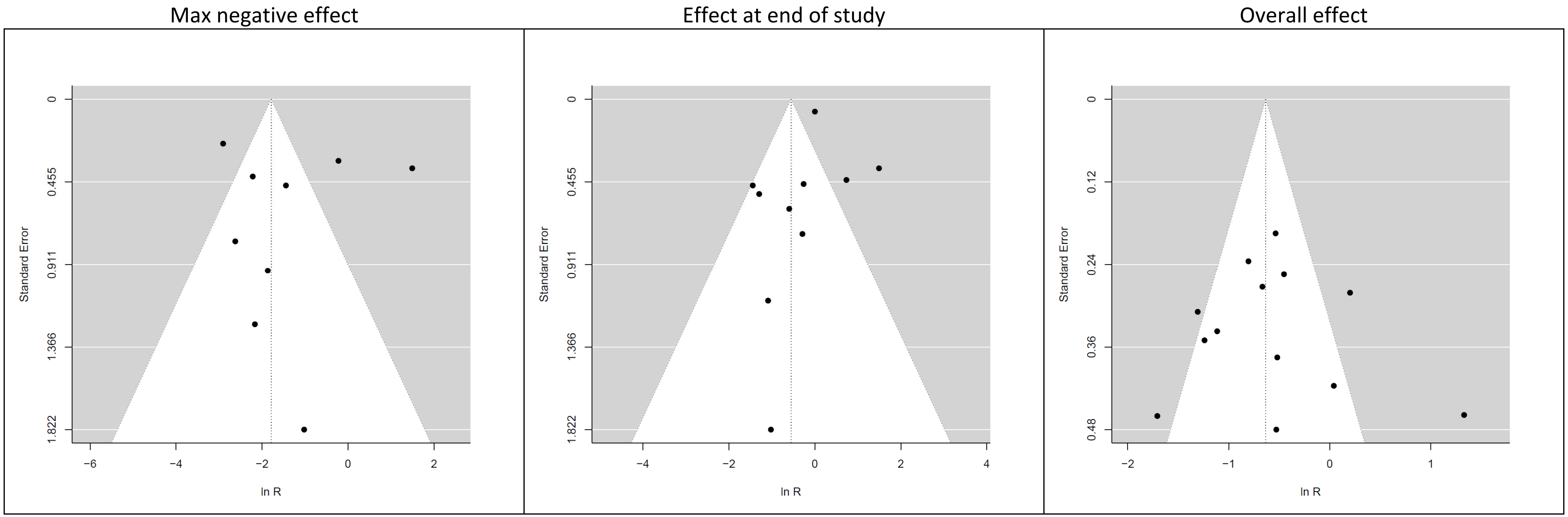

Figure S13. Funnel plots for effect sizes of chironomid emergence. There is no obvious asymmetry that could indicate possible publication bias.

Funnel plots, chironomid abundance

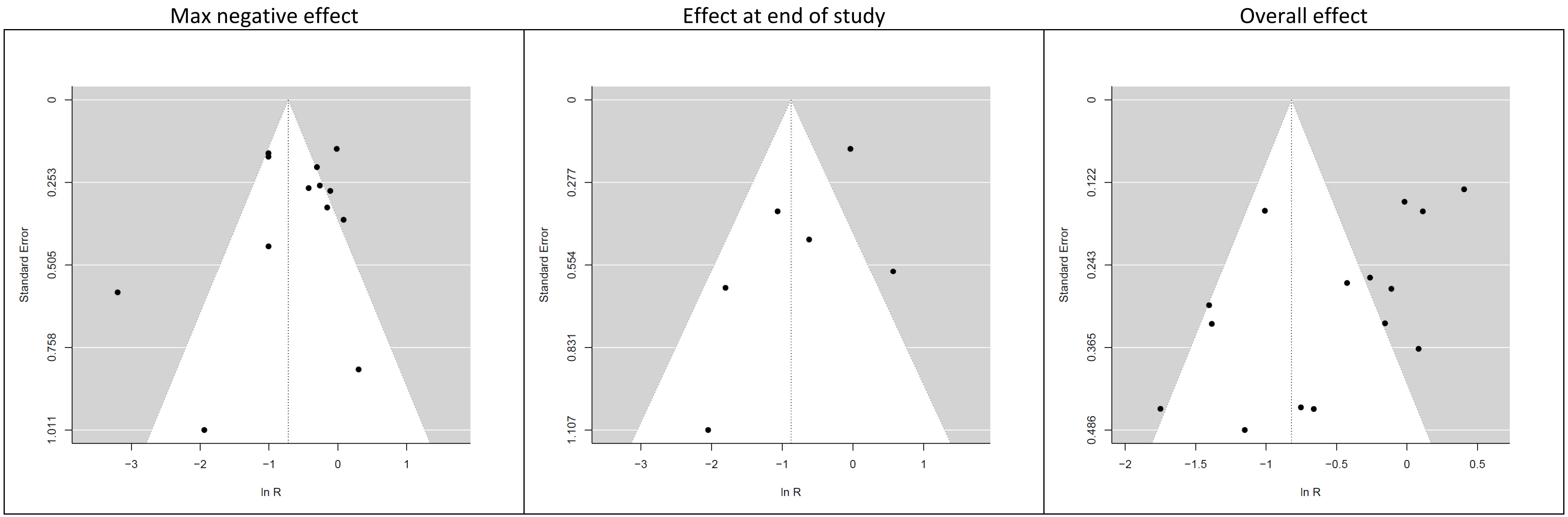

Figure S14. Funnel plots for effect sizes of chironomid abundance. There is no obvious asymmetry that could indicate possible publication bias.

Funnel plots, crustacea abundance

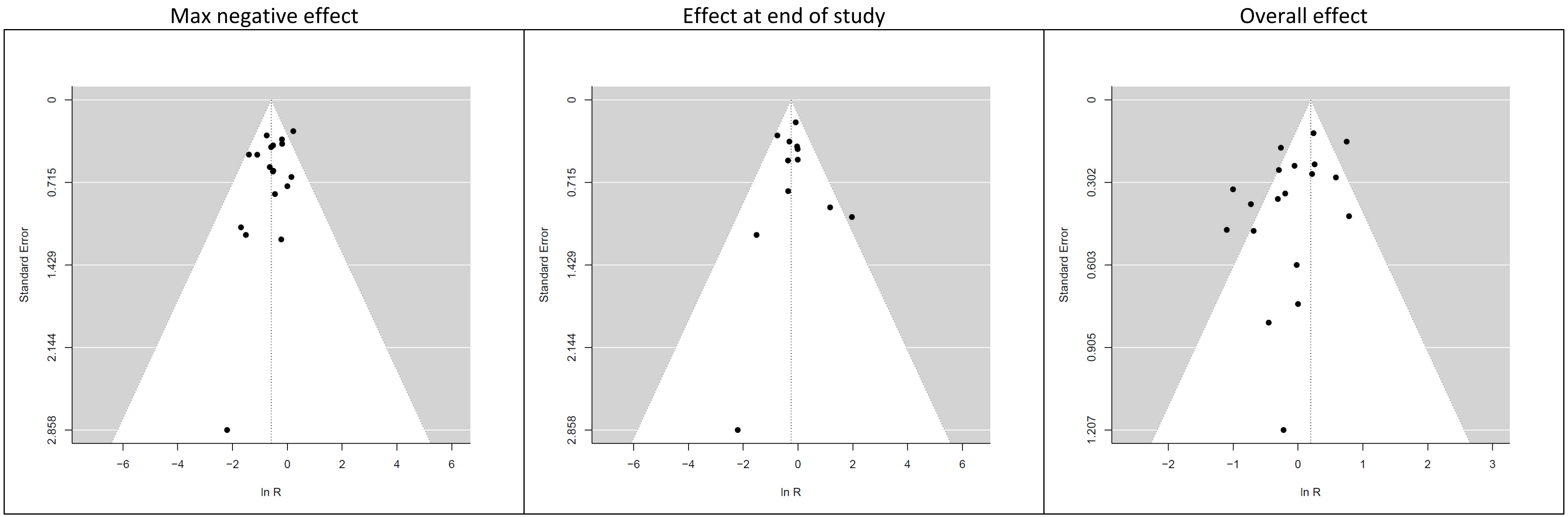

Figure S15. Funnel plots for effect sizes of crustacea abundance. There is no obvious asymmetry that could indicate possible publication bias, except for the overall effect where nonsignificant positive effects may be missing.

Funnel plots, taxa richness

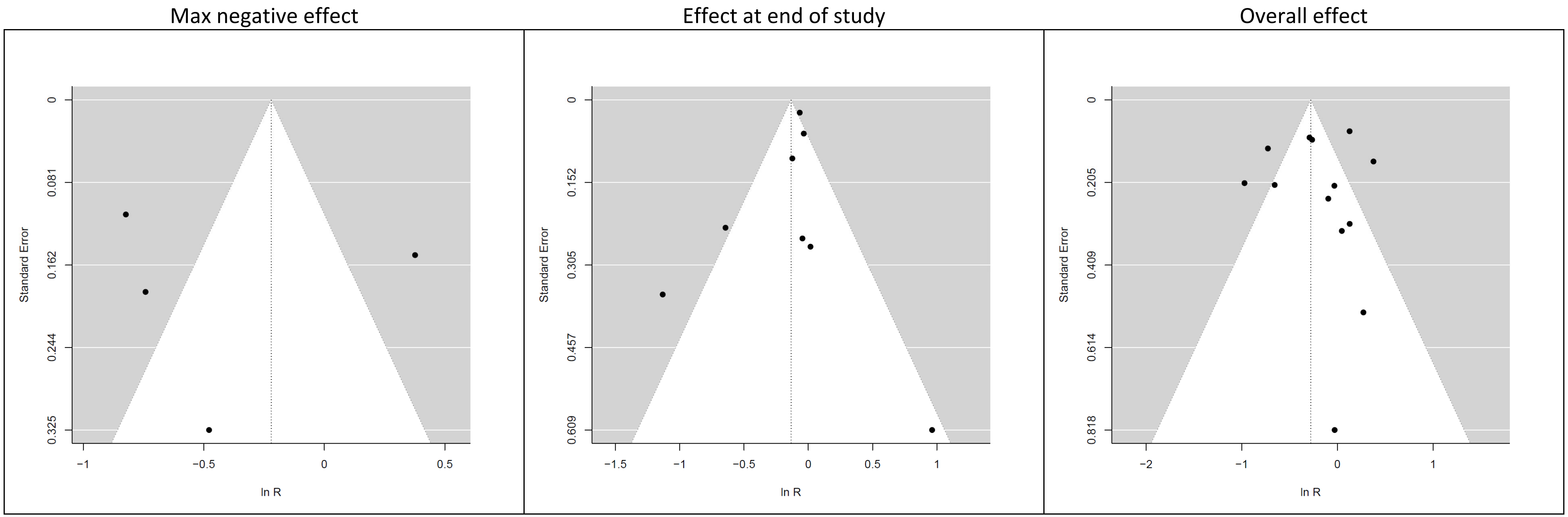

Figure S16. Funnel plots for effect sizes of taxa richness. There is no obvious asymmetry that could indicate possible publication bias, except for the overall effect where nonsignificant negative effects may be missing.

Regression plots, chironomid emergence

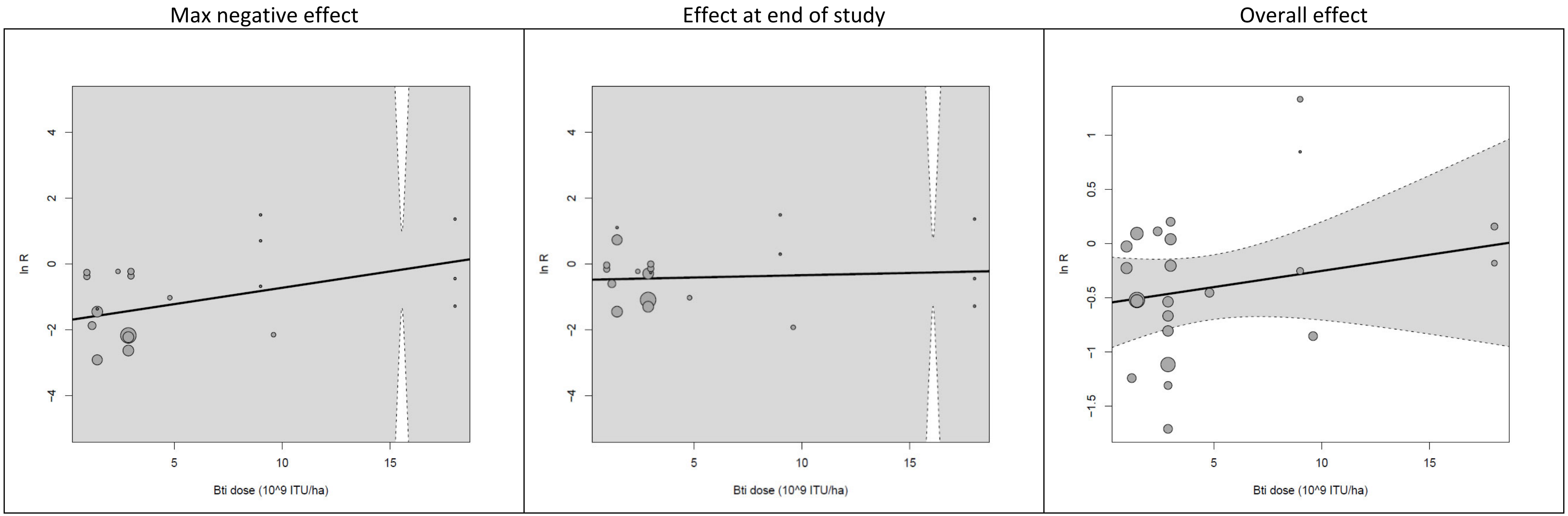

Figure S17. Effect sizes as a function of Bti dose in 10^9 ITU/ha.

Table S25. Slope and p-value for regression lines where the moderator is Bti dose.

Group	Summary effect	Slope	p-value	n
chironomid	Max negative	0.099	0.99	20
chironomid	End of study	0.014	1	20
chironomid	Overall	0.030	0.35	23

Regression plots, chironomid abundance

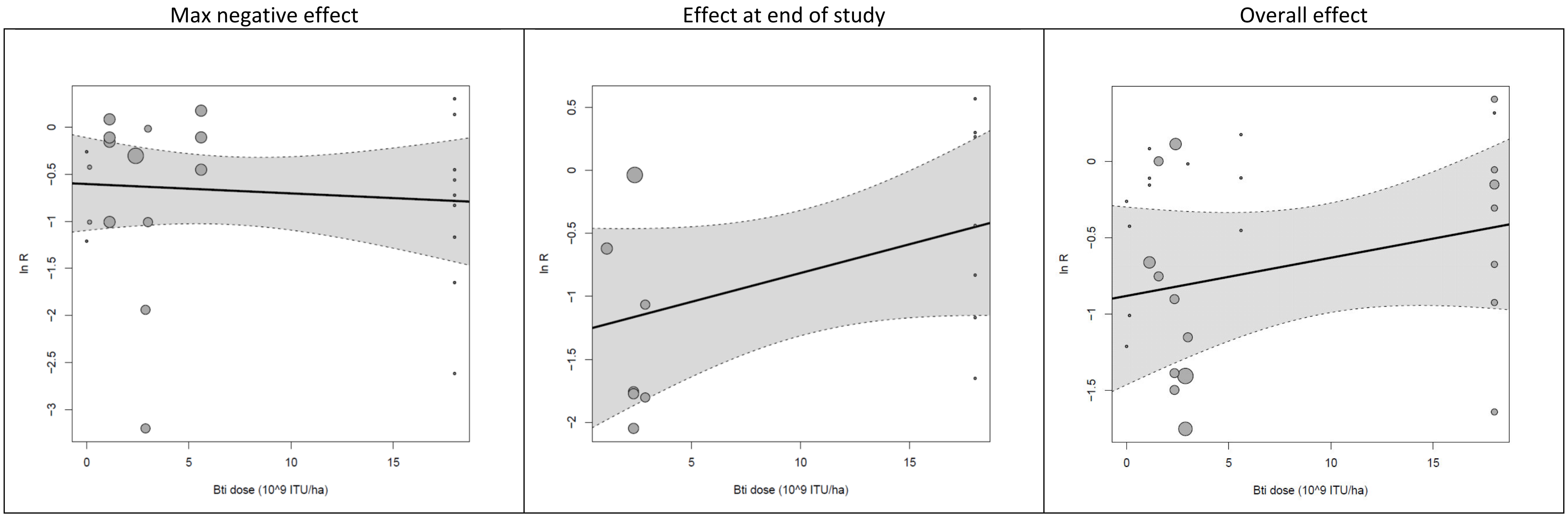

Figure S18. Effect sizes as a function of Bti dose in 10^9 ITU/ha.

Table S26. Slope and p-value for regression lines where the moderator is Bti dose.

Group	Summary effect	Slope	p-value	n
chironomid	Max negative	-0.01	0.68	25
chironomid	End of study	0.046	0.14	14
chironomid	Overall	0.025	0.29	29

Regression plots, crustacea abundance

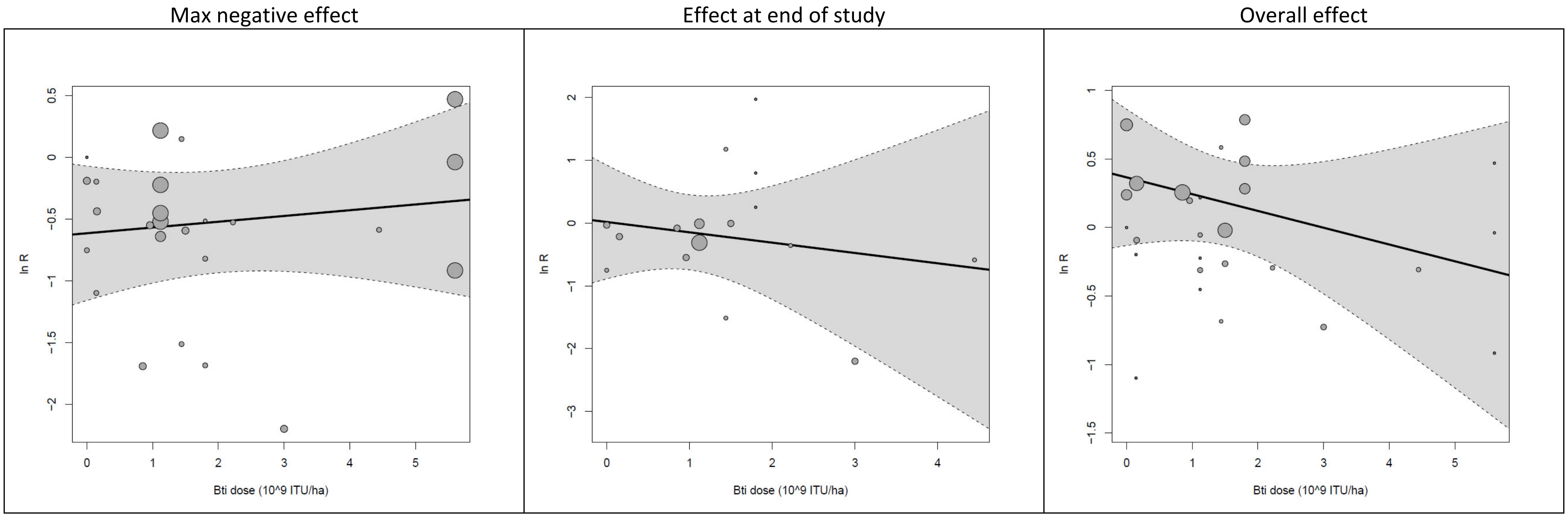

Figure S19. Effect sizes as a function of Bti dose in 10^9 ITU/ha.

Table S27. Slope and p-value for regression lines where the moderator is Bti dose.

Group	Summary effect	Slope	p-value	n
crustacea	Max negative	0.047	0.59	25
crustacea	End of study	-0.16	0.61	16
crustacea	Overall	-0.12	0.32	27

Regression plots, taxa richness

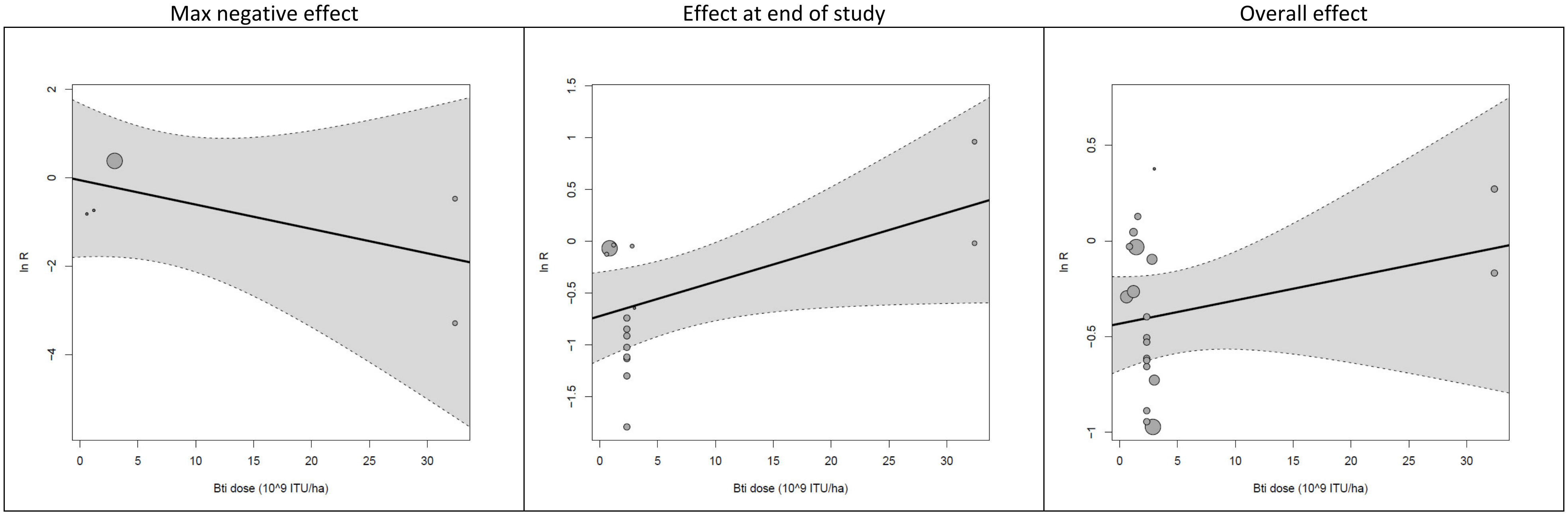

Figure S20. Effect sizes as a function of Bti dose in 10^9 ITU/ha.

Table S28. Slope and p-value for regression lines where the moderator is Bti dose.

Group	Summary effect	Slope	p-value	n
taxa richness	Max negative	-0.055	0.27	5
taxa richness	End of study	0.033	0.055	15
taxa richness	Overall	0.012	0.33	20

Sensitivity analysis, Chironomidae abundance

Max negative effect | Max negative effect

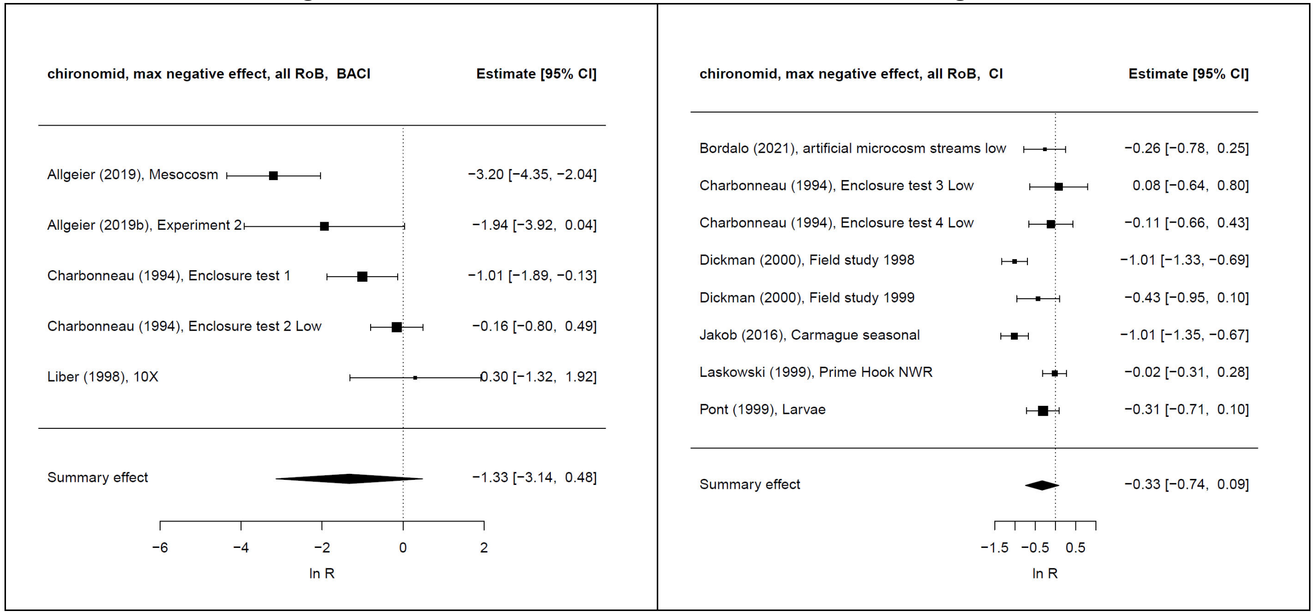

Figure S21. Forest plots showing maximum negative effects on chironomid abundance in Bti treated areas compared to untreated areas. To the left are studies with a BACI design, to the right are studies with a CI design.

Table S29. Meta-analysis results for studies with BACI and CI designs.

RoB	Group	Summary effect	Estimate [95% CI]	p-value	n	Change (%)	I^2 (%)	Fail-safe N
all	BACI	max negative effect	-1.33 [-3.14, 0.482]	0.111	5	-74 [-96 – 62]	85	--
all	CI	max negative effect	-0.326 [-0.74, 0.0874]	0.104	8	-28 [-52 – 9.1]	83	--

Sensitivity analysis, Crustacea abundance

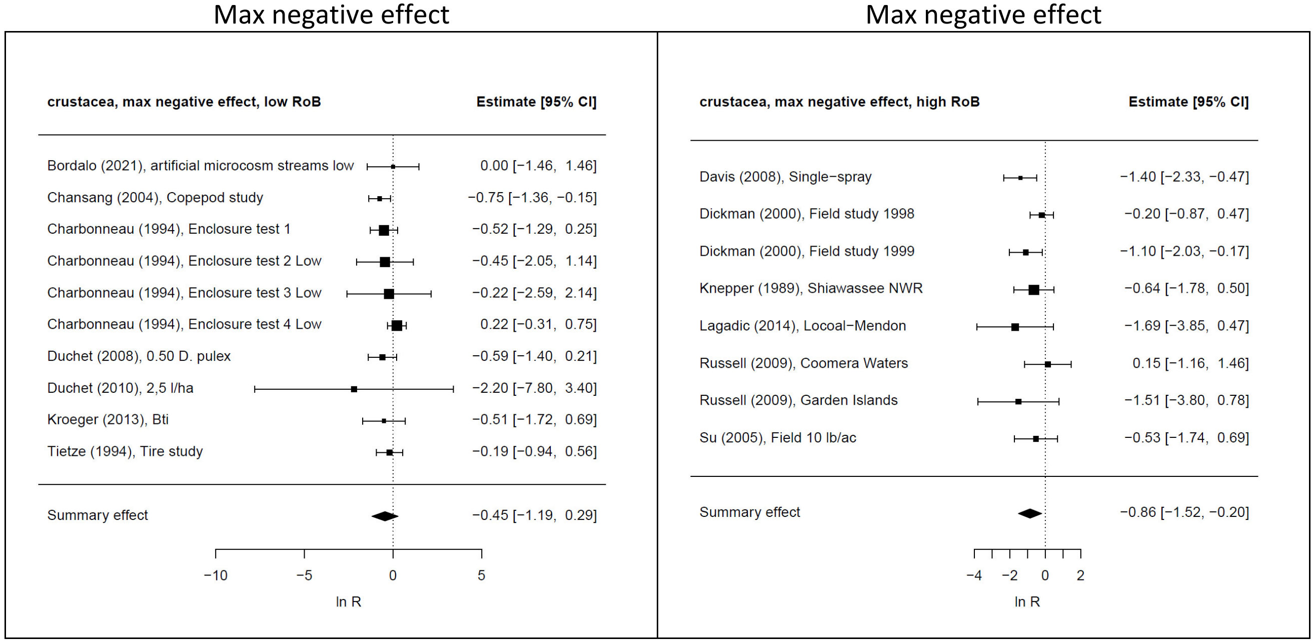

Figure S22. Forest plots showing maximum negative effects on crustacea abundance in Bti treated areas compared to untreated areas. To the left are studies judged to have low, probably low, or moderate risk of bias included (p>0.05), to the right are studies judged to have high, probably high, or unclear risk of bias included (p<0.05)

Table S30. Meta-analysis results for studies with low, probably low, or moderate risk of bias (RoB=low) and studies with high, probably high, or unclear risk of bias (RoB=high).

RoB	Group	Summary effect	Estimate [95% CI]	p-value	n	Change (%)	I^2 (%)	Fail-safe N
low	crustacea	Max negative	-0.451 [-1.19, 0.289]	0.201	10	-36 [-70 – 34]	0	--
high	crustacea	Max negative	-0.856 [-1.52, -0.197]	**0.0181**	8	-58 [-78 – -18]	9.9	18

Sensitivity analysis, invertebrate taxa richness

Overall effect | Overall effect | Overall effect

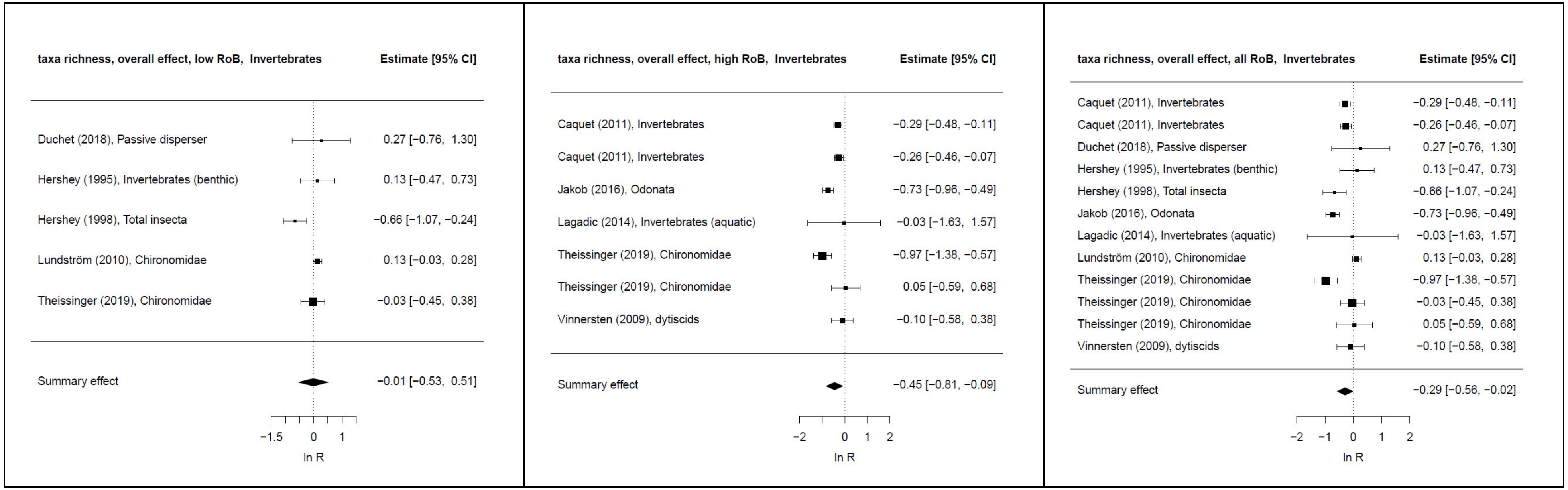

Figure S23. Forest plots showing overall effects across study periods on invertebrate taxa richness in Bti treated areas compared to untreated areas. To the left are studies judged to have low, probably low, or moderate risk of bias included (p>0.05), in the centre are studies judged to have high, probably high, or unclear risk of bias included (p<0.05), and to the right are all studies included (p<0.05).

Table S31. Meta-analysis results for studies with low, probably low, or moderate risk of bias (RoB=low) and studies with high, probably high, or unclear risk of bias (RoB=high). The results for RoB=all are equal to those for the subgroup shown in Figure S5.

RoB	Group	Summary effect	Estimate [95% CI]	p-value	n	Change (%)	I^2 (%)	Fail-safe N
low	taxa richness	overall effect	-0.0148 [-0.535, 0.505]	0.941	5	-1.5 [-41 – 66]	0	--
high	taxa richness	overall effect	-0.446 [-0.805, -0.0869]	**0.0228**	7	-36 [-55 – -8.3]	78	92
all	taxa richness	overall effect	-0.289 [-0.559, -0.0189]	**0.0382**	12	-25 [-43 – -1.9]	53	78

Articles used in meta-analyses

Short reference	Reference
Allgeier (2019)	Allgeier, S., Kaestel, A., Bruehl Carsten, A., 2019b. Adverse effects of mosquito control using Bacillus thuringiensis var. israelensis: Reduced chironomid abundances in mesocosm, semi-field and field studies. Ecotoxicology and Environmental Safety, 169: 786-796.
Allgeier (2019b)	Allgeier, S., Friedrich, A., Brühl, C.A., 2019a. Mosquito control based on Bacillus thuringiensis israelensis (Bti) interrupts artificial wetland food chains. Science of the Total Environment, 686: 1173-1184.
Bordalo (2021)	Bordalo Maria, D. et al., 2021. Responses of benthic macroinvertebrate communities to a Bti-based insecticide in artificial microcosm streams. Environmental Pollution, 282.
Brown (1999)	Brown, M.D., Thomas, D., Mason, P., Greenwood, J.G., Kay, B.H., 1999. Laboratory and field evaluation of the efficacy of four insecticides for Aedes vigilax (Diptera : Culicidae) and toxicity to the nontarget shrimp Leander tenuicornis (Decapoda : Palaemonidae). Journal of Economic Entomology, 92(5): 1045-1051.
Caquet (2011)	Caquet, T., Roucaute, M., Le, G., Pierre, Lagadic, L., 2011. Effects of repeated field applications of two formulations of Bacillus thuringiensis var. israelensis on non-target saltmarsh invertebrates in Atlantic coastal wetlands. Ecotoxicology and Environmental Safety, 74(5): 1122-1130.
Chansang (2004)	Chansang, U.R., Bhumiratana, A., Kittayapong, P., 2004. Combination of Mesocyclops thermocyclopoides and Bacillus thuringiensis var. israelensis: A better approach for the control of Aedes aegypti larvae in water containers. Journal of Vector Ecology, 29(2): 218-226.
Charbonneau (1994)	Charbonneau, C.S., Drobney, R.D., Rabeni, C.F., 1994. EFFECTS OF BACILLUS-THURINGIENSIS VAR ISRAELENSIS ON NONTARGET BENTHIC ORGANISMS IN A LENTIC HABITAT AND FACTORS AFFECTING THE EFFICACY OF THE LARVICIDE. Environmental Toxicology and Chemistry, 13(2): 267-279.
Davis (2008)	Davis Ryan, S., Peterson Robert, K.D., 2008. Effects of single and multiple applications of mosquito insecticides on nontarget arthropods. Journal of the American Mosquito Control Association, 24(2): 270-280.
Dickman (2000)	Dickman, M., 2000. Impacts of a mosquito selective pesticide, Bti, on the macroinvertebrates of a subtropical stream in Hong Kong. Chemosphere, 41(1-2): 209-217.
Duchet (2008)	Duchet, C. et al., 2008. Effects of spinosad and Bacillus thuringiensis israelensis on a natural population of Daphnia pulex in field microcosms. Chemosphere, 74(1): 70-77.

Duchet (2010)	Duchet, C., Caquet, T., Franquet, E., Lagneau, C., Lagadic, L., 2010a. Influence of environmental factors on the response of a natural population of Daphnia magna (Crustacea: Cladocera) to spinosad and Bacillus thuringiensis israelensis in Mediterranean coastal wetlands. Environmental Pollution, 158(5): 1825-1833.
Duchet (2015)	Duchet, C., Franquet, E., Lagadic, L., Lagneau, C., 2015. Effects of Bacillus thuringiensis israelensis and spinosad on adult emergence of the non-biting midges Polypedilum nubifer (Skuse) and Tanytarsus curticornis Kieffer (Diptera: Chironomidae) in coastal wetlands. Ecotoxicology and Environmental Safety, 115: 272-278.
Duchet (2018)	Duchet, C. et al., 2018. Pesticide-mediated trophic cascade and an ecological trap for mosquitoes. Ecosphere, 9(4).
Duguma (2015)	Duguma, D. et al., 2015. Microbial communities and nutrient dynamics in experimental microcosms are altered after the application of a high dose of Bti. Journal of Applied Ecology, 52(3): 763-773.
Gutierrez (2017)	Gutierrez, Y., Ramos Gabryele, S., Tome Hudson, V.V., Oliveira Eugenio, E., Salaro Ana, L., 2017. Bt(i)-based insecticide enhances the predatory abilities of the backswimmer Buenoa tarsalis (Hemiptera: Notonectidae). Ecotoxicology, 26(8): 1147-1155.
Hanowski (1997)	Hanowski, J.M., Niemi, G.J., Lima, A.R., Regal, R.R., 1997a. Do mosquito control treatments of wetlands affect red-winged blackbird (Agelaius phoeniceus) growth, reproduction, or behavior? Environmental Toxicology and Chemistry, 16(5): 1014-1019.
Hershey (1995)	Hershey, A.E., Shannon, L., Axler, R., Ernst, C., Mickelson, P., 1995. EFFECTS OF METHOPRENE AND BTI (BACILLUS-THURINGIENSIS VAR ISRAELENSIS) ON NONTARGET INSECTS. Hydrobiologia, 308(3): 219-227.
Hershey (1998)	Hershey, A.E., Lima, A.R., Niemi, G.J., Regal, R.R., 1998. Effects of Bacillus thuringiensis israelensis (Bti) and methoprene on nontarget macroinvertebrates in Minnesota wetlands. Ecological Applications, 8(1): 41-60.
Jakob (2016)	Jakob, C., Poulin, B., 2016. Indirect effects of mosquito control using Bti on dragonflies and damselflies (Odonata) in the Camargue. Insect Conservation and Diversity, 9(2): 161-169.
Knepper (1989)	Knepper, R.G., Walker, E.D., 1989. EFFECT OF BACILLUS-THURINGIENSIS-ISRAELENSIS (H-14) ON THE ISOPOD ASELLUS-FORBESI AND SPRING AEDES MOSQUITOS IN MICHIGAN. Journal of the American Mosquito Control Association, 5(4): 596-598.
Kroeger (2013)	Kroeger, I., Liess, M., Dziock, F., Duquesne, S., 2013b. Sustainable control of mosquito larvae in the field by the combined actions of the biological insecticide Bti and natural competitors. Journal of Vector Ecology, 38(1): 82-89.
Lagadic (2014)	Lagadic, L., Roucaute, M., Caquet, T., 2014. Bti sprays do not adversely affect non-target aquatic invertebrates in French Atlantic coastal wetlands. Journal of Applied Ecology, 51(1): 102-113.
Laskowski (1999)	Laskowski, H.P., Larsen, A.C., O'Shea, G.F., Pittendrigh, B.R., 1999. Effect of xanthan gum and traditional mosquito larvicides on chironomid larvae. Wildlife Society Bulletin, 27(3): 741-745.
Liber (1998)	Liber, K., Schmude, K.L., Rau, D.M., 1998. Toxicity of Bacillus thuringiensis var. israelensis to chironomids in pond mesocosms. Ecotoxicology, 7(6): 343-354.

Lundström (2010)	Lundstrom, J.O., Brodin, Y., Schafer, M.L., Vinnersten, T.Z.P., Ostman, O., 2010a. High species richness of Chironomidae (Diptera) in temporary flooded wetlands associated with high species turn-over rates. Bulletin of Entomological Research, 100(4): 433-444.
Lundström (2010b)	Lundstrom, J.O. et al., 2010b. Production of wetland Chironomidae (Diptera) and the effects of using Bacillus thuringiensis israelensis for mosquito control. Bulletin of Entomological Research, 100(1): 117-125.
Ostman (2008)	Ostman, O., Lundstrom Jan, O., Vinnersten Thomas, Z.P., 2008. Effects of mosquito larvae removal with Bacillus thuringiensis israelensis (Bti) on natural protozoan communities. Hydrobiologia, 607: 231-235.
Painter (1996)	Painter, M.K., Tennessen, K.J., Richardson, T.D., 1996. Effects of repeated applications of Bacillus thuringiensis israelensis on the mosquito predator Erythemis simplicicollis (Odonata: Libellulidae) from hatching to final instar. Environmental Entomology, 25(1): 184-191.
Pont (1999)	Pont, D., Franquet, E., Tourenq, J.N., 1999. Impact of different Bacillus thuringiensis variety israelensis treatments on a chironomid (Diptera Chironomidae) community in a temporary marsh. Journal of Economic Entomology, 92(2): 266-272.
Poulin (2018)	Poulin, B., Lefebvre, G., 2018. Perturbation and delayed recovery of the reed invertebrate assemblage in Camargue marshes sprayed with Bacillus thuringiensis israelensis. Insect Science, 25(4): 542-548.
Russell (2009)	Russell Tanya, L., Kay Brian, H., Skilleter Greg, A., 2009. Environmental effects of mosquito insecticides on saltmarsh invertebrate fauna. Aquatic Biology, 6(1-3): 77-90.
Strasburg (2021)	Strasburg, M., Boone Michelle, D., 2021. Effects of Trematode Parasites on Snails and Northern Leopard Frogs (Lithobates pipiens) in Pesticide-Exposed Mesocosm Communities. Journal of Herpetology, 55(3): 229-236.
Su (1999)	Su, T.Y., Mulla, M.S., 1999b. Microbial agents Bacillus thuringiensis ssp israelensis and Bacillus sphaericus suppress eutrophication, enhance water quality, and control mosquitoes in microcosms. Environmental Entomology, 28(4): 761-767.
Su (2005)	Su, T.Y., Mulla, M.S., 2005. Toxicity and effects of microbial mosquito larvicides and larvicidal oil on the development and fecundity of the tadpole shrimp Triops newberryi (Packard) (Notostraca : Triopsidae). Journal of Vector Ecology, 30(1): 107-114.
Theissinger (2018)	Theissinger, K. et al., 2018. Using DNA metabarcoding for assessing chironomid diversity and community change in mosquito controlled temporary wetlands. MBMG Metabarcoding and Metagenomics: 672-677.
Theissinger (2019)	Theissinger, K. et al., 2019. Mosquito control actions affect chironomid diversity in temporary wetlands of the Upper Rhine Valley. Molecular Ecology, 28(18): 4300-4316.
Tietze (1994)	Tietze, N.S., Hester, P.G., Shaffer, K.R., Prescott, S.J., Schreiber, E.T., 1994. INTEGRATED MANAGEMENT OF WASTE TIRE MOSQUITOS UTILIZING MESOCYCLOPS-LONGISETUS (COPEPODA, CYCLOPIDAE), BACILLUS-THURINGIENSIS VAR ISRAELENSIS, BACILLUS-SPHAERICUS, AND METHOPRENE. Journal of the American Mosquito Control Association, 10(3): 363-373.
Vinnersten (2009)	Vinnersten Thomas, Z.P., Lundstrom Jan, O., Petersson, E., Landin, J., 2009. Diving beetle assemblages of flooded wetlands in relation to time, wetland type and Bti-based mosquito control. Hydrobiologia, 635(1): 189-203.

References

Rosenberg, M. S. (2005). The file-drawer problem revisited: A general weighted method for calculating fail-safe numbers in meta-analysis. Evolution, 59(2), 464–468. https://doi.org/10.1111/j.0014-3820.2005.tb0
